# Assessing appropriateness of antibiotic therapy: a scoping review of definitions and their clinical implication

**DOI:** 10.1007/s15010-026-02733-x

**Published:** 2026-01-31

**Authors:** Lea A. Nikolai, Beryl P. Gladstone, Arisa Hakariya, Marissa Rink, Evelina Tacconelli, Siri Göpel

**Affiliations:** 1 Division of Infectious Diseases, Department of Internal Medicine I, University Hospital Tübingen, Otfried Müller Str 12, Tübingen, Germany; 2 DZIF German Centre for Infection Research, Brunswick, Germany; 3 Division of Infectious Diseases, Department of Diagnostic and Public Health, University of Verona, Policlinico GB Rossi, Verona, Italy

**Keywords:** Antimicrobial stewardship, Appropriate antibiotic therapy, Quality indicators, Scoping review, Antibiotic susceptibility

## Abstract

**Purpose:**

Appropriate antibiotic therapy (AAT) is associated with improved clinical outcomes, yet definitions used to assess AAT vary widely and focus predominantly on in-vitro susceptibility. This contrasts with antimicrobial stewardship (AMS) principles, which emphasize additional factors such as infection focus, dosing, route, and treatment duration. We conducted a scoping review to describe how AAT is defined in clinical research and to examine the association between AAT definitions and patient outcomes.

**Methods:**

We included observational studies published between 2011 and 2021 evaluating the impact of AAT on outcomes in hospitalized adults with bacterial infections. Definitions of empiric and definite AAT were extracted and categorized according to the aspects considered. Association between definition characteristics and reported outcomes were analysed.

**Results:**

Among 288 included studies, nearly all (98.6%) provided a definition of AAT. Most definitions relied on in-vitro susceptibility alone for empiric (40.7%, 105/258) and definite (31.7%, 13/41) therapy. Other aspects (e.g. dosing, duration, guideline adherence) were used inconsistently and with heterogeneous operationalization. Details on AAT assessment were frequently missing for several aspects, including dosing and route of administration. Empiric AAT was associated with improved outcomes in 62.8% (125/199), particularly for long-term and ICU mortality. However, the associations did not differ consistently by the type or complexity of AAT definition, except when minimum treatment duration was included.

**Conclusion:**

AAT definitions in current clinical research remain largely susceptibility-based and incompletely aligned with AMS frameworks. More standardized, multidimensional definitions that incorporate clinical, pharmacological, and stewardship criteria are needed to enable comparability across studies and to better understand the true impact of AAT on patient outcomes.

**Supplementary Information:**

The online version contains supplementary material available at 10.1007/s15010-026-02733-x.

## Introduction

Appropriateness of antibiotics is a key factor in effective treatment of bacterial infections. Multiple systematic reviews found that appropriate antibiotic therapy (AAT) significantly reduces mortality and may have positive effects on additional patient outcomes among patients with sepsis and severe bacterial infection [1–4]. Susceptibility-based definitions of AAT, referring to the causative pathogen and its resistance phenotype determined in microbiological susceptibility testing, are the established standard in defining AAT in clinical studies [5, 6]. In this context, appropriateness of empiric treatment is usually assessed retrospectively based on antibiograms available only after start of antibiotic treatment. However, an optimal antibiotic therapy additionally requires a conscious choice of agent, dosing, route of administration and length of therapy [7]. Further, susceptibility-based definitions of AAT fail to detect unnecessary or overly broad antibiotics, which are associated with adverse events and contribute to the development of antimicrobial resistance [8].

These other aspects of AAT such as timely initiation of antibiotics or dosing are investigated less frequently [2–4]. In the past, various authors highlighted the shortcomings of centering definitions of AAT around in vitro susceptibility only and called for comprehensive, uniform assessment of AAT [5, 6, 9]. Recent publications have therefore focused on developing quality indicators (QIs) for AAT, often in the context of antimicrobial stewardship (AMS) [10–14]. It remains unclear to what extent these recommendations have been implemented in research. In addition, the impact of varying AAT definitions on the measured effect of AAT on patient outcomes requires further investigation. Previous reviews suggest that the definition applied impacts the effect measure; however, evidence is not conclusive [1, 3]. Another major challenge in measuring the effect of AAT on patient outcomes is the investigation of potential confounding variables such as disease severity which may impact both choice of therapy and patient outcomes [6].

With the aim to investigate the definition of AAT as well as the effect of AAT on outcomes in hospitalized adults treated for bacterial infections, we mapped the current state of defining AAT in research to identify its patterns. Further, we investigated how varying definition of AAT impact outcome analysis.

## Methods

A systematic literature search for studies evaluating the effect of AAT on patient outcomes in adult patients with confirmed or suspected bacterial infections treated in the inpatient setting was conducted. Methodology was in accordance with the scoping review framework [15] and PRISMA-ScR guidelines (supplementary Table S1) [16]. The registered protocol can be found here (https://osf.io/zyngd) [17].

### Search strategy and data extraction

We conducted the literature search on November 24, 2021 in PubMed, Web of Science and Cochrane Library using search terms related to antibiotic therapy, appropriate or adequate and outcome measures (search terms in supplements p. 9). A non-blinded two-step screening was performed by one reviewer (LN) and discrepancies sorted with another reviewer (PB). The data was extracted by two reviewers (LN and AH), and any discrepancies clarified (PB). Extracted variables included study characteristics, study inclusion criteria such as study setting, pathogen, type of infection and acquisition, the definition(s) of AAT applied, and all available outcomes of interest. Variables were extracted into a REDCap database [18, 19].

### Eligibility criteria

Retrospective and prospective observational studies with control groups published after 2011, evaluating the impact of appropriate or adequate antibiotic therapy on any patient outcomes and/or emergence of antibiotic resistance, were included. Reviews, case reports or conference contributions were excluded. Inclusion was limited to English language. Exclusion criteria included studies evaluating prophylactic antibiotic use, studies conducted in dental care or outpatient settings, nursing homes and long-term care facilities, pediatric cohorts, cohorts with over 10% percent of patients infected with a non-bacterial pathogen, cohorts focusing on COVID-19 or HIV only.

### Definition of AAT

Recent literature suggesting QIs for AAT was used to create a predefined list of potential aspects used in AAT definition [14, 20]. Each study definition was checked for the following aspects: in-vitro susceptibility, timely initiation of therapy, dosing, route of administration, aminoglycoside restriction, duration of therapy, guideline-based choice of therapy, expert assessment, diagnostic procedures (such as taking blood cultures), adequate documentation of therapy in patient notes, antibiotic de-escalation, switch to oral therapy when feasible and process-related measures (such as bedside consultation). Any additional aspects reported were also extracted. We investigated the factors influencing AAT definition based on individual aspects of AAT as well as grouped AAT definitions as follows: studies defining appropriateness by susceptibility only, by susceptibility and one other aspect (with a subcategory of susceptibility and timely initiation of therapy), by susceptibility and at least two other aspects and studies defining AAT non-susceptibility centered (such as guideline-based definitions). Susceptibility refers here to therapy based on individual in-vitro susceptibility testing. Other definitions and detailed methods are provided in the appendix p. 6.

### Outcomes related to AAT

All patient outcomes reported in relation to AAT were included. Details on the direction of association (AAT or inappropriate antibiotic therapy (IAT) associated with favorable outcome), type of analysis (univariable or multivariable), and whether a significant effect (*p* < 0.05) was found, were extracted. All-cause mortality was classified as early (2–15 days), late (21–30 days) or long-term (> 30 days to 1-year) mortality. Overall mortality without specified time period was considered as in-hospital mortality. Clinical failure was summarized along with clinical success after harmonizing the direction of association (details provided in supplements p. 11). Potential risk factors reported in univariable and multivariable analyses other than AAT were studied for their effect on outcomes of interest.

### Statistical tests

The association of study characteristics with the definitions as well as the impact of definitions on outcomes were tested using Chi square statistic or Fisher’s exact test, whichever applicable. All statistical analyses were performed using Stata/SE 15 [21].

## Results

### Study selection and study characteristics

**Fig. 1:**
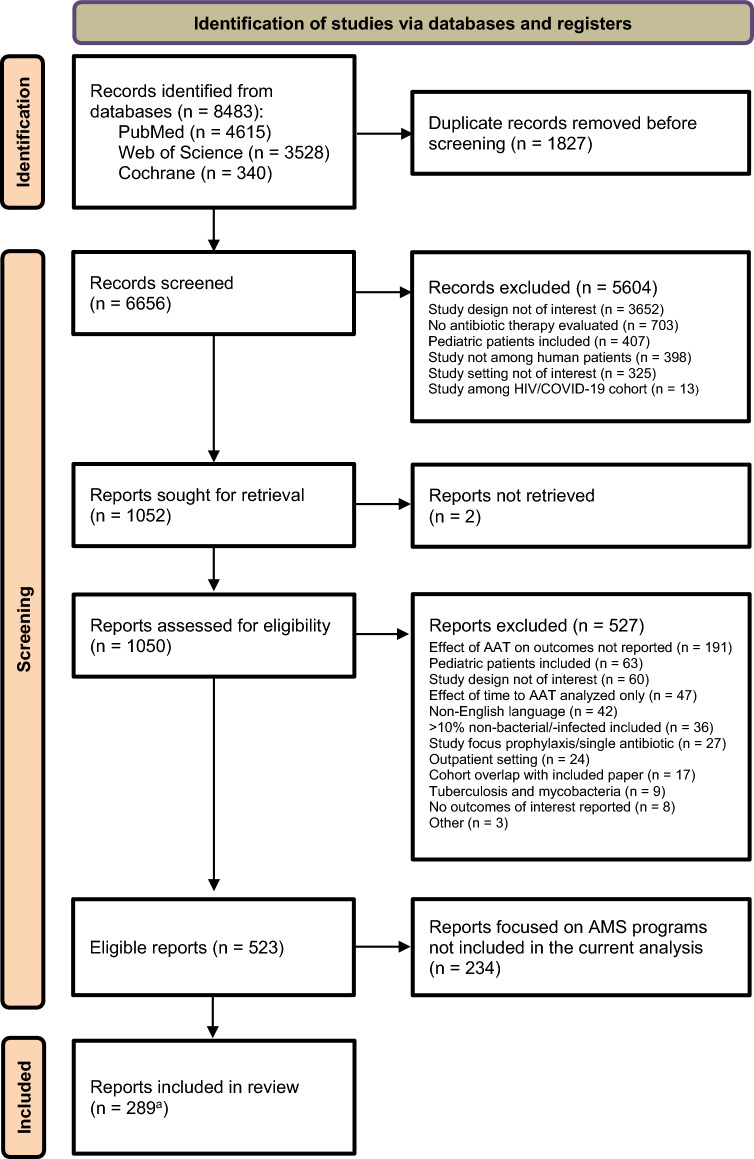
PRISMA 2020 flow diagram showing the process of study selection A one additional publication based on the same study cohort and methods was used to extract a second outcome for one study and hence 288 studies are considered as the number of included studies

Our search identified 6553 unique studies, of which 5501 were excluded through title and abstract screening. 1052 full-text publications were screened and 288 were included (Fig. 1). In total, 310,096 patients with a median age of 63.1 years (ranging from 38.9 to 84.3) were included. Main study characteristics are presented in Table 1 (see supplementary Table S2 for full list of studies).

**Table 1:** Characteristics of included studies

Variable	Studies (*n* = 288)
Median number of patients per study [IQR]	251 [113;549.5]
Median age of included patients [IQR]^a^	63.1 [58.7;68]
Year of publication	
2011–2014	101 (35.07%)
2015–2018	130 (45.14%)
2019–2021	57 (19.79%)
Study design	
Retrospective cohort study	176 (61.11%)
Prospective cohort study	77 (26.74%)
Secondary data from prospective cohort studies	15 (5.21%)
Retrospective case cohort data based on case control studies	12 (4.17%)
Secondary data from interventional studies	4 (1.39%)
Others	4 (1.39%)
Multicenter study	104 (36.11%)
Study department	
Hospital wide	176 (61.11%)
ICU	73 (25.35%)
Emergency department	14 (4.86%)
Mixed	23 (7.99%)
Unclear	2 (0.69%)
Target site of acquisition in included studies	
Healthcare-setting	53 (18.40%)
Acquisition outside the hospital	45 (15.62%)
Inclusion not restricted by site of acquisition	190 (65.97%)
Target pathogens in included studies	
* P. aeruginosa*	20 (6.94%)
* E. coli*	6 (2.08%)
* S. aureus*	18 (6.25%)
* K. pneumoniae*	19 (6.60%)
* A. baumannii*	14 (4.86%)
* S. pneumoniae*	3 (1.04%)
Gram negative bacteria	21 (7.29%)
* Enterobacteriaceae* spp.	16 (5.56%)
Mixed specific pathogens	38 (13.19%)
Other	5 (1.74%)
Inclusion not restricted by infecting pathogen	128 (44.44%)
Resistance mechanisms of target pathogens in included studies	
Carbapenem-resistant bacteria	21 (7.29%)
ESBL-producing bacteria	14 (4.86%)
MRSA	5 (1.74%)
VRE	1 (0.35%)
Unspecified	3 (1.04%)
Inclusion not restricted by resistance mechanism	244 (84.72%)
Target type of infection in included studies	
Bloodstream infection	187 (64.93%)
Respiratory infection	45 (15.62%)
Urinary tract infection	11 (3.82%)
Abdominal infection	8 (2.78%)
Endocarditis	5 (1.74%)
Skin and soft tissue infection	4 (1.39%)
Central nervous system infection	2 (0.69%)
Mixed / Other	7 (2.43%)
Inclusion not restricted by type of infection	19 (6.60%)
Phases of antibiotic treatment investigated	
Empirical	255 (88.54%)
Definite	41 (16.08%)
General / both	7 (2.43%)
Unknown	15 (5.21%)
Outcome measure^b^	
Mortality	265 (92.01%)
Length of stay	35 (12.15%)
Clinical failure	25 (8.68%)
ICU length of stay	15 (5.21%)
Rate of ICU admission	8 (2.78%)
Adverse events	7 (2.43%)
Rate of hospital readmission	6 (2.08%)
Duration of mechanical ventilation	6 (2.08%)
Other^c^	7 (2.43%)

IQR, Interquartile range; ICU, Intensive care unit; ESBL, Extended-spectrum beta-lactamase; MRSA, Methicillin-resistant *Staphylococcus aureus*; VRE, Vancomycin-resistant enterococci; AAT, appropriate antibiotic therapy

^a^Based on 192 studies reporting mean/median patient age

^b^One study can provide multiple outcomes

^c^Other outcomes include: rate of *C. difficile* infection, duration of antibiotic treatment, emergence of multidrug resistance, mechanical ventilation, microbiological failure, time to clinical success

### Definition of appropriateness

In total, 321 definitions of AAT were extracted from 284 studies: 258 definitions of empirical AAT, 41 definitions of definite AAT, 5 definitions of AAT during the entire treatment course, 3 definitions of AAT regarding other treatment phases and 14 definitions relating to unclear treatment phases (overview provided in supplementary Table S3). Four studies (1.39%) investigated the effect of AAT on outcomes without defining AAT. Supplementary Table S4 provides the study definitions of empiric and definite therapy.

The most frequent aspect integrated in definitions of both empiric and definite AAT was in-vitro susceptibility (Table 2). Empiric and definite AAT were defined based on susceptibility only among 105 (40.70%) and 13 (31.71%) studies, respectively. Though only used in few studies (13 (5.04%) empiric and 5 (12.20%) definite AAT), non-susceptibility-centered definitions formed a heterogenous group, with some studies considering only explicitly specified antibiotics appropriate in their study setting, mainly when inclusion was limited to one pathogen. The most frequent combinations of individual aspects for empiric AAT were susceptibility and timing (46 definitions, 17.83%), susceptibility, dosing and route of administration (11 studies, 4.26%), and susceptibility, timing, dosing and route (10 studies, 3.88%). For definite therapy, these were susceptibility and duration of therapy (4 studies, 9.76%), susceptibility and aminoglycoside restriction (3 studies, 7.32%) and susceptibility and dosing (3 studies, 7.32%). There was no relevant change in use of AAT aspects over time.

**Table 2:** Overview of the aspects included in study definitions of appropriate antibiotic therapy (AAT)

Aspect	Number of definitions^a^	Number of definitions with details on evaluation
Total number of definitions of empirical AAT	258	
Susceptibility	245 (94.96%)	151 (61.63%)
Timing	92 (35.66%)	91 (98.91%)
Dosing	49 (18.99%)	21 (42.86%)
Route of administration	34 (13.18%)	14 (41.18%)
Aminoglycoside restriction	16 (6.20%)	16 (100.00%)
Duration	11 (4.26%)	10 (90.91%)
Guideline-based choice of antibiotic agent	11 (4.26%)	11 (100.00%)
Other	33 (12.79%)	33 (100.00%)
Total number of definitions of definite AAT	41	
Susceptibility	36 (87.81%)	31 (86.11%)
Timing	4 (9.76%)	4 (100.00%)
Dosing	10 (24.39%)	7 (70.00%)
Route of administration	5 (12.20%)	2 (40.00%)
Aminoglycoside restriction	5 (12.20%)	5 (100.00%)
Duration	6 (14.63%)	6 (100.00%)
Guideline-based choice of antibiotic agent	2 (4.88%)	2 (100.00%)
Other	9 (21.95%)	9 (100.00%)
Total number of definitions of general AAT	5	
Susceptibility	3 (60.00%)	1 (33.33%)
Timing	1 (20.00%)	1 (100.00%)
Dosing	2 (40.00%)	1 (50.00%)
Route of administration	1 (20.00%)	1 (100.00%)
Aminoglycoside restriction	–	–
Duration	2 (40.00%)	2 (100.00%)
Guideline-based choice of antibiotic agent	3 (60.00%)	3 (100.00%)
Other	3 (60.00%)	3 (100.00%)

^a^One definition may contain multiple aspects of AAT

AAT, appropriate antibiotic therapy

Methods of how studies assessed adherence to aspects of AAT were inconsistently reported (Table 2, supplementary tables S5, S6 and S7). For empiric AAT, assessment details were missing in 23.08% (144/491 aspects).

Some of the aspects on the predefined list were infrequently or never used to define AAT: De-escalation was used as a QI in only two studies [22, 23]; diagnostic procedures [22], expert assessment [24] and switch to oral therapy [22] in one study each; documentation of therapy and process-related measures were never used. 13/62 (20.97%) studies including patients with microbiologically non-confirmed infection had a separate definition for patients without positive microbiological samples and 24 (38.71%) did not assess AAT in these patients whatsoever (supplementary Table S8). Some studies evaluated the effect of treatment-related indicators other than AAT (such as combination therapy, details in supplementary Table S9).

Of 77 studies (26.74%) reporting how their AAT definition was established, majority (68, 88.31%) adapted their definition from another publication (supplementary Table S10). Of 61 studies (21.18%) that commented on the quality of their AAT definition (supplementary Table S11), 8 (13.12%) pointed out that in-vitro-susceptibility-based approaches may fall short of determining in vivo effectiveness.

### Study characteristics and definitions of AAT

Distribution of study characteristics among the various types of definition for empiric treatment is presented in Table 3 and supplementary Table S12. Studies including patients with any infecting pathogen applied susceptibility-based definitions more often compared to those studies restricted to specific pathogens (66/116 (61.68%) vs. 39/142 (27.47%), *p* < 0.001). When bloodstream infection (BSI) was the infection of interest, studies assessed AAT more often using at least three aspects (including susceptibility) compared to studies with other infections of interest (54/166 (32.53%) vs. 10/92 (10.87%), *p* < 0.001). In definite treatment, inclusion restricted to a specific pathogen was associated with the applied definition as well (supplementary Table S13).

**Table 3:** Study characteristics and definitions of appropriate antibiotic therapy (AAT) grouped according to use of susceptibility and other aspects for empiric therapy

	Grouping of AAT definition				
	susceptibility only	Susceptibility + 1 other aspect	Susceptibility + ≥ 2 other aspects	Non-susceptibility-centered	p-value
Study characteristics	n (%)	n (%)	n (%)	n (%)	
Overall	105 (40.70%)	76 (29.46%)	64 (24.81%)	13 (5.04%)	
Study department					< 0.001
Hospital wide	55 (52.38%)	51 (67.11%)	48 (75.00%)	5 (38.46%)	
ICU	33 (31.43%)	18 (23.68%)	11 (17.19%)	1 (7.69%)	
Emergency department	6 (5.71%)	2 (2.63%)	4 (6.25%)	2 (15.38%)	
Mixed	11 (10.48%)	5 (6.58%)	-	4 (30.77%)	
Unclear	-	-	1 (1.56%)	1 (7.69%)	
Target site of acquisition in included studies					0.06
Healthcare-setting	24 (22.86%)	15 (19.74%)	11 (17.19%)	-	
Outside hospital	17 (16.19%)	11 (14.47%)	10 (15.62%)	7 (53.85%)	
Inclusion not restricted by site of acquisition	64 (60.95%)	50 (65.79%)	43 (67.19%)	6 (46.15%)	
Target pathogens in included studies					< 0.001
* P. aeruginosa*	5 (4.76%)	8 (10.53%)	7 (10.94%)	-	
* K. pneumoniae*	7 (6.67%)	8 (10.53%)	2 (3.12%)	-	
* S. aureus*	5 (4.76%)	5 (6.58%)	3 (4.69%)	2 (15.38%)	
* A. baumannii*	2 (1.90%)	4 (5.26%)	6 (9.38%)	-	
* E. coli*	1 (0.95%)	2 (2.63%)	3 (4.69%)	-	
* S. pneumoniae*	-	2 (2.63%)	1 (1.56%)	-	
Mixed specific pathogens	19 (18.10%)	23 (30.26%)	22 (34.38%)	-	
Other	-	1 (1.32%)	2 (3.12%)	2 (15.38%)	
Inclusion not restricted by infecting pathogen	66 (62.86%)	23 (30.26%)	18 (28.12%)	9 (69.23%)	
Target type of infection in included studies					0.002
Bloodstream infection	59 (56.19%)	47 (61.84%)	54 (84.38%)	6 (46.15%)	
Respiratory infection	24 (22.86%)	15 (19.74%)	3 (4.69%)	2 (15.38%)	
Urinary tract infection	7 (6.67%)	3 (3.95%)	–	–	
Abdominal infection	4 (3.81%)	2 (2.63%)	–	2 (15.38%)	
Central nervous system	1 (0.95%)	-	–	1 (7.69%)	
Skin and soft tissue	1 (0.95%)	1 (1.32%)	2 (3.12%)	–	
Mixed / Other	3 (2.86%)	4 (5.26%)	-	–	
Inclusion not restricted by type of infection	6 (5.71%)	4 (5.26%)	5 (7.81%)	2 (15.38%)	

ICU, Intensive care unit; AAT, appropriate antibiotic therapy

While studying individual aspects (supplementary Tables S14 and S15), route of administration was found to be rarely evaluated in ICU-settings, while it was relatively frequent among BSI studies for empiric AAT. 4/12 studies (25.00%) assessing guideline-based therapy were conducted in the emergency department (versus 5.43% overall, *p* = 0.002).

### Outcomes related to AAT

After excluding seven of 478 extracted outcomes due to missing data, the most frequently reported outcomes were mortality (350, 74.31%), LOS (40, 8.49%), and clinical failure (28, 5.94%). 390 (82.80%) outcomes pertained to empirical AAT and 53 to definite AAT (supplementary Tables S16 and S17). In total, 238 effect measures from 184 studies were based on multivariable analysis, finding an independent effect of AAT in 125 analyses (62.81%) for empiric and in 21 (80.77%) for definite AAT.

Ten studies investigated the effect of both empiric and definite AAT on all-cause mortality, using multivariable analyses in at least one of the analyses [25–34]. Three studies (30.00%) reported a significant effect of both empiric and definite AAT [25–27], four studies (40.00%) found a significant effect for definite AAT only [28–31], two studies (20.00%) for empiric AAT only (one significantly reducing mortality, the other significantly increasing mortality) [32, 33] and one study found no significant effect in either [34].

### Effect of varying definitions of AAT on all-cause-mortality

Further analyses explored all-cause mortality, as it accounted for 75.63% (180/238) of outcomes with multivariable analysis. Detection of a significant AAT effect on mortality was similar among all types of AAT definitions (Table 4 and supplementary Table S18), except for definition with treatment duration as an aspect, with a higher probability of significant reduction in mortality (14/14, *p* = 0.015). Of note, 13 of these 14 studies defined appropriate duration by suggesting a minimum treatment duration without indication of a maximum duration. Furthermore, no study setting or inclusion criteria impacted the effect of AAT on all-cause mortality. Interestingly, studies not finding a significant AAT effect critically appraised their AAT definition (i.e. pointing out strengths or weaknesses) more frequently than those with an effect (16/49 (32.65%) vs. 23/128 (17.97%), *p* = 0.035).

**Table 4:** Study characteristics and the measured effect of appropriate antibiotic therapy (AAT) on all-cause mortality in multivariable analysis

	Effect of AAT on all-cause mortality		
	Significant effect found^a^	Significant effect not found	
	n (%)	n (%)	*p*-value
	130 (72.63%)	49 (27.37%)	
Study characteristic			
Year of publication			0.056
2011–2014	46 (82.14%)	10 (17.86%)	
2015–2018	60 (72.29%)	23 (27.71%)	
2019–2021	24 (60.00%)	16 (40.00%)	
Study department			0.244
Hospital wide	85 (71.43%)	34 (28.57%)	
ICU	31 (79.49%)	8 (20.51%)	
Emergency department	4 (44.44%)	5 (55.56%)	
Mixed	9 (81.82%)	2 (18.18%)	
Unclear	1 (100.00%)	-	
Target site of acquisition in included studies			0.965
Healthcare-setting	26 (74.29%)	9 (25.71%)	
Outside hospital	20 (71.43%)	8 (28.57%)	
Inclusion not restricted by site of acquisition	84 (72.41%)	32 (27.59%)	
Target pathogens in included studies			0.586
* P. aeruginosa*	10 (90.91%)	1 (9.09%)	
* E. coli*	2 (66.67%)	1 (33.33%)	
* S. aureus*	6 (60.00%)	4 (40.00%)	
* K. pneumoniae*	8 (80.00%)	2 (20.00%)	
* A. baumannii*	10 (83.33%)	2 (16.67%)	
* S. pneumoniae*	2 (100.00%)	-	
Mixed specific pathogens	34 (64.15%)	19 (35.85%)	
Other	2 (66.67%)	1 (33.33%)	
Inclusion not restricted by infecting pathogen	56 (74.67%)	19 (25.33%)	
Target type of infection in included studies			0.353
Bloodstream infection	90 (70.87%)	37 (29.13%)	
Respiratory infection	16 (80.00%)	4 (20.00%)	
Urinary tract infection	1 (25.00%)	2 (75.00%)	
Abdominal infection	3 (75.00%)	1 (25.00%)	
Endocarditis	3 (100.00%)	-	
Central nervous system infection	1 (100.00%)	-	
Mixed / Other	5 (71.43%)	2 (28.57%)	
Inclusion not restricted by type of infection	11 (84.62%)	2 (15.38%)	
Source of definition specified in the paper			0.361
Yes	33 (67.35%)	16 (32.65%)	
No	95 (74.22%)	33 (25.78%)	
Reflection of quality/limitations of the definition in the paper			0.035
Yes	23 (58.97%)	16 (41.03%)	
No	105 (76.09%)	33 (23.91%)	
Definition of appropriateness			0.957
Based on susceptibility only	42 (71.19%)	17 (28.81%)	
Susceptibility + 1 aspect	23 (76.67%)	7 (23.33%)	
Susceptibility + ≥ 2 aspects	36 (73.47%)	12 (26.53%)	
Non-susceptibility-centered	7 (70.00%)	3 (30.00%)	
Aspects considered			
Susceptibility	120 (72.29%)	46 (27.71%)	0.875
Timely initiation	42 (68.85%)	19 (31.15%)	0.476
Dosing	33 (73.33%)	12 (26.67%)	0.839
Route of administration	18 (60.00%)	12 (40.00%)	0.103
Duration of treatment	14 (100.00%)	–	0.015
Guideline-based choice	10 (83.33%)	2 (16.67%)	0.371
Aminoglycoside restriction	13 (92.86%)	1 (7.14%)	0.072
Other	15 (68.18%)	7 (31.82%)	0.656

^a^Excluding data from one study in which IAT was found to significantly reduce mortality

ICU, Intensive care unit; ESBL, Extended-spectrum beta-lactamase; MRSA, Methicillin-resistant *Staphylococcus aureus*; AMS, Antimicrobial stewardship; AAT, appropriate antibiotic therapy

### Interaction of AAT with other covariates

Potential risk factors studied in multivariable analyses other than AAT are presented in supplementary Table S19. An independent AAT effect on all-cause mortality was found more often (*p* = 0.016, supplementary Table S20) among studies that considered disease severity, however not where disease severity was an accompanying independent risk factor. No significant association of study characteristics and consideration of disease severity was found.

## Discussion

We conducted a systematic assessment of AAT definitions used in clinical research. Almost all identified studies provided a definition of AAT, however, with details on the assessment of AAT frequently missing. The most frequent aspect used to define both empiric and definite AAT was in-vitro susceptibility (95% and 88%, respectively) followed by timely initiation of therapy among empiric AAT and dosing among definite AAT. Usage of aspects other than susceptibility was more common in studies focusing on specific pathogens or BSI. Empiric AAT had an independent effect on improvement of outcomes in 63% of the analyses and definite AAT in 81%, particularly for long-term and ICU mortality. However, the associations did not differ consistently by the type or complexity of AAT definition, except when minimum treatment duration was included.

### Defining AAT

Similar to our findings, previous reviews that systematically assessed definitions of AAT reported that in-vitro susceptibility was used as an aspect of AAT in 87% to 94% of applied definitions [2, 3, 6]. However, basing AAT assessment primarily on susceptibility fails to acknowledge that in vitro susceptibility does not always translate to an adequate choice of antibiotics in vivo [5, 9]. First, susceptibility-based definitions bear the risk of overlooking or even encouraging antibiotic overtreatment. A high rate of appropriateness, an apparent target in many of the studies, could potentially be achieved by unselectively prescribing broad-spectrum antibiotics or combination therapy, thereby provoking antibiotic resistance [4, 8] and negatively impacting patient outcomes [35, 36]. Only two of all included studies considered unnecessarily broad empiric therapy to be inappropriate [37, 38]. While several other studies emphasized that broad spectrum antibiotics should be reserved to selected populations only, AAT assessment was based on susceptibility only in these studies, suggesting a lack of awareness of this pitfall [8, 39–41].

At the time of selecting empiric therapy, the causative pathogen is unknown to physicians, making in-vitro appropriateness a suboptimal criterion for choice of good initial treatment. Clinical guidelines, instead, are vital in choosing an empiric therapy with the best evidence of avoiding both under- and overtreatment, based on infective focus and medical history of the patient. Nevertheless, our data did not reflect the importance of guidelines, with less than 5% of included studies considering guideline-based empirical therapy. Studies incorporating this aspect usually hypothesized that guideline adherence would be the most relevant aspect to patient outcomes at the time of initial antibiotic administration, and therefore before availability of culture results [42–44]. Two studies comparing AAT defined by either susceptibility or guidelines found that while guideline-adherence did not affect mortality, it did, however, increase the rate of in-vitro susceptibility [45, 46]. Though guideline adherence could potentially have a bigger impact on preventing emergence of antimicrobial resistance, this outcome was rarely studied.

Culture-negative infections pose another field where guidelines are of special importance to the selection of therapy, as they are the main resource for therapy selection for both empiric and definite treatment. However, we observed that most studies including both patients with and without pathogen identification either did not evaluate AAT in culture-negative patients or did not comment on this aspect.

Further, many QIs of AAT suggested by literature were rarely or never used and few studies used numerous QIs. The most exhaustive evaluation of AAT we identified was by Lee et al., where empiric therapy was evaluated using six aspects including susceptibility, timing, dosing, route of administration, duration of therapy as well as aminoglycoside use [47]. Only 3.47% (10/288) used five aspects or more [23, 48–55] and around 25% applied more comprehensive definitions integrating at least three aspects. Previous reviews had found that AAT definitions included dosing in 24% to 35%, and route of administration in 3% to 37% of studies [1–3, 6]. This is in line with our findings of 20% and 13%, respectively, indicating no change in use of these aspects over time. Timely antibiotic initiation was used in only 31% of extracted definitions, much less compared to 71% to 76% reported in previous reviews. These earlier reviews focused on sepsis or BSI, in which early antibiotic administration is of utmost importance and thus frequently used.

We found the aspects included in AAT definitions as well as the specific methods of their evaluation to vary greatly. At the same time, details of the aspects’ evaluation were insufficiently clarified in many of the studies, especially in regard to dosing and route of administration. Thus, heterogeneity of reported AAT definitions was a relevant obstacle to evidence synthesis. Making applied methods transparent is highly relevant to allow discussion in case of differing findings and for potential replication of study results. We would therefore like to point out two of the included studies which provided thorough clarifications of study methodology and can be used as a reference for good methodological reporting [56, 57].

Surprisingly, we found only few patterns in AAT definitions with regard to study characteristics. The identified associations tended to generally reflect the treatment patterns in specific settings or target patient groups. For example, guideline adherence was more commonly studied as an aspect of AAT in studies conducted in the emergency department, where empiric therapy is usually initiated. In ICU settings, route of administration was rarely studied as intravenous administration would represent the standard of care for most cases treated in intensive care.

### Effect of AAT on patient outcomes

Our findings are in line with previous research suggesting a positive effect of AAT on patient outcomes [1–4]. We found that the independent effect of AAT on all-cause mortality did not vary much according to study setting, inclusion criteria, or type of definition. The only exception was the additional use of treatment duration as a criterion of definition which was associated with a positive effect of AAT. It should however be noted that studies assessing duration are at a particularly high risk of introducing immortal time bias: In such studies, classification of antibiotic therapy as “appropriate” requires patients to survive until the timepoint considered as the minimum duration of treatment. Out of the 14 studies using duration as a definition aspect, only one study accounted for this issue by assigning patients with shorter-than-recommended therapy into the appropriate treatment group if they received antibiotics until the time of death [48]. Therefore, we believe this finding to be a result of bias rather than representing a true association.

Similar to our findings, previous reviews did not identify a clear effect of varying AAT definitions on outcome measures. A systematic review of prospective cohort studies on sepsis by Paul et al. found a higher pooled adjusted effect of IAT on mortality, though not significant (*p* = 0.095), among studies defining AAT based on susceptibility only (OR = 2.30, *n* = 24) as compared to those using additional aspects such as dosing, route and duration (OR = 1.74, *n* = 15) [1]. Though our study did not aim at quantifying the effect of AAT on all-cause mortality, hindering any direct comparison, our results partly corroborate theirs as we identified a higher percentage of positive results for in-vitro susceptibility alone (71%) as compared to route of administration (60%). Similarly, another review among patients with gram-negative infections did not identify any difference in mortality rates when AAT was defined based on susceptibility alone or both susceptibility- and timeliness-based [3].

Of note, as the methods used to measure appropriateness varied considerably between studies, there was substantial heterogeneity even within studies with similar definition aspects. We therefore repeated analyses after restriction to studies on BSI or ICU setting only. Within these subgroups, we found some individual definition aspects of AAT to impact outcome; however, this did not apply to the main definition categories.

Moreover, a matter of concern was the lack of multivariable analysis in one third of the studies, hampering their contribution to the evidence base. Comprehensive statistical methods taking into consideration multiple covariates at various levels and varying effects as well as their intra- and inter-correlations should be used instead. Based on our findings, we have summarized some recommendations for future research examining the effect of AAT (Table 5).

**Table 5:** Recommendations for future research examining the effect of AAT on patient outcomes

Recommendation 1	We recommend that the assessment of both empiric and definite antibiotic therapy consistently includes adherence to relevant clinical treatment guidelines. Although justified deviations may be necessary in specific clinical situations, guidelines provide the most robust evidence base for selecting agents with appropriate spectrum and focus-specific activity. Incorporating guideline adherence also allows appropriateness to be evaluated in patients without microbiological confirmation, where susceptibility testing is not available to inform treatment decisions.
Recommendation 2	Additional aspects relevant to the evaluation of empiric AAT, when not already captured by guideline adherence, should include the timeframe for initiation of antibiotics according to diagnosis and disease severity, dosing as well as diagnostic procedures such as taking blood cultures before starting antibiotic therapy. In studies that choose to apply susceptibility-centred definitions for empiric and/or definitive therapy, we recommend explicitly incorporating criteria that identify unnecessary use of broad-spectrum agents as inappropriate, to avoid misclassifying overtreatment as appropriate care.
Recommendation 3	The evaluation of definite therapy should be assessed separately from the assessment of empiric therapy and should be guided by established aspects. We suggest that these should at least include: de-escalation or streamlining based on culture results, route of administration with timely switch to oral therapy when feasible, correct dosing of antibiotics, and appropriate duration of treatment.
Recommendation 4	The impact of adherence to each individual component of AAT, as well as adherence to a comprehensive set of components collectively, should be systematically evaluated in relation to patient outcomes.
Recommendation 5	Whenever feasible, the emergence of antimicrobial resistance should be included as a key outcome measure.
Recommendation 6	When analyzing the effect of AAT on patient outcomes appropriate adjustment for relevant covariates should be considered. Disease severity and comorbidities should be measured in all studies, using standardized scoring systems, and variables such as immunosuppression status, infection with resistant pathogens, and other clinically relevant factors should be considered based on study context. The effect of AAT on patient outcomes should be evaluated using multivariable analysis or other analytical methods such as propensity score methods, to adjust for confounding. Results of both univariable and multivariable analyses should be fully provided, if applicable.
Recommendation 7	Study methods should be made fully transparent in regard to both the assessment of AAT and outcome analysis. The criteria and procedures used to evaluate AAT should be provided in full detail and any complex assessment algorithms should be made available, for example as supplementary material. When definitions of AAT are adopted from previous work, the original source should be referenced.

AAT, appropriate antibiotic therapy

### Limitations

Our study has some limitations. First, the comprehensive nature of the review and the wide range of study designs and settings in which AAT is assessed led to a very heterogenous group of included studies with limited comparability, which could not be averted. Second, our analysis included studies published from 2011 to 2021, a period in which we could safely assume the awareness of AMS to be fairly widespread. We could not extend the data to later years owing to logistic and time constraints; however, we did not see a major shift in the patterns of AAT definitions in a sample of 17 studies assessing the effect of AAT on patient outcomes published between 2023 and 2025. The appraised aspects corresponded to the presented data, with susceptibility being used in all studies (100.00%), timing in seven (41.18%) and dosing and route of administration in two studies each (11.76%) [58–74]. Third, most of the included studies did not state the study of AAT effect on patient outcomes as an objective. Hence, it is likely that our search identified a considerable number of studies that incidentally found a significant effect of AAT, while those not finding such an effect refrained from reporting it in their abstract and were not included. Moreover, many studies did not report all variables included in multivariable analysis but only the significant ones. Almost a fourth of the included studies with a significant crude AAT effect (17/76 studies) reported a multivariable analysis but not providing further data regarding the AAT effect. Both of these could have overestimated the frequency of a significant effect; nonetheless, we do not expect the overestimation to vary significantly across AAT definitions or study characteristics. Fourth, relevant studies could have been missed as inclusion was restricted to studies published in English. Fifth, as in any scoping review, formal risk-of-bias assessment was not performed, and therefore, the presented associations of AAT definitions and measured outcomes should be interpreted cautiously.

## Conclusion

Definitions of AAT applied in clinical studies continue to rely almost exclusively on in-vitro susceptibility, with limited integration of AMS-oriented criteria such as guideline adherence, dosing, de-escalation, and treatment duration. In addition, details on evaluation of many aspects were frequently not reported, particularly for dosing and route of administration. While AAT generally correlates with improved outcomes, methodological heterogeneity and insufficient adjustment for confounders limit comparability across studies. Future research should adopt standardized, multidimensional AAT definitions and consistently adjust for disease severity and comorbidities. Incorporating stewardship-based QIs and routinely evaluating effects on antimicrobial resistance will strengthen the clinical and public health relevance of AAT research.

## Supplementary Information

Below is the link to the electronic supplementary material.

Supplementary file1 (PDF 1022 KB)

## Data Availability

The data were extracted from published articles, and these data, analyzed during the current study, are available from the corresponding author upon reasonable request.

## Declarations

### Standards of reporting

PRISMA checklist in the appendix.
