# Supplementary material for: Assessing appropriateness of antibiotic therapy: a scoping review of definitions and their clinical implication
Source: Infection. 2026 Jan 31;54(3):1077–91. doi: 10.1007/s15010-026-02733-x (PMC13323112; doi:10.1007/s15010-026-02733-x)
Supplement: Supplementary file 1 — Supplementary file1 (PDF 1022 KB) [file 15010_2026_2733_MOESM1_ESM.pdf]

Supplementary Information for

Assessing Appropriateness of Antibiotic Therapy: A Scoping Review of Definitions and Their Clinical Implication

Lea A. Nikolai, Beryl P. Gladstone, Arisa Hakariya, Marissa Rink, Evelina Tacconelli, Siri Göpel

Corresponding author:

Dr. med. Siri Göpel

Division of Infectious Diseases, Department of Internal Medicine I, University Hospital Tübingen

Table of Contents

List of abbreviations 3

S Table 1: Preferred Reporting Items for Systematic reviews and Meta-Analyses extension for Scoping Reviews (PRISMA-ScR) Checklist 4

S Section 1: Detailed Study Methodology 6

S Section 2: Search Terms used in PubMed, Web of Science and Cochrane Central Register of Controlled Trials (CENTRAL) 9

S Section 2: Grouping of study outcomes: clinical failure, clinical success and adverse events 11

S Table 2: Overview of characteristics of included studies 12

S Table 3: Overview of appropriate antibiotic therapy (AAT) definitions by study and respective aspects of AAT evaluated 43

S Table 4: Definitions of the terms "empiric" and "definite" therapy reported in the studies 56

S Table 5: Overview of aspects included in study definitions of appropriate antibiotic therapy (AAT) for unclear and other treatment phase 57

S Table 6: Detailed methods for evaluation of appropriate antibiotic therapy (AAT) aspects in study definitions of empiric therapy (n=258) 58

S Table 7: Detailed methods for evaluation of appropriate antibiotic therapy (AAT) aspects in study definitions of definite therapy (n=41) 60

S Table 8: Assessment of appropriate antibiotic therapy (AAT) in patients without pathogen identification among studies including such patients and providing information on AAT assessment 61

S Table 9: Assessment of AMS objectives and individual appropriate antibiotic therapy (AAT) aspects for their effect on patient outcomes 62

S Table 10: Sources and other ways of determining study definitions of appropriate antibiotic therapy (AAT) 63
S Table 11: Mentioned strengths and limitations of appropriate antibiotic therapy (AAT) definitions among studies providing critical assessment of their respective study definitions 64
S Table 12: Study characteristics and definitions of appropriate antibiotic therapy (AAT) grouped according to use of susceptibility and other aspects for empiric therapy (n=258) 65
S Table 13: Study characteristics and definitions of appropriate antibiotic therapy (AAT) grouped according to use of susceptibility and other aspects for definite therapy (n=41) 67
S Table 14: Study characteristics and definitions of appropriate antibiotic therapy (AAT) grouped according to use of individual aspects for empiric therapy (n=258) 69
S Table 15: Study characteristics and definitions of appropriate antibiotic therapy (AAT) grouped according to use of individual aspects for definite therapy (n=41) 72
S Table 16: Overview of outcome measures and effect of empiric appropriate antibiotic therapy (AAT) on patient outcomes in univariate and multivariate analyses reported by the studies 75
S Table 17: Overview of outcome measures and effect of definite appropriate antibiotic therapy (AAT) on patient outcomes in univariate and multivariate analyses reported by the studies 76
S Table 18: Study characteristics and the measured effect of empiric appropriate antibiotic therapy (AAT) on all-cause mortality in the multivariable analyses reported by the studies 77
S Table 19: Covariates considered as potential risk factors for unfavorable outcome among studies reporting multivariable analysis 79
S Table 20: Relationship between the considered risk factors for mortality and the measured effect of appropriate antibiotic therapy (AAT) on all-cause mortality in multivariable analysis reported in the studies[a] 81
References 83

List of abbreviations

AAT	appropriate antibiotic therapy
AMS	antimicrobial stewardship
CDI	*Clostridioides difficile* infection
CNS	central nervous system
ED	emergency department
ESBL	extended-spectrum beta-lactamase
ICU	intensive care unit
i.v.	intravenous
IQR	interquartile range
KPC	*Klebsiella pneumoniae* carbapenemase
LOS	length of stay
MDR	multi drug resistance
PRISMA-ScR	Preferred Reporting Items for Systematic reviews and Meta-Analyses extension for Scoping Reviews
RCT	randomized controlled trial
spp.	species pluralis
A. baumannii	*Acinetobacter baumannii*
A. calcoaceticus	*Acinetobacter calcoaceticus*
A. nosocomialis	*Acinetobacter nosocomialis*
C. difficile	*Clostridioides difficile*
E. aerogenes	*Enterobacter aerogenes*
E. cloacae	*Enterobacter cloacae*
E. coli	*Escherichia coli*
E. faecalis	*Enterococcus faecalis*
E. faecium	*Enterococcus faecium*
E. meningoseptica	*Elizabethkingia meningoseptica*
K. oxytoca	*Klebsiella oxytoca*
K. pneumoniae	*Klebsiella pneumoniae*
L. monocytogenes	*Listeria monocytogenes*
L. pneumophila	*Legionella pneumophila*
MRSA	Methicillin-resistant *Staphylococcus aureus*
MSSA	Methicillin-sensitive *Staphylococcus aureus*
P. aeruginosa	*Pseudomonas aeruginosa*
P. mirabilis	*Proteus mirabilis*
S. agalactiae	*Streptococcus agalactiae*
S. aureus	*Staphylococcus aureus*
S. maltophilia	*Stenotrophomonas maltophilia*
S. pneumoniae	*Streptococcus pneumoniae*
S. saprophyticus	*Staphylococcus saprophyticus*
VRE	Vancomycin-resistant *enterococci*

S Table 1: Preferred Reporting Items for Systematic reviews and Meta-Analyses extension for Scoping Reviews (PRISMA-ScR) Checklist

SECTION	ITEM	PRISMA-ScR CHECKLIST ITEM	REPORTED ON PAGE #
**TITLE**			
Title	1	Identify the report as a scoping review.	p. 1
**ABSTRACT**			
Structured summary	2	Provide a structured summary that includes (as applicable): background, objectives, eligibility criteria, sources of evidence, charting methods, results, and conclusions that relate to the review questions and objectives.	p. 2
**INTRODUCTION**			
Rationale	3	Describe the rationale for the review in the context of what is already known. Explain why the review questions/objectives lend themselves to a scoping review approach.	p. 3
Objectives	4	Provide an explicit statement of the questions and objectives being addressed with reference to their key elements (e.g., population or participants, concepts, and context) or other relevant key elements used to conceptualize the review questions and/or objectives.	p. 3
**METHODS**			
Protocol and registration	5	Indicate whether a review protocol exists; state if and where it can be accessed (e.g., a Web address); and if available, provide registration information, including the registration number.	p. 4
Eligibility criteria	6	Specify characteristics of the sources of evidence used as eligibility criteria (e.g., years considered, language, and publication status), and provide a rationale.	p. 4
Information sources*	7	Describe all information sources in the search (e.g., databases with dates of coverage and contact with authors to identify additional sources), as well as the date the most recent search was executed.	p. 4
Search	8	Present the full electronic search strategy for at least 1 database, including any limits used, such that it could be repeated.	Suppl. p. 9-10
Selection of sources of evidence†	9	State the process for selecting sources of evidence (i.e., screening and eligibility) included in the scoping review.	p. 4
Data charting process‡	10	Describe the methods of charting data from the included sources of evidence (e.g., calibrated forms or forms that have been tested by the team before their use, and whether data charting was done independently or in duplicate) and any processes for obtaining and confirming data from investigators.	p. 4
Data items	11	List and define all variables for which data were sought and any assumptions and simplifications made.	p. 4-5, Suppl. p. 7-8
Critical appraisal of individual sources of evidence§	12	If done, provide a rationale for conducting a critical appraisal of included sources of evidence; describe the methods used and how this information was used in any data synthesis (if appropriate).	not applicable
Synthesis of results	13	Describe the methods of handling and summarizing the data that were charted.	p. 5
**RESULTS**			

SECTION	ITEM	PRISMA-ScR CHECKLIST ITEM	REPORTED ON PAGE #
Selection of sources of evidence	14	Give numbers of sources of evidence screened, assessed for eligibility, and included in the review, with reasons for exclusions at each stage, ideally using a flow diagram.	p. 6
Characteristics of sources of evidence	15	For each source of evidence, present characteristics for which data were charted and provide the citations.	Suppl. p. 12-55
Critical appraisal within sources of evidence	16	If done, present data on critical appraisal of included sources of evidence (see item 12).	not applicable
Results of individual sources of evidence	17	For each included source of evidence, present the relevant data that were charted that relate to the review questions and objectives.	Suppl. p. 12-55
Synthesis of results	18	Summarize and/or present the charting results as they relate to the review questions and objectives.	p. 5-13
**DISCUSSION**			
Summary of evidence	19	Summarize the main results (including an overview of concepts, themes, and types of evidence available), link to the review questions and objectives, and consider the relevance to key groups.	p. 13-14
Limitations	20	Discuss the limitations of the scoping review process.	p. 18
Conclusions	21	Provide a general interpretation of the results with respect to the review questions and objectives, as well as potential implications and/or next steps.	p. 18
**FUNDING**			
Funding	22	Describe sources of funding for the included sources of evidence, as well as sources of funding for the scoping review. Describe the role of the funders of the scoping review.	p. 19

JBI = Joanna Briggs Institute; PRISMA-ScR = Preferred Reporting Items for Systematic reviews and Meta-Analyses extension for Scoping Reviews.

* Where *sources of evidence* (see second footnote) are compiled from, such as bibliographic databases, social media platforms, and Web sites.

† A more inclusive/heterogeneous term used to account for the different types of evidence or data sources (e.g., quantitative and/or qualitative research, expert opinion, and policy documents) that may be eligible in a scoping review as opposed to only studies. This is not to be confused with *information sources* (see first footnote).

‡ The frameworks by Arksey and O'Malley (6) and Levac and colleagues (7) and the JBI guidance (4, 5) refer to the process of data extraction in a scoping review as data charting.

§ The process of systematically examining research evidence to assess its validity, results, and relevance before using it to inform a decision. This term is used for items 12 and 19 instead of "risk of bias" (which is more applicable to systematic reviews of interventions) to include and acknowledge the various sources of evidence that may be used in a scoping review (e.g., quantitative and/or qualitative research, expert opinion, and policy document).

From: Tricco AC, Lillie E, Zarin W, O'Brien KK, Colquhoun H, Levac D, et al. PRISMA Extension for Scoping Reviews (PRISMAScR): Checklist and Explanation. Ann Intern Med. 2018;169:467–473. doi: 10.7326/M18-0850.

S Section 1: Detailed Study Methodology

In order to gain a comprehensive overview of studies and identify existing research gaps, a systematic literature search was conducted. We targeted studies evaluating the effect of AAT on patient outcomes in adult patients with confirmed or suspected bacterial infections treated in the inpatient setting. Studies published after 2011 were included as we did not expect earlier publications to incorporate AMS recommendations on defining AAT.

Search Strategy

We conducted the literature search on November 24, 2021 in PubMed, Web of Science and Cochrane Library. Search terms combining antibiotic therapy, appropriate or adequate and outcome measures were used (full search terms provided in supplements p. 9. Originally, search terms were developed to identify studies evaluating the impact of antibiotic stewardship programs on AAT as well. However, this group of studies is not included in the current analysis and will be presented elsewhere, as we focus here on research associating risk factors with clinical outcomes. Search results were managed with EndNote.

Eligibility Criteria

Screening procedures were performed by one of the authors (LN) and questions were discussed with a second author (PB). Discrepancies were sorted and reason for exclusion was noted. No blinding to study authors or location was done. Retrospective and prospective observational studies with control groups were included, but not reviews, case reports or conference contributions. Inclusion was limited to studies available in English language. Exclusion criteria were: studies evaluating prophylactic antibiotic use, studies conducted in dental care or outpatient settings, nursing homes and long-term care facilities, pediatric cohorts, cohorts with over 10% percent of patients infected with a non-bacterial pathogen, cohorts focusing on COVID-19 or HIV only. Publications had to evaluate the impact of appropriate or adequate antibiotic therapy on patient outcomes and/or emergence of antibiotic resistance. When multiple publications presented data from the same study or database and analyzed time periods were overlapping, we chose to include the study presenting a larger cohort of patients and/or the more recent publication only.

Data Extraction

The data was extracted by two of the authors (LN and AH), and questions were discussed with a third author (PB). Extracted variables included study inclusion criteria, the definition(s) of AAT applied and all available outcomes of interest. Variables were extracted into a REDCap database.

Definition of AAT

Recent literature suggesting QIs for AAT was used to create a predefined list of potential aspects used in definitions of AAT. Each study definition of AAT was checked for the following aspects: in-vitro susceptibility, timely initiation of therapy, dosing, route of administration, aminoglycoside restriction, duration of therapy, guideline-based choice of therapy, expert assessment, diagnostic procedures (such as taking blood cultures), adequate documentation of therapy in patient notes, antibiotic de-escalation, switch to oral therapy when feasible and process-related measures (such as bedside consultation). When other aspects were used, these were additionally extracted. The detailed steps used to determine the appropriateness of treatment based on these aspects were investigated. When a study used a comprehensive definition for AAT (for overall treatment) but reported outcomes in relation to empirical and definite therapy separately, we counted the definition towards both empirical and definite AAT individually. Otherwise, it was considered a definition for the entire treatment course. Definitions of AAT that had no related outcome were excluded from data analysis, while outcomes without an associated definition were considered to pertain to an "unclear" definition.

To investigate factors that influenced how AAT was defined, we grouped AAT definitions as follows: studies defining appropriateness by susceptibility only, by susceptibility and one other aspect (with a subcategory of susceptibility and timely initiation of therapy), by susceptibility and at least two other aspects and studies defining AAT non-susceptibility centered (such as guideline-based definitions). In this context, susceptibility referred to therapy based on individual in-vitro susceptibility testing. We also studied the development of the AAT definition, and the limitations and/or strengths mentioned by study authors regarding their own definitions. We compared relevant characteristics of studies including type of infection setting after grouping similar AAT definitions to identify any pattern in the use of definitions.

Outcomes related to AAT

All patient outcomes reported in relation to AAT were included. Details on the direction of association (AAT or IAT associated with favorable outcome), type of analysis (univariable or multivariable), and whether a significant effect ($p < 0.05$) was found, were extracted. Results of multivariable analysis were extracted whenever available; when not, univariable analysis was used. An association was referred to as independent when AAT was significantly and favorably associated with the outcome after adjustment for other risk factors in a multivariable analysis, e.g. AAT being significantly associated with lower mortality or shorter LOS. Outcomes based on the entire study cohort were preferred over subgroup-based outcomes. All-cause mortality was classified as early mortality (2 to 15 days), late mortality (21 to 30 days) or long-term mortality (>30 days to 1-year mortality). Overall mortality

without mention of a time period was grouped along with in-hospital mortality. Clinical failure was summarized along with clinical success after harmonizing the direction of association (details provided in supplements p. 11).

Potential risk factors

We also evaluated which potential risk factors other than AAT were considered for their effect on outcomes of interest. Covariates related to disease severity, comorbidities, immunosuppression and infection with a resistant pathogen were specifically checked to see whether they were considered as a risk factor in the study (as univariable analysis). All independent risk factors found significant upon multivariable analysis were broadly classified into categories of disease severity, comorbidities, immunosuppression, infection with a resistant pathogen, age, treatment, past treatment, pathogen, focus of infection, site of acquisition, institutional factors, sex, and microbiological factors.

Other definitions

Sepsis, septic shock, febrile neutropenia and bacteremia (including bacteremia due to specific foci, such as bacteremic urinary tract infection) were summarized as bloodstream infection (BSI). A subgroup of severe BSI including sepsis and septic shock was formed. With regard to acquisition, studies were classified as enrolling healthcare-acquired infection (HCAI, including both hospital-acquired and healthcare-associated infection), community-acquired infection (CAI, infections acquired outside the hospital as well as studies combining CAI and healthcare-associated infection) or unrestricted. Studies targeting patients with malignancy, undergoing stem cell transplantation or solid organ transplant recipients were grouped together as “immunosuppressed and cancer patients”.

S Section 2: Search Terms used in PubMed, Web of Science and Cochrane Central Register of Controlled Trials (CENTRAL)

PubMed:

("Anti-Bacterial Agents"[Mesh] OR "Anti-Bacterial Agents" [Pharmacological Action] OR Anti-infective agents [MeSH] OR Antibiotic* [tiab] OR Antiinfective* [tiab] OR "Anti infective*" [tiab] OR Antimicrobial* [tiab] OR "Anti microbial*" [tiab] OR Antibacterial* [tiab] OR "Anti bacterial*" [tiab]) AND (Stewardship [tiab] OR Policy [tiab] OR Policies [tiab] OR guideline*[tiab] OR inappropriat*[tiab] OR inadequat*[tiab] OR appropriat*[tiab] OR adequate*[tiab]) AND ((mortalit*[tiab] OR death[tiab] OR dead[tiab] OR alive[tiab] OR surviv*[tiab] OR "length of stay"[tiab] OR (("hospital stay"[tiab] OR hospitalization[tiab]) AND (length[tiab] OR period[tiab])) OR "length of hospitalisation"[tiab] OR "duration of hospitalization"[tiab] OR LOS[tiab] OR "Morbidity"[Mesh:NoExp] OR morbidit*[tiab] OR "Drug Resistance"[Mesh] OR resistan*[tiab])) AND ("2011/01/01"[Date - Publication] : "2021/11/24"[Date - Publication]) AND ("observational study"[tiab] OR "retrospective study"[tiab] OR "prospective study"[tiab] OR "trial"[tiab] OR "cohort"[tiab] OR "intervention* study"[tiab] OR "case-control study"[tiab] OR "case control study"[tiab] OR "cross sectional study"[tiab] OR "cross-sectional study"[tiab] OR "prospective analysis"[tiab] OR "retrospective analysis"[tiab] OR "study investigation"[tiab] OR "study investigati"[tiab] OR research[tiab] OR intervention*[tiab]) NOT "review"[Title] NOT "Malaria*"[tiab] NOT "HIV"[tiab] NOT (Review[Publication Type]) NOT (systematic review[Publication Type]) NOT ("dental" [tiab])) NOT ("dentist*" [tiab])) NOT ("antifung*" [Title])) NOT ("neonat*" [Title])) NOT ("infant*" [Title])) NOT children[Title])) NOT pediatric[Title])) NOT paediatric[Title])) NOT ("ambulatory" [Title])) NOT ("primary care" [Title])) NOT ("outpatient*" [Title])) NOT ("general practitioner*" [Title])) NOT ("prophyla*" [Title]) NOT ("COVID" [Title]) NOT (Case reports[pt]) NOT (editorial[pt]) NOT (News[pt]) AND (humans[Filter]))

Web of Science:

TS=("Anti bacterial" OR "anti infective" OR antibiotic OR antiinfective OR antimicrobial OR "anti microbial" OR antibacterial) AND TS=(stewardship OR (polic* NEAR/3 antibiotic) OR (polic* NEAR/3 antimicrobial) OR (guideline* NEAR/3 antibiotic) OR (guideline* NEAR/3 antimicrobial) OR inappropriat* OR inadequat* OR appropriat* OR adequat*) AND TS=("mortalit*" OR "death" OR "dead" OR "alive" OR "survi*" OR "length of stay" OR "length of hospitalisation" OR "duration of hospitalization" OR "LOS" OR "morbidit*" OR "resistan*") AND TS= ("observational study" OR "retrospective NEAR/3 study" OR "prospective NEAR/3 study" OR "trial" OR "cohort" OR "intervention* NEAR/5 study" OR "case-control* study" OR "case control* NEAR/3 study" OR "prospective NEAR/5 analys*s" OR "retrospective NEAR/5 analys*s" OR "research NEAR/5 investigati*" OR "study NEAR/5 investigati*" OR research OR cross-sectional NEAR/3 study OR "cross sectional" NEAR/3 study) NOT TS=("malaria*" OR "HIV" OR "dental" OR "dentist*") NOT TI=("neonat*" OR "pediatric*" OR "ambulatory" OR "primary care" OR "outpatient*" OR "general practitioner*" OR "prophyla*" OR "COVID" OR "review") NOT DT==("REVIEW" OR "RETRACTION" OR "REPRINT" OR "CORRECTION" OR "MEETING ABSTRACT" OR "LETTER" OR "BOOK CHAPTER" OR "EDITORIAL MATERIAL" OR "PROCEEDINGS PAPER") NOT TS=("veterinary" OR "animal" OR "agriculture")

Cochrane Central Register of Controlled Trials (CENTRAL):

#1	("Anti bacterial" OR "anti infective" OR antibiotic OR antiinfective OR antimicrobial OR "anti microbial" OR antibacterial):ti,ab,kw
**#2**	("mortalit*" OR "death" OR "dead" OR "alive" OR "survi*" OR "length of stay" OR "length of hospitalisation" OR "duration of hospitalization" OR "LOS" OR "morbidit*" OR "resistan*"):ti,ab,kw
**#3**	(stewardship OR (polic* NEAR antibiotic) OR (polic* NEAR antimicrobial) OR (guideline* NEAR antibiotic) OR (guideline* NEAR antimicrobial) OR inappropriat* OR inadequat* OR appropriat* OR adequat*):ti,ab,kw
**#4**	("observational study" OR (retrospective NEAR study) OR (prospective NEAR study) OR "trial" OR "cohort" OR (intervention* NEAR study) OR "case-control*study" OR ("case control" NEAR study) OR (prospective NEAR analys*s) OR (retrospective NEAR analys*s) OR (research NEAR investigati*) OR (study NEAR investigati*) OR "research" OR (cross-sectional NEAR study) OR ("cross sectional" NEAR study)):ti,ab,kw
**#5**	#1 AND #2 AND #3 AND #4
**#6**	("neonat*" OR "pediatric*" OR "ambulatory" OR "primary care" OR "outpatient*" OR "general practicioner*" OR "prophylax*" OR "COVID" OR "review" OR "veterinary" OR "animal" OR "agriculture"):ti
**#7**	("malaria*" OR "HIV" OR "dental" OR "dentist*"):ti,ab,kw
**#8**	#5 NOT (#6 OR #7) with Cochrane Library publication date Between Jan 2011 and Nov 2021

S Section 2: Grouping of study outcomes: clinical failure, clinical success and adverse events

Outcomes grouped together as “clinical failure”:

- Readmission or death within 30 days
- Recurrence of urinary tract infection
- Poor prognosis
- Progression from tracheobronchitis to pneumonia
- Slowly resolving pneumonia
- Unfavorable outcomes

Outcomes grouped together as “clinical success”:

- Resolution of pneumonia on day 28
- Time until defervescence and time until normalization of peripheral leucocyte count (composite)
- Resolution of secondary peritonitis
- Clinical response within 72 hours (decline of fever, leukocytosis and C-reactive protein, improvement of hypoxemia and shock)
- Extubation or discharge alive
- Discharge alive after more than 48h without transition to hospice or palliative care

Outcomes grouped as “adverse events”:

- Infectious complications
- Mechanical ventilation
- Surgical complications by day 30
- Relaparotomy or percutaneous drainage by day 30

S Table 2: Overview of characteristics of included studies

First Author	Year	Study design[a]	Country	Mention of AAT in study objectives	Pathogen	Infectious Focus	Phase(s) of antibiotic treatment studied	Outcome
De Rosa [1]	2011	retrospective cohort study	Italy	yes	ESBL-producing *E. coli*, *K. pneumoniae* or *P. mirabilis*	bacteremia	empiric	all-cause 21-day mortality
Fayad [2]	2011	retrospective cohort study	France	no	unrestricted	infective endocarditis	unclear	all-cause in-hospital mortality
Fernández-Hidalgo [3]	2011	prospective cohort study	Spain	yes	unrestricted	left-sided infective endocarditis	overall	- all-cause in-hospital mortality<br>- adverse events (acute kidney injury)
Johnson [4]	2011	retrospective cohort study	United States	no	gram-negative bacteria	severe sepsis	empiric	all-cause in-hospital mortality
Joo [5]	2011	retrospective cohort study	South Korea	yes	*P. aeruginosa*	bacteremia	empiric	all-cause 30-day/1 month mortality
Lin [6]	2011	retrospective case cohort based on case control study	Taiwan	no	unrestricted	bacteremia	empiric	all-cause 28-day mortality
Micek [7]	2011	retrospective cohort study	United States	yes	unrestricted	healthcare-associated pneumonia	empiric	all-cause in-hospital mortality
Montravers [8]	2011	prospective cohort study	France	no	unrestricted	unrestricted	empiric	all-cause ICU mortality
Plataki [9]	2011	retrospective / secondary analysis of prospective data	United States	no	unrestricted	septic shock	empiric	adverse events (acute kidney injury)
Reisfeld [10]	2011	retrospective cohort study	Israel	yes	gram-negative bacteria	bacteremia	- empiric<br>- definite	all-cause 30-day/1 month mortality
Rello [11]	2011	prospective cohort study	Belgium, France, Germany,	no	unrestricted	hospital-acquired pneumonia, ventilator-	empiric	- all-cause mortality<br>- ICU length of stay

			Greece, Italy, Ireland, Portugal, Spain, Turkey			associated pneumonia		- duration of mechanical ventilation
Schechner [12]	2011	prospective cohort study	Israel	yes	*P. aeruginosa*	bacteremia	empiric	all-cause in-hospital mortality
Schreiber [13]	2011	retrospective cohort study	United States	no	*S. aureus*	pneumonia	empiric	all-cause in-hospital mortality
Seligman [14]	2011	prospective cohort study	Brazil	no	unrestricted	ventilator-associated pneumonia	empiric	all-cause 28-day mortality
Shorr [15]	2011	retrospective cohort study	United States	yes	gram-negative bacteria	severe sepsis	empiric	in-hospital length of stay
Suppli [16]	2011	retrospective / secondary analysis of prospective data	Denmark	yes	*E. faecalis*, *E. faecium*	bloodstream infection	overall	all-cause 30-day/1 month mortality
Tumbarello [17]	2011	retrospective cohort study	Italy	no	*P. aeruginosa*	bloodstream infection	empiric	all-cause 21-day mortality
Wang [18]	2011	retrospective cohort study	Taiwan	no	ESBL-producing *E. coli* or *K. pneumoniae*	bacteremia	- empiric<br>- definite	all-cause 14-day mortality
Zarkotou [19]	2011	prospective cohort study	Greece	yes	KPC-producing *K. pneumoniae*	bloodstream infection	- empiric<br>- definite	infection-related in-hopital mortality
Aguilar-Duran [20]	2012	prospective cohort study	Spain	no	gram-negative bacilli, *Enterococcus* spp, *Staphylococcus* spp, betahemolytic *Streptococcus* spp.	bacteremic urinary tract infection	empiric	all-cause 30-day/1 month mortality
Ariza [21]	2012	retrospective cohort study	Spain	yes	unrestricted	spontaneous bacterial peritonitis	empiric	all-cause 30-day/1 month mortality
Bouza [22]	2012	prospective cohort study	Spain	no	unrestricted	ventilator-associated pneumonia	empiric	all-cause in-hospital mortality

Castillo [23]	2012	retrospective case cohort based on case control study	Colombia	no	*S. aureus*	bacteremia	empiric	all-cause 30-day/1 month mortality
Chidiac [24]	2012	prospective cohort study	France	no	*L. pneumophila*	Legionnaires' disease	- empiric<br>- definite	all-cause 30-day/1 month mortality
Chuang [25]	2012	prospective cohort study	Taiwan	yes	*A. baumannii*	bacteremia	unclear	microbiological failure
de Gouvêa [26]	2012	retrospective cohort study	Brazil	no	*A. baumannii*	unrestricted	empiric	- infection-related mortality<br>- all-cause 30-day/1 month mortality
Gözel [27]	2012	prospective cohort study	Turkey	no	gram-negative bacteria	bloodstream infection	empiric	- all-cause 14-day mortality<br>- all-cause 30-day/1 month mortality
Halilovic [28]	2012	retrospective cohort study	United States	no	unrestricted	cellulitis, cutaneous abscess	empiric	clinical failure (repeat incision and drainage, change in antimicrobial therapy or extension of antimicrobial duration due to inadequate response)
Hernández-Torres [29]	2012	prospective cohort study	Spain	yes	multidrug and carbapenem-resistant *A. baumannii*	unrestricted	- empiric<br>- definite	all-cause in-hospital mortality
Horino [30]	2012	retrospective cohort study	Japan	no	*P. aeruginosa*	bacteremia	empiric	all-cause 30-day/1 month mortality
Huang [31]	2012	retrospective cohort study	Taiwan	no	*A. baumannii*	bacteremia	empiric	all-cause 30-day/1 month mortality
Jung [32]	2012	retrospective cohort study	South Korea	no	*K. pneumoniae*	bloodstream infection	- empiric<br>- definite	all-cause 30-day/1 month mortality
Kim [33]	2012	retrospective cohort study	South Korea	yes	*A. baumannii*	bloodstream infection	definite	all-cause 14-day mortality

Lee [34]	2012	retrospective cohort study	Taiwan	no	ESBL-producing *E. coli* or *K. pneumoniae*	bacteremia	empiric	infection-related in-hopital mortality
Lye [35]	2012	retrospective cohort study	Singapore	no	Enterobacteriaceae, *P. aeruginosa*, *A. baumannii*	septic bacteremia	- empiric<br>- definite	- all-cause 30-day/1 month mortality<br>- post-infection hospital length of stay
Micek [36]	2012	retrospective cohort study	United States	no	gram-negative bacteria	severe sepsis or septic shock	empiric	in-hospital length of stay
O'Neal [37]	2012	retrospective cohort study	United States	no	*Enterobacter* spp.	bacteremia	empiric	- all-cause in-hospital mortality<br>- clinical failure (non-resolving infection signs, ongoing fluid resuscitation or persistent Enterobacter bacteremia within 72 h, inotropic support > 72 h)
Park [38]	2012	retrospective cohort study	South Korea	no	*P. aeruginosa*	bacteremic pneumonia	empiric	- all-cause 7-day mortality<br>- all-cause 14-day mortality<br>- all-cause 28-day mortality
Qureshi [39]	2012	retrospective case cohort based on case control study	United States	no	*E. coli*, *K. pneumoniae*, *K. oxytoca*, *E. cloacae*, *E. aerogenes*	bacteremia	empiric	all-cause 28-day mortality
Sancho [40]	2012	prospective cohort study	Spain	no	unrestricted	bloodstream infection	empiric	infection-related in-hopital mortality
Silveira [41]	2012	retrospective cohort study	Brazil	yes	unrestricted	community-acquired pneumonia	empiric	all-cause 30-day/1 month mortality

Tabah [42]	2012	prospective cohort study	Belgium, Netherlands, Germany, Poland, Romania, Hungary, Turkey, Greece, Serbia, Croatia, Italy, Austria, Spain, Portugal, France, Switzerland, Canada, Brazil, Morocco, Tunisia, United Arab Emirates, China, Australia, Japan	no	unrestricted	bloodstream infection	unclear	all-cause 28-day mortality
Tumbarello [43]	2012	retrospective case cohort based on case control study	Italy	no	*P. mirabilis*	bloodstream infection	empiric	all-cause 21-day mortality
Wu [44]	2012	prospective cohort study	Taiwan	no	ESBL-producing *E. coli*	bloodstream infection	empiric	all-cause 30-day/1 month mortality
Zervos [45]	2012	retrospective cohort study	United States	yes	unrestricted	complicated skin and soft tissue infection	empiric	- all-cause in-hospital mortality<br>- in-hospital length of stay<br>- clinical failure (readmission or death by day 30)

Adrie [46]	2013	retrospective / secondary analysis of prospective data	France	yes	unrestricted	community-acquired pneumonia	empiric	all-cause 60-day/2 month mortality
Bowers [47]	2013	retrospective cohort study	USA, Singapore	no	*P. aeruginosa*	bacteremia	empiric	all-cause 30-day/1 month mortality
Capone [48]	2013	prospective cohort study	Italy	no	Ertapenem-resistant *K. pneumoniae*	unrestricted	unclear	all-cause in-hospital mortality
Cardoso [49]	2013	prospective cohort study	Portugal	yes	unrestricted	unrestricted	empiric	all-cause in-hospital mortality
Ferreira [50]	2013	retrospective cohort study	Portugal	no	unrestricted	infective endocarditis	overall	all-cause in-hospital mortality
Gasch [51, 52]	2013	prospective cohort study	Spain	no	MRSA	bacteremia	empiric	- all-cause 2-day mortality<br>- all-cause 30-day/1 month mortality
Heintz [53]	2013	retrospective cohort study	United States	no	VRE	urinary tract infection	- empiric<br>- definite	clinical failure (extended antibiotic therapy within 30 days, positive follow-up blood culture within 7 days of completing treatment, 30-day infection-related readmission or 30-day all-cause mortality)
Horcajada [54]	2013	prospective cohort study	Spain	yes	*Enterobacteriaceae*, *P. aeruginosa*, non-fermenting gram-negative bacilli, *Enterococcus* spp., *S. saprophyticus*	bacteremic urinary tract infection	empiric	all-cause 30-day/1 month mortality
Kang [55]	2013	retrospective cohort study	South Korea	yes	unrestricted	bacteremic biliary tract infection	- empiric<br>- definite, no definition	all-cause 30-day/1 month mortality
Kang [56]	2013	retrospective cohort study	South Korea	no	ESBL-producing *E. coli*	bacteremia	empiric	- all-cause 30-day/1 month mortality

								- clinical failure (no improvement, deterioration of clinical parameters or death by day 3)
Kuo [57]	2013	retrospective cohort study	Taiwan	yes	*A. nosocomialis*	bacteremia	empiric	all-cause 14-day mortality
Lee [58]	2013	retrospective cohort study	Taiwan	yes	unrestricted	bacteremia	empiric	all-cause 28-day mortality
Lee [59]	2013	unclear/unknown	Taiwan	no	*A. baumannii*, *A. nosocomialis*	bacteremic hospital-acquired pneumonia	empiric	all-cause 14-day mortality
Metan [60]	2013	retrospective cohort study	Turkey	no	gram-negative bacteria	bacteremia	empiric	all-cause 7-day mortality
Navarro-San Francisco [61]	2013	prospective cohort study	Spain	no	carbapenemase-producing Enterobacteriaceae	bloodstream infection	other	all-cause 30-day/1 month mortality
Palmer [62]	2013	prospective cohort study	United States	no	gram-negative bacteria	bacteremia	empiric	all-cause in-hospital mortality
Park [63]	2013	retrospective cohort study	South Korea	yes	*P. aeruginosa*, *A. baumannii*	bacteremia	- empiric<br>- definite, no definition	all-cause 14-day mortality
Peña [64]	2013	retrospective cohort study	Spain	no	*P. aeruginosa*	ventilator-associated lower respiratory tract infection	- empiric<br>- definite	- all-cause 7-day mortality<br>- all-cause in-hospital mortality
Phua [65]	2013	prospective cohort study	Singapore	no	unrestricted	severe sepsis	empiric	all-cause in-hospital mortality
Retamar [66]	2013	prospective cohort study	Spain	no	unrestricted	bloodstream infection	empiric	- all-cause 14-day mortality<br>- all-cause 30-day/1 month mortality
Ruiz-Giardin [67]	2013	retrospective cohort study	Spain	yes	unrestricted	bacteremia	empiric	all-cause mortality
Shorr [68]	2013	retrospective cohort study	United States	no	unrestricted	non-nosocomial pneumonia	empiric	all-cause hospital readmission

Tumbarello [69]	2013	retrospective / secondary analysis of prospective data	Italy	yes	*P. aeruginosa*	hospital-acquired pneumonia, healthcare-associated pneumonia	empiric	- all-cause ICU mortality<br>- duration of mechanical ventilation
Vallés [70]	2013	retrospective / secondary analysis of prospective data	Spain	no	unrestricted	bacteremia	empiric	all-cause ICU mortality
Willmann [71]	2013	retrospective cohort study	Germany	no	*P. aeruginosa*	bloodstream infection	- empiric<br>- definite	- all-cause in-hospital mortality<br>- in-hospital length of stay
Yang [72]	2013	retrospective cohort study	Taiwan	yes	unrestricted	bacteremia	empiric	all-cause in-hospital mortality
Yang [73]	2013	retrospective cohort study	Taiwan	no	*A. baumannii*, *A. nosocomialis*	bloodstream infection	empiric	all-cause 14-day mortality
Zheng [74]	2013	retrospective cohort study	China	no	*A. baumannii*	nosocomial pneumonia	empiric	all-cause 28-day mortality
Anderson [75]	2014	retrospective cohort study	United States	yes	unrestricted	bloodstream infection	empiric	- all-cause in-hospital mortality<br>- ICU admission<br>- in-hospital length of stay<br>- post-infection hospital length of stay<br>- all-cause hospital readmission
Bloos [76]	2014	prospective cohort study	Germany	yes	unrestricted	severe sepsis or septic shock	empiric	all-cause 28-day mortality
Chusri [77]	2014	retrospective cohort study	Thailand	no	*A. calcoaceticus-baumannii* complex	unrestricted	empiric	all-cause 30-day/1 month mortality
Corrêa [78]	2014	secondary data from an RCT	Brazil	no	unrestricted	ventilator-associated pneumonia	empiric	all-cause 28-day mortality

De Bus [79]	2014	retrospective cohort study	Belgium	no	unrestricted	hospital-acquired pneumonia	empiric	- all-cause ICU mortality<br>- ICU length of stay
Esparcia [80]	2014	retrospective cohort study	Spain	yes	unrestricted	urinary tract infection	empiric	all-cause in-hospital mortality
Falcone [81]	2014	prospective cohort study	Italy	no	ESBL-producing Enterobacteriaceae	bloodstream infection	empiric	all-cause 21-day mortality
Fayad [82]	2014	retrospective cohort study	France	no	unrestricted	infective endocarditis	other	all-cause in-hospital mortality
Garnacho-Montero [83]	2014	prospective cohort study	Spain	no	unrestricted	severe sepsis or septic shock	empiric	all-cause in-hospital mortality
Girometti [84]	2014	retrospective cohort study	Italy	yes	*K. pneumoniae*	bloodstream infection	empiric	all-cause 30-day/1 month mortality
Gonçalves-Pereira [85]	2014	prospective cohort study	Portugal	no	unrestricted	unrestricted	empiric	all-cause in-hospital mortality
Hsu [86]	2014	retrospective cohort study	Taiwan	no	*S. aureus*	bacteremia	empiric	all-cause in-hospital mortality
Jeong [87]	2014	retrospective / secondary analysis of prospective data	South Korea	no	unrestricted	community-acquired severe pneumonia, healthcare-associated severe pneumonia	empiric	all-cause in-hospital mortality
Kim [88]	2014	retrospective cohort study	South Korea	yes	*P. aeruginosa*	bacteremia	- empiric<br>- definite	all-cause 14-day mortality
Lee [89]	2014	retrospective cohort study	Taiwan	no	*A. baumannii* complex	bacteremia	empiric	infection-related 30-day/1 month mortality
Lipsky [90]	2014	prospective cohort study	United States	yes	unrestricted	complicated skin and soft tissue infection	empiric	in-hospital length of stay
Membrilla-Fernández [91]	2014	prospective cohort study	Spain	yes	unrestricted	secondary peritonitis	empiric	- all-cause mortality<br>- clinical failure (no complete resolution of all symptoms of infection)

Nygård [92]	2014	prospective cohort study	Norway	no	unrestricted	severe sepsis	2 definitions for empiric	all-cause in-hospital mortality
Park [93]	2014	prospective cohort study	South Korea	no	unrestricted	severe sepsis or septic shock	empiric, no definition	all-cause 28-day mortality
Pelegrín [94]	2014	retrospective / secondary analysis of prospective data	Spain	no	*L. monocytogenes*	meningoencephalitis	empiric	all-cause in-hospital mortality
Shorr [95]	2014	retrospective cohort study	United States	yes	*Acinetobacter* spp.	sepsis	empiric	all-cause in-hospital mortality
Spoorenberg [96]	2014	retrospective cohort study	Netherlands	yes	unrestricted	complicated urinary tract infection	overall	in-hospital length of stay
Vallés [97]	2014	prospective cohort study	Spain	no	unrestricted	severe community-acquired pneumonia, severe healthcare-associated pneumonia	empiric	all-cause ICU mortality
Van Aken [98]	2014	retrospective case cohort based on case control study	Sweden	no	*E. coli*	bacteremia	empiric	- all-cause 14-day mortality<br>- post-infection hospital length of stay
Vilella [99]	2014	retrospective cohort study	United States	yes	unrestricted	sepsis	empiric	- all-cause in-hospital mortality<br>- ICU length of stay
Yokota [100]	2014	retrospective cohort study	Brazil	yes	unrestricted	severe sepsis or septic shock	empiric	all-cause in-hospital mortality
Zeng [101]	2014	retrospective cohort study	China	no	gram-negative bacteria	severe sepsis or septic shock	definite	all-cause 30-day/1 month mortality
Zilberberg [102]	2014	retrospective cohort study	United States	yes	gram-negative bacteria	severe sepsis or septic shock	empiric	all-cause in-hospital mortality
Al-Dorzi [103]	2015	retrospective cohort study	Saudi Arabia	yes	*Acinetobacter* spp.	bacteremia	empiric	- all-cause in-hospital mortality<br>- all-cause ICU mortality

								- in-hospital length of stay<br>- ICU length of stay<br>- duration of mechanical ventilation
Allou [104]	2015	prospective cohort study	France	no	unrestricted	postoperative pneumonia	empiric	all-cause in-hospital mortality
Andria [105]	2015	retrospective cohort study	Israel	no	gram-negative aerobic bacteria	bacteremia	empiric	- all-cause 14-day mortality<br>- all-cause 1-year mortality
Bass [106]	2015	retrospective cohort study	United States	yes	carbapenem-resistant gram-negative bacteria	septic bloodstream infection	unclear	all-cause 30-day/1 month mortality
Bastug [107]	2015	retrospective cohort study	Turkey	no	unrestricted	sterile site infection, bloodstream infection, urinary tract infection	empiric	all-cause 30-day/1 month mortality
Beuving [108]	2015	Secondary data from an RCT	Netherlands	yes	gram-positive cocci, facultative aerobic gram-negative rods	bloodstream infection	unclear	- all-cause 30-day in-hospital mortality<br>- in-hospital length of stay
Boel [109]	2015	retrospective cohort study	Denmark	yes	unrestricted	bacteremia	empiric	- all-cause 30-day/1 month mortality<br>- all-cause hospital readmission<br>- acute hospital readmission<br>- infection-related hospital readmission
Brigmon [110]	2015	retrospective cohort study	United States	no	aerobic gram-negative bacteria	bloodstream infection	empiric	in-hospital length of stay

Coccolini [111]	2015	retrospective / secondary analysis of prospective data	multinational	no	unrestricted	intraabdominal infection secondary to acute cholecystitis	empiric	- all-cause mortality<br>- ICU admission
Denis [112]	2015	retrospective case cohort based on case control study	France	no	*E. coli*	bacteremia	empiric	all-cause 21-day mortality
Dimopoulos [113]	2015	retrospective / secondary analysis of prospective data	Greece	no	unrestricted	bloodstream infection	empiric	all-cause 28-day mortality
Inchai [114]	2015	retrospective cohort study	Thailand	no	unrestricted	ventilator-associated pneumonia	empiric	all-cause 30-day/1 month mortality
Katsiari [115]	2015	prospective cohort study	Greece	no	carbapenemase-producing *K. pneumoniae*	unrestricted	empiric	infection-related 14-day mortality
Lee [116]	2015	retrospective cohort study	Taiwan	no	MRSA	bacteremia	empiric	infection-related 30-day/1 month mortality
Martin-Loeches [117]	2015	prospective cohort study	Spain	no	unrestricted	ICU-acquired pneumonia	empiric	all-cause ICU mortality
Martin-Loeches [118]	2015	prospective cohort study	Spain, France, Portugal, Brazil, Argentina, Ecuador, Bolivia, Colombia	no	unrestricted	ventilator-associated lower respiratory tract infection	empiric	- all-cause ICU mortality<br>- clinical failure (progression from tracheobronchitis to pneumonia)
Oliveira [119]	2015	retrospective case cohort based on case control study	Brazil	no	Enterobacteriaceae	unrestricted	empiric	- all-cause in-hospital mortality
Park [120]	2015	retrospective cohort study	South Korea	yes	coagulase-negative staphylococci	bacteremia	empiric	- all-cause 30-day/1 month mortality<br>- infection-related 30-day/1 month mortality

Rabello [121]	2015	retrospective / secondary analysis of prospective data	Brazil	no	unrestricted	community-acquired pneumonia, healthcare-associated pneumonia	empiric	all-cause in-hospital mortality
Ratzinger [122]	2015	prospective cohort study	Austria	yes	unrestricted	sepsis	empiric	all-cause in-hospital mortality
Shindo [123]	2015	prospective cohort study	Japan	yes	unrestricted	community-acquired pneumonia, healthcare-associated pneumonia	empiric	- all-cause 30-day/1 month mortality<br>- all-cause in-hospital mortality
Su [124]	2015	retrospective cohort study	Taiwan	no	cefepime-resistant *P. aeruginosa*	bacteremia	empiric	- all-cause 30-day/1 month mortality<br>- all-cause 15-day mortality
Suberviola Cañas [125]	2015	prospective cohort study	Spain	yes	unrestricted	septic shock	unclear	all-cause in-hospital mortality
Sumida [126]	2015	retrospective case cohort based on case control study	Japan	no	*S. maltophilia*, *P. aeruginosa*	bacteremia	unclear	all-cause 30-day/1 month mortality
Torres [127]	2015	prospective cohort study	Spain	no	unrestricted	community-acquired pneumonia	empiric	all-cause 30-day in-hospital mortality
Tumbarello [128]	2015	retrospective cohort study	Italy	no	KPC-producing *K. pneumoniae*	unrestricted	empiric	all-cause 14-day mortality
Wu [129]	2015	retrospective cohort study	China	no	unrestricted	bloodstream infection	empiric	all-cause 28-day mortality
Abraham [130]	2016	retrospective cohort study	United States	yes	unrestricted	bacteremia	empiric	- all-cause in-hospital mortality<br>- in-hospital length of stay<br>- ICU length of stay

								- duration of mechanical ventilation
Ali [131]	2016	retrospective cohort study	Qatar	no	unrestricted	ventilator-associated pneumonia	empiric	all-cause 30-day/1 month mortality
Cheng [132]	2016	retrospective cohort study	Taiwan	no	ESBL-producing *E. coli* or *K. pneumoniae*	bacteremic pneumonia	- empiric<br>- definite	all-cause 30-day/1 month mortality
Chin [133]	2016	retrospective cohort study	Canada	yes	unrestricted	ventilator-associated pneumonia	empiric	- all-cause in-hospital mortality<br>- all-cause ICU mortality<br>- ICU length of stay<br>- duration of mechanical ventilation
Coccolini [134]	2016	retrospective / secondary analysis of prospective data	multinational	no	unrestricted	intraabdominal infection secondary to acute appendicitis	empiric, no definition	- all-cause 30-day/1 month mortality<br>- ICU admission
Cuervo [135]	2016	prospective cohort study	Spain	no	MRSA	bacteremia	empiric	- all-cause 2-day mortality<br>- all-cause 30-day/1 month mortality
De Rosa [136]	2016	retrospective cohort study	Italy	no	*S. aureus*	bloodstream infection	definite	all-cause 21-day mortality
Fitzpatrick [137]	2016	prospective cohort study	United Kingdom	yes	*E. coli*, *Klebsiella* spp., *Enterobacter* spp., *Serratia* spp., *Morganella* spp., *Citrobacter* spp., *Proteus* spp., *Pseudomonas* spp.	bacteremia	empiric	- all-cause 7-day mortality<br>- all-cause 30-day/1 month mortality
Freire [138]	2016	retrospective cohort study	Brazil	no	extensively drug resistant *A.*	bacteremia	unclear	all-cause 30-day/1 month mortality

					baumannii-calcoaceticus complex			
Garnacho-Montero [139]	2016	prospective cohort study	Spain	no	*A. baumannii*	unrestricted	empiric	all-cause 30-day/1 month mortality
Gonzalez [140]	2016	retrospective cohort study	France	no	unrestricted	septic shock	empiric	all-cause ICU mortality
Guilbart [141]	2016	prospective cohort study	France	no	unrestricted	complicated intra-abdominal infection	2 definitions for empiric	- all-cause in-hospital mortality<br>- ICU admission<br>- in-hospital length of stay<br>- ICU length of stay<br>- adverse events (infectious complications)
Guillamet [142]	2016	retrospective cohort study	United States	no	unrestricted	bacteremic pneumonia	empiric	all-cause in-hospital mortality
Herkel [143]	2016	prospective cohort study	Czech Republic	yes	unrestricted	pneumonia	empiric	all-cause 30-day/1 month mortality
Li [144]	2016	retrospective cohort study	China	no	unrestricted	pneumonia	empiric	clinical failure (slowly resolving pneumonia)
Li [145]	2016	retrospective cohort study	China	no	unrestricted	meningitis, ventriculitis	empiric	all-cause in-hospital mortality
Maeda [146]	2016	Secondary data from quasi-experimental study	Japan	no	unrestricted	bloodstream infection	definite	all-cause in-hospital mortality
Oshima [147]	2016	retrospective cohort study	Japan	yes	unrestricted	severe sepsis or septic shock	empiric	all-cause ICU mortality
Palacios-Baena [148]	2016	retrospective cohort study	Spain	no	carbapenemase-producing Enterobacteriaceae	Unrestricted	- empiric<br>- definite	all-cause 30-day/1 month mortality
Ruangchan [149]	2016	retrospective cohort study	Thailand	no	unrestricted	severe sepsis or septic shock	empiric	all-cause mortality
Savage [150]	2016	retrospective / secondary analysis	Canada	yes	unrestricted	bloodstream infection	empiric	all-cause in-hospital mortality

		of retrospective data						
Stoma [151]	2016	prospective cohort study	Belarus	no	unrestricted	bloodstream infection	empiric	all-cause 30-day/1 month mortality
Trecarichi [152]	2016	prospective cohort study	Italy	no	*K. pneumoniae*	bloodstream infection	empiric	all-cause 21-day mortality
Vallés [153]	2016	retrospective cohort study	Spain	no	unrestricted	severe community-acquired pneumonia	empiric	all-cause ICU mortality
Worapratya [154]	2016	prospective cohort study	Thailand	yes	unrestricted	septic shock	empiric	all-cause in-hospital mortality
Yilmaz [155]	2016	prospective cohort study	Turkey	no	*S. aureus*	bacteremia	empiric	all-cause 28-day in-hospital mortality
Yoon [156]	2016	prospective cohort study	South Korea	yes	MRSA	healthcare-associated bacteremia	empiric	- all-cause in-hospital mortality<br>- infection-related in-hopital mortality
Zarco-Márquez [157]	2016	retrospective cohort study	Mexico	no	*S. pneumoniae*	unrestricted	empiric	all-cause 30-day/1 month mortality
Zilberberg [158]	2016	retrospective cohort study	United States	yes	*A. baumannii*	pneumonia, sepsis	empiric	all-cause in-hospital mortality
Ahn [159]	2017	retrospective cohort study	South Korea	no	unrestricted	community-acquired pneumonia, healthcare-associated pneumonia	empiric	all-cause 28-day mortality
Babich [160]	2017	prospective cohort study	Israel	yes	unrestricted	catheter-associated urinary tract infection	empiric	all-cause 30-day/1 month mortality
Battle [161]	2017	retrospective cohort study	United States	yes	aerobic gram-negative bacteria	bloodstream infection	empiric	in-hospital length of stay
Bosch-Nicolau [162]	2017	retrospective cohort study	Spain	yes	unrestricted	pyelonephritis, septic urinary tract infection	- empiric<br>- definite, no definition	- all-cause mortality<br>- in-hospital length of stay
Costa-de-Oliveira [163]	2017	retrospective cohort study	Portugal	no	unrestricted	bloodstream infection	empiric	- all-cause mortality

								- in-hospital length of stay
Deconinck [164]	2017	retrospective cohort study	France	yes	*P. aeruginosa*	ventilator-associated pneumonia	empiric	all-cause ICU mortality
González-Del Castillo [165]	2017	retrospective cohort study	Spain	yes	unrestricted	unrestricted	empiric	- all-cause 30-day/1 month mortality<br>- in-hospital length of stay<br>- all-cause hospital readmission
Goto [166]	2017	retrospective cohort study	United States	no	*S. aureus*	bacteremia	definite	all-cause 30-day/1 month mortality
Gutiérrez-Gutiérrez [167]	2017	retrospective cohort study	multinational	yes	carbapenemase-producing Enterobacteriaceae	bloodstream infection	unclear	all-cause 30-day/1 month mortality
Jokinen [168]	2017	retrospective cohort study	Finland	no	*S. aureus*	bacteremia	empiric	all-cause 28-day mortality
Joo [169]	2017	retrospective cohort study	South Korea	yes	ESBL-producing *E. coli* or *K. pneumoniae*	bacteremia	empiric	all-cause 30-day/1 month mortality
Lachhab [170]	2017	prospective cohort study	Morocco	no	unrestricted	bacteremia	unclear, no definition	all-cause ICU mortality
Li [171]	2017	retrospective cohort study	China	no	*K. pneumoniae*	bloodstream infection	- empiric<br>- definite	all-cause 30-day/1 month mortality
Micozzi [172]	2017	retrospective cohort study	Italy	no	Carbapenem-resistant *K. pneumoniae*	unrestricted	- empiric<br>- definite	all-cause 30-day/1 month mortality
Póvoa [173]	2017	prospective cohort study	Spain	yes	unrestricted	ventilator-associated pneumonia	empiric	all-cause ICU mortality
Palacios-Baena [174]	2017	retrospective cohort study	Spain, Germany, Italy, Greece, Israel,	yes	ESBL-producing Enterobacteriaceae	bloodstream infection	- empiric<br>- definite	all-cause 30-day/1 month mortality

			Turkey, South Africa, Canada, United States, Argentina, Taiwan					
Papadimitriou-Olivgeris [175]	2017	retrospective case cohort based on case control study	Greece	no	carbapenemase-producing *K. pneumoniae*	bacteremia	empiric	all-cause 30-day/1 month mortality
Pouwels [176]	2017	retrospective cohort study	United Kingdom	yes	Enterobacteriaceae	bacteremia	empiric	all-cause ICU mortality
Rello [177]	2017	retrospective cohort study	multinational	no	unrestricted	community-acquired pneumonia	unclear	all-cause ICU mortality
Royo-Cebrecos [178]	2017	prospective cohort study	Spain	no	unrestricted	bacteremic cholangitis	empiric	all-cause 30-day/1 month mortality
Tagashira [179]	2017	retrospective cohort study	Japan	yes	unrestricted	bacteremic cholangitis	empiric	all-cause 30-day/1 month mortality
Thaden [180]	2017	prospective cohort study	United States	no	*S. aureus*, gram-negative bacteria	bloodstream infection	unclear	all-cause in-hospital mortality
Tuon [181]	2017	retrospective cohort study	Brazil	no	carbapenem-resistant Enterobacteriaceae	Ventilator-associated pneumonia	- empiric<br>- definite	all-cause 30-day/1 month mortality
Wang [182]	2017	retrospective cohort study	China	no	*A. baumannii*	bacteremia	empiric	all-cause 30-day/1 month mortality
Zhang [183]	2017	retrospective cohort study	China	no	Enterococci	bloodstream infection	empiric	- all-cause 7-day mortality<br>- all-cause 30-day/1 month mortality
Zilberberg [184]	2017	retrospective cohort study	United States	yes	Enterobacteriaceae	urinary tract infection, pneumonia, sepsis	empiric	- all-cause in-hospital mortality<br>- in-hospital length of stay
Abdulsalam [185]	2018	prospective cohort study	India	no	*S. aureus*	bacteremia	empiric	all-cause 30-day/1 month mortality

Bassetti [186]	2018	retrospective cohort study	Italy	no	*S. aureus*	bacteremia	empiric	- all-cause 7-day mortality<br>- all-cause 30-day/1 month mortality
Bouiller [187]	2018	prospective cohort study	France	no	MSSA	Bacteremia	- empiric<br>- definite<br>- overall	all-cause 30-day/1 month mortality
Chen [188]	2018	retrospective case cohort based on case control study	Taiwan	no	*A. baumannii*	bloodstream infection	empiric	all-cause 14-day mortality
Claeys [189]	2018	retrospective cohort study	United States	yes	gram-negative bacteria	lower respiratory tract infection	- empiric<br>- definite	- all-cause 30-day/1 month mortality<br>- all-cause in-hospital mortality<br>- in-hospital length of stay<br>- ICU length of stay<br>- infection-related hospital readmission
Dewi [190]	2018	retrospective cohort study	Indonesia	yes	unrestricted	sepsis or septic shock	unclear	all-cause mortality
Fouks [191]	2018	retrospective cohort study	Israel	yes	unrestricted	surgical site infection	empiric	in-hospital length of stay
Garcia-Vidal [192]	2018	prospective cohort study	Spain	no	unrestricted	bloodstream infection	empiric	all-cause 30-day/1 month mortality
Garrouste-Orgeas [193]	2018	retrospective / secondary analysis of prospective data	France	no	*S. pneumoniae*	invasive pneumococcal infection	empiric	all-cause 28-day mortality
Holmes [194]	2018	prospective cohort study	Australia	no	*S. aureus*	bacteremia	empiric	clinical failure (all-cause mortality, persistent bacteraemia at 7 days or recurrent bacteraemia within 30 days)
Islas-Muñoz [195]	2018	prospective cohort study	Mexico	no	unrestricted	bloodstream infection	empiric	all-cause 30-day/1 month mortality

Kethireddy [196]	2018	retrospective nested cohort study	Canada, USA, Saudi Arabia	no	unrestricted	septic shock	empiric	all-cause in-hospital mortality
Lee [197]	2018	retrospective cohort study	Taiwan	no	*Acinetobacter* spp.	catheter-related bloodstream infection	empiric	all-cause 30-day/1 month mortality
Li [198]	2018	retrospective cohort study	China	no	unrestricted	ventilator-associated pneumonia	empiric	all-cause 28-day mortality
Papadopoulos [199]	2018	prospective cohort study	Greece	no	unrestricted	aspiration pneumonia	empiric	clinical failure (no clinical response within 72h of treatment)
Saliba [200]	2018	prospective cohort study	Spain	no	unrestricted	vascular catheter-related bloodstream infections	empiric	all-cause 30-day/1 month mortality
Seas [201]	2018	prospective cohort study	Argentina, Brazil, Chile, Colombia, Ecuador, Guatemala, Mexico, Peru, Venezuela	yes	*S. aureus*	bacteremia	definite	all-cause 30-day/1 month mortality
Sommer [202]	2018	retrospective / secondary analysis of prospective data	France	yes	*P. aeruginosa*	ventilator-associated pneumonia	empiric	clinical failure (no extubation or in-hospital mortality)
Tang [203]	2018	retrospective cohort study	China	no	unrestricted	bloodstream infection	empiric	all-cause 30-day/1 month mortality
Tschudin-Sutter [204]	2018	retrospective cohort study	Switzerland	no	*P. aeruginosa*	bloodstream infection	empiric	- all-cause in-hospital mortality<br>- infection-related in-hospital mortality
Xu [205]	2018	retrospective cohort study	China	no	unrestricted	pneumonia	empiric	- all-cause 30-day/1 month mortality<br>- all-cause 90-day/3 month mortality

Yamaga [206]	2018	retrospective cohort study	Japan	yes	unrestricted	bloodstream infection	empiric	- all-cause 28-day mortality<br>- all-cause in-hospital mortality<br>- all-cause 60-day/2 month mortality<br>- all-cause ICU mortality
Battle [207]	2019	retrospective cohort study	United States	yes	gram-negative bacteria	bloodstream infection	empiric	all-cause 14-day mortality
Benítez-Sala [208]	2019	prospective cohort study	Spain	yes	unrestricted	unrestricted	empiric, no definition	all-cause mortality
Ben-Chetrit [209]	2019	retrospective cohort study	Israel	no	*E. coli*, *K. pneumoniae*	febrile neutropenia	empiric	all-cause 14-day mortality
Ben-Zvi [210]	2019	Secondary data from quasi-experimental study	Israel	no	*S. aureus*	bacteremia	empiric	all-cause 30-day/1 month mortality
Brescini [211]	2019	retrospective cohort study	Italy	no	KPC-producing *K. pneumoniae*	bloodstream infection	- empiric<br>- definite	all-cause 30-day/1 month mortality
Callejas-Díaz [212]	2019	retrospective cohort study	Spain	no	*P. aeruginosa*	bacteremia	- empiric<br>- definite	- all-cause mortality<br>- attributable mortality
Castaño [213]	2019	prospective cohort study	Colombia	yes	unrestricted	severe sepsis or septic shock	empiric	- all-cause in-hospital mortality<br>- in-hospital length of stay<br>- ICU length of stay<br>- ICU admission<br>- mechanical ventilation<br>- adverse events (renal replacement therapy)
Chusri [214]	2019	retrospective cohort study	Thailand	no	*A. baumannii*	bacteremia	empiric	all-cause 30-day/1 month mortality

Delle Rose [215]	2019	prospective cohort study	Italy	no	gram-negative bacteria	bloodstream infection	empiric	- all-cause in-hospital mortality<br>- all-cause 14-day mortality
Eliakim-Raz [216]	2019	retrospective cohort study	Bulgaria, Greece, Hungary, Israel, Italy, Romania, Spain, Turkey	yes	unrestricted	complicated urinary tract infection	empiric	- all-cause 30-day/1 month mortality<br>- clinical failure (continuous symptoms by day 5-7 of AAT, new infection-related symptoms or urine culture growing original pathogen within 30 days, 30-day mortality)
Gómez Belda [217]	2019	prospective cohort study	Spain	yes	unrestricted	bacteremic urinary tract infection	empiric	all-cause 30-day/1 month mortality
Huang [218]	2019	retrospective cohort study	Taiwan	no	*E. meningoseptica*, carbapenem-resistant *A. baumannii-calcoaceticus* complex, *P. aeruginosa* or *S. maltophilia*	bloodstream infection	definite	all-cause 28-day mortality
Jeon [219]	2019	retrospective cohort study	South Korea	no	unrestricted	sepsis	empiric	all-cause in-hospital mortality
Kim [220]	2019	retrospective cohort study	South Korea	yes	ESBL-producing Enterobacteriales	pyelonephritis	empiric	- in-hospital length of stay<br>- clinical failure (symptoms not resolved within 7 days or recurrence during antibiotic treatment, negative

								urine culture within 7 days)<br>- adverse events (composite variable)
Lat [221]	2019	prospective cohort study	United States	no	unrestricted	pneumonia	empiric	all-cause in-hospital mortality
Lim [222]	2019	retrospective cohort study	Australia	yes	ESBL-producing Enterobacteriaceae, AmpC-producing Enterobacteriaceae	bacteremia	- empiric<br>- definite	all-cause 30-day/1 month mortality
Maruyama [223]	2019	prospective cohort study	Japan	no	unrestricted	pneumonia	empiric	all-cause 30-day/1 month mortality
Morvan [224]	2019	retrospective / secondary analysis of prospective data	France	yes	*Enterococcus* spp.	intraabdominal infection	empiric	- all-cause 30-day/1 month mortality<br>- adverse events (infectious complications)<br>- adverse events (relaparotomy or percutaneous drainage)<br>- adverse events (surgical complications)
Park [225]	2019	retrospective cohort study	South Korea	no	Carbapenemase-producing Enterobacteriaceae	bloodstream infection	empiric	all-cause 30-day/1 month mortality
Ramos-Rincón [226]	2019	retrospective cohort study	Spain	no	unrestricted	bloodstream infection	empiric	all-cause in-hospital mortality
Rodriguez-Gómez [227]	2019	retrospective cohort study	Spain	yes	*K. pneumoniae*	urinary tract infection	empiric	- all-cause 30-day/1 month mortality<br>- clinical failure (persistence or recurrence of symptoms, death

								within 21 days without clinical response)
Schuttevaer [228]	2019	retrospective cohort study	Netherlands	yes	unrestricted	bloodstream infection	empiric	all-cause 30-day/1 month mortality
Shi [229]	2019	retrospective cohort study	China	no	*P. aeruginosa*	bloodstream infection	- empiric<br>- definite	all-cause 30-day/1 month mortality
Tacconelli [230]	2019	prospective cohort study	Italy	yes	ESBL-producing Enterobacteriaceae	unrestricted	empiric	- all-cause in-hospital mortality<br>- in-hospital length of stay
Wang [231]	2019	retrospective cohort study	China	no	carbapenem-resistant Enterobacteriaceae	bloodstream infection	definite	all-cause in-hospital mortality
Wiggers [232]	2019	retrospective cohort study	Canada	yes	unrestricted	bacteremic urinary tract infection	empiric	- all-cause 30-day/1 month mortality<br>- time to clinical success
Al-Sunaidar [233]	2020	retrospective cohort study	Malaysia	yes	unrestricted	sepsis	empiric	- all-cause ICU mortality<br>- ICU length of stay
Augustin [234]	2020	retrospective cohort study	France	yes	unrestricted	post-operative peritonitis	empiric	all-cause in-hospital mortality
Babich [235]	2020	retrospective cohort study	Australia, Germany, Greece, France, Israel, Slovenia, Spain, Sweden, United Kingdom	no	*P. aeruginosa*	bacteremia	empiric	all-cause 30-day/1 month mortality
Benetazzo [236]	2020	retrospective cohort study	France	no	ESBL-producing Enterobacteriaceae	bloodstream infection	empiric	all-cause 30-day/1 month mortality

Chen [237]	2020	retrospective cohort study	Taiwan	no	unrestricted	bacteremia	empiric	all-cause 30-day/1 month mortality
Chen [238]	2020	retrospective cohort study	Taiwan	yes	*E. coli*, *K. pneumoniae*	bloodstream infection	empiric	all-cause in-hospital mortality
Dubler [239]	2020	retrospective cohort study	Germany	no	*E. faecium*	bloodstream infection	empiric	- all-cause 30-day/1 month mortality<br>- clinical failure (death or persistent stay in the ICU)
Falcone [240]	2020	retrospective cohort study	Italy	yes	KPC-producing *K. pneumoniae*	bloodstream infection	unclear	all-cause 30-day/1 month mortality
Ingram [241]	2020	retrospective cohort study	Australia	no	unrestricted	infective endocarditis	other	clinical failure (unplanned cardiac surgery, embolic event, relapse of positive blood culture or death within 6 months)
Kang [242]	2020	retrospective cohort study	Taiwan	yes	*A. baumannii*	bacteremic pneumonia	empiric	all-cause 14-day mortality
Kawasuji [243]	2020	retrospective cohort study	Japan	no	MRSA	bacteremia	empiric	all-cause in-hospital mortality
Kim [244]	2020	retrospective cohort study	South Korea	yes	unrestricted	community-acquired pneumonia, healthcare-associated pneumonia	empiric	- all-cause 30-day/1 month mortality<br>- in-hospital length of stay<br>- ICU length of stay
Lambregts, Wijnakker [245]	2020	retrospective cohort study	Netherlands	yes	unrestricted	bloodstream infection	empiric	- all-cause 14-day mortality<br>- all-cause 30-day/1 month mortality<br>- in-hospital length of stay
Lee [246]	2020	retrospective cohort study	Taiwan	no	unrestricted	bacteremia	empiric	all-cause 30-day/1 month mortality

Lee [247]	2020	retrospective cohort study	Taiwan	yes	*S. aureus*, streptococci	bacteremia	empiric	all-cause 28-day mortality
Malbasa [248]	2020	retrospective case cohort based on case control study	Serbia	no	multidrug-resistant *Acinetobacter* spp.	bacteremia	empiric	all-cause 30-day/1 month mortality
Martinez-Nadal [249]	2020	retrospective / secondary analysis of prospective data	Spain	yes	unrestricted	bacteremia in high-risk febrile neutropenia	empiric	all-cause 30-day/1 month mortality
Mitsuboshi [250]	2020	retrospective cohort study	Japan	no	ESBL-producing *E. coli*, *K. pneumoniae*, *P. mirabilis*	bacteremia	- empiric<br>- definite	all-cause 30-day/1 month mortality
Montero [251]	2020	retrospective cohort study	Spain	no	*P. aeruginosa*	bacteremia	- empiric<br>- definite, no definition	- all-cause 14-day mortality<br>- all-cause 30-day/1 month mortality
Mora-Guzmán [252]	2020	prospective cohort study	Spain	no	carbapenemase-producing Enterobacteriaceae	abdominal surgical site infection	- empiric<br>- definite	all-cause 30-day/1 month mortality
Papadimitriou-Olivgeris [253]	2020	retrospective cohort study	Greece	no	gram-positive cocci	bloodstream infection	empiric	all-cause 14-day mortality
Rhee [254]	2020	retrospective cohort study	United States	yes	unrestricted	sepsis	empiric	- all-cause in-hospital mortality<br>- adverse events (acute kidney injury)<br>- C. difficile infection rate
Righolt [255]	2020	retrospective cohort study	Canada	yes	unrestricted	complicated urinary tract infection, complicated intra-abdominal infection	empiric	- all-cause 30-day/1 month mortality<br>- in-hospital length of stay of at least 21 days<br>- ICU length of stay<br>- ICU admission<br>- all-cause hospital readmission

Rivera-Espinar [256]	2020	retrospective cohort study	Spain	no	*K. pneumoniae*	ventilator-associated pneumonia	- empiric<br>- definite	all-cause 30-day/1 month mortality
Santos [257]	2020	retrospective cohort study	Portugal	no	unrestricted	sepsis and septic shock	empiric	all-cause in-hospital mortality
Seo [258]	2020	retrospective cohort study	South Korea	no	carbapenem-resistant *E. coli* or *K. pneumoniae*	bacteremia	- empiric<br>- definite	all-cause 14-day mortality
Seok [259]	2020	prospective cohort study	South Korea	yes	unrestricted	sepsis	empiric	- all-cause 7-day mortality<br>- all-cause 14-day mortality<br>- all-cause 28-day mortality
Wagner [260]	2020	retrospective cohort study	United States	no	unrestricted	unrestricted	empiric	clinical failure (30-day readmission, adverse drug reaction before discharge alive, C. difficile infection within 30 days after discharge)
Wang [261]	2020	retrospective cohort study	Taiwan	no	*A. baumannii*	bacteremia	empiric	all-cause 14-day mortality
Wiener-Well [262]	2020	retrospective cohort study	Israel	yes	unrestricted	bacteremia	- 2 definitions for empiric<br>- unclear, no definition	- all-cause in-hospital mortality<br>- in-hospital length of stay<br>- clinical failure (prolonged time to defervescence or normalization of peripheral leucocyte count)
Xiao, Zhu [263]	2020	retrospective cohort study	China	no	*K. pneumoniae*	bloodstream infection	- empiric<br>- definite	all-cause 28-day mortality
Zhao [264]	2020	retrospective cohort study	China	no	*P. aeruginosa*	bloodstream infection	- empiric	all-cause 30-day/1 month mortality

							- definite, no definition	
Aliyu [265]	2021	retrospective cohort study	United States	yes	unrestricted	septic bloodstream infection	empiric	- all-cause in-hospital mortality or discharge to hospice<br>- in-hospital length of stay<br>- ICU length of stay<br>- ICU admission
Amipara [266]	2021	retrospective cohort study	United States	no	gram-negative bacteria	bloodstream infection	empiric	all-cause 28-day mortality
Cetin [267]	2021	retrospective cohort study	Turkey	no	gram-negative bacteria	bloodstream infection	empiric	all-cause 28-day mortality
Chang [268]	2021	prospective cohort study	South Korea	no	unrestricted	hospital-acquired pneumonia, ventilator-associated pneumonia	- empiric<br>- definite	- all-cause 28-day mortality<br>- all-cause 60-day/2 month mortality<br>- all-cause ICU mortality<br>- clinical failure (no complete patient recovery and stoppage of antibiotics for pneumonia on day 28)
D'Onofrio [269]	2021	prospective cohort study	Belgium	no	unrestricted	sepsis	empiric	- all-cause in-hospital mortality<br>- in-hospital length of stay<br>- ICU length of stay<br>- ICU admission
Gómez-Zorrilla [270]	2021	prospective cohort study	Spain	no	Enterobacterales, *P. aeruginosa*, non-fermenting gram-	bacteremic urinary tract infection	empiric	- all-cause 30-day/1 month mortality

	2021				negative bacteria, *S. saprophyticus*, *S. agalactiae*, *Enterococcus* spp.			- in-hospital length of stay<br>- clinical failure (symptoms of infection not completely resolved at discharge)
Jovanovic [271]	2021	prospective cohort study	Serbia	yes	unrestricted	ventilator-associated pneumonia	empiric	- all-cause in-hospital mortality<br>- infection-related in-hospital mortality<br>- ICU length of stay<br>- clinical failure (pneumonia-related sepsis)<br>- microbiological failure
Kadri [272]	2021	retrospective cohort study	United States	yes	unrestricted	bloodstream infection	empiric	all-cause in-hospital mortality or discharge to hospice
Kohler [273]	2021	retrospective case cohort based on case control study	Switzerland	no	Enterobacterales	unrestricted	empiric	clinical failure (microbiological relapse, graft failure or death within 90 days)
Liu [274]	2021	retrospective cohort study	Taiwan	yes	Carbapenem-resistant *K. pneumoniae*	bacteremia	- empiric<br>- definite	all-cause 30-day/1 month mortality
Man [275]	2021	retrospective cohort study	Hong Kong	yes	*K. pneumoniae*	bacteremia	empiric	- all-cause 90-day/3 month mortality<br>- all-cause in-hospital mortality<br>- all-cause ICU mortality<br>- in-hospital length of stay

								- duration of mechanical ventilation
Meng [276]	2021	retrospective cohort study	China	no	*A. baumannii* complex	bloodstream infection	- empiric<br>- definite	all-cause 28-day mortality
Moschou [277]	2021	prospective cohort study	Greece	no	unrestricted	bloodstream infection	empiric	all-cause 30-day/1 month mortality
Puzniak [278]	2021	retrospective cohort study	United States	yes	unrestricted	unrestricted	empiric	- all-cause in-hospital mortality<br>- in-hospital length of stay<br>- ICU length of stay
Quillici [279]	2021	retrospective cohort study	Brazil	yes	gram-negative bacteria	bloodstream infection	empiric	all-cause ICU mortality
Rodríguez [280]	2021	retrospective cohort study	Spain	no	*K. pneumoniae*	bacteremia	- empiric<br>- definite	all-cause 14-day mortality
Shen [281]	2021	retrospective cohort study	China	no	carbapenem-resistant *K. pneumoniae*	bloodstream infection	- empiric<br>- definite	all-cause 28-day mortality
Shorr [282]	2021	retrospective cohort study	United States	no	*S. pneumoniae*	community-acquired pneumonia	empiric	all-cause in-hospital mortality
Sun, Zhao [283]	2021	retrospective cohort study	China	no	*Aeromonas* spp.	bacteremia	empiric	clinical failure (discharge due to continuously deteriorating conditions or death)
Teelucksingh [284]	2021	retrospective cohort study	United States	yes	*P. aeruginosa*	bacteremia	empiric	clinical failure (in-hospital death, transition to hospice/palliative care)
Thy [285]	2021	retrospective cohort study	France	no	unrestricted	necrotizing skin and soft tissue infection	empiric	emergence of multidrug-resistant organisms
Yiang [286]	2021	retrospective cohort study	Taiwan	no	unrestricted	Suspected septic community-acquired or healthcare-	empiric	all-cause ICU mortality

						associated pneumonia		
Zhang [287]	2021	unclear/unknown	China	no	*E. coli*	bloodstream infection	empiric	all-cause in-hospital mortality
Zhu, Chen [288]	2021	retrospective cohort study	China	yes	*E. coli*	urinary tract infection	empiric	- all-cause 30-day/1 month mortality<br>- in-hospital length of stay<br>- clinical failure (recurrence of urinary tract infection)<br>- duration of antibiotic treatment
Zilberberg [289]	2021	retrospective cohort study	United States	yes	Enterobacteriaceae, *P. aeruginosa*, A. *baumannii*, *E. faecium*, *E. faecalis*	urinary tract infection	empiric	- all-cause in-hospital mortality<br>- in-hospital length of stay

a. Refers to the study design/data source used for our systematic review.

S Table 3: Overview of appropriate antibiotic therapy (AAT) definitions by study and respective aspects of AAT evaluated

First Author	Year	Treatment phase	Susceptibility	Timely start of therapy	Dosing	Route of administration	Duration	Guideline-based therapy	Restriction of aminoglycoside therapy	Other	Other aspects of appropriateness evaluated in the study	Total number of indicators
De Rosa [1]	2011	empiric	X	X								2
Fayad [2]	2011	unclear	X				X	X				3
Fernández-Hidalgo [3]	2011	overall	X		X	X		X		X	general rules for choice of empiric antibiotic	5
Johnson [4]	2011	empiric	X	X			X					3
Joo [5]	2011	empiric	X	X	X	X						4
Lin [6]	2011	empiric	X									1
Micek [7]	2011	empiric	X	X								2
Montravers [8]	2011	empiric	X	X								2
Plataki [9]	2011	empiric	X									1
Reisfeld [10]	2011	empiric	X		X							2
		definite	X		X							2
Rello [11]	2011	empiric	X									1
Schechner [12]	2011	empiric	X	X								2
Schreiber [13]	2011	empiric	X	X								2
Seligman [14]	2011	empiric	X									1

Shorr [15]	2011	empiric	X	X								2
Suppli [16]	2011	overall	X	X	X		X			X	no contraindactions/relevant interactions	5
Tumbarello [17]	2011	empiric	X									1
Wang [18]	2011	empiric	X									1
		definite	X									1
Zarkotou [19]	2011	empiric	X	X								2
		definite	X				X					2
Aguilar-Duran [20]	2012	empiric	X									1
Ariza [21]	2012	empiric	X									1
Bouza [22]	2012	empiric	X	X								2
Castillo [23]	2012	empiric	X	X	X	X	X					5
Chidiac [24]	2012	empiric								X	specific antibiotics that were considered (in)appropriate for the entire study cohort	1
		definite								X	specific antibiotics that were considered (in)appropriate for the entire study cohort	1
Chuang [25]	2012	unclear	X						X	X	- monotherapy with specific antibiotics other than aminoglycosides considered inappropriate<br>- specific antibiotics considered (in)appropriate in specific resistance pattern	4
de Gouvêa [26]	2012	empiric	X	X						X	specific antibiotics considered (in)appropriate in specific resistance pattern	3
Gözel [27]	2012	empiric	X	X								2
Halilovic [28]	2012	empiric	X	X								2
Hernández-Torres [29]	2012	empiric	X		X							2
		definite	X		X		X					3
Horino [30]	2012	empiric	X									1
Huang [31]	2012	empiric	X	X					X			3
Jung [32]	2012	empiric	X							X	specific antibiotics considered (in)appropriate in specific resistance pattern	2

		definite	X							X	specific antibiotics considered (in)appropriate in specific resistance pattern	2
Kim [33]	2012	definite	X	X	X	X						4
Lee [34]	2012	empiric	X									1
Lye [35]	2012	empiric	X		X							2
		definite	X		X							2
Micek [36]	2012	empiric	X	X			X					3
O'Neal [37]	2012	empiric	X	X						X	specific antibiotics considered (in)appropriate in specific resistance pattern	3
Park [38]	2012	empiric	X	X		X						3
Qureshi [39]	2012	empiric	X									1
Sancho [40]	2012	empiric	X									1
Silveira [41]	2012	empiric						X				1
Tabah [42]	2012	unclear	X							X	- rules for appropriateness when susceptibility testing was not performed/reported<br>- rules regarding combination therapy	3
Tumbarello [43]	2012	empiric	X	X				X				3
Wu [44]	2012	empiric	X	X								2
Zervos [45]	2012	empiric	X	X						X	rules for appropriateness when susceptibility testing was not performed/reported	3
Adrie [46]	2013	empiric	X									1
Bowers [47]	2013	empiric	X	X								2
Capone [48]	2013	unclear	X				X					2
Cardoso [49]	2013	empiric	X		X	X						3
Ferreira [50]	2013	overall					X	X				2
Gasch [51, 52]	2013	empiric	X						X			2
Heintz [53]	2013	empiric	X									1
		definite			X		X	X				3
Horcajada [54]	2013	empiric	X	X								2
Kang [55]	2013	empiric	X	X	X	X						4
Kang [56]	2013	empiric	X		X	X						3
Kuo [57]	2013	empiric	X	X	X	X						4

Lee [58]	2013	empiric	X									1
Lee [59]	2013	empiric	X	X	X	X			X			5
Metan [60]	2013	empiric	X		X	X				X	specific antibiotics considered (in)appropriate in specific resistance pattern	4
Navarro-San Francisco [61]	2013	other	X									1
Palmer [62]	2013	empiric	X									1
Park [63]	2013	empiric	X						X			2
Peña [64]	2013	empiric	X						X	X	monotherapy with specific antibiotics other than aminoglycosides considered inappropriate	3
		definite	X						X	X	monotherapy with specific antibiotics other than aminoglycosides considered inappropriate	3
Phua [65]	2013	empiric	X									1
Retamar [66]	2013	empiric	X	X	X							3
Ruiz-Giardin [67]	2013	empiric	X		X	X						3
Shorr [68]	2013	empiric	X	X								2
Tumbarello [69]	2013	empiric	X									1
Vallés [70]	2013	empiric	X	X								2
Willmann [71]	2013	empiric	X						X			2
		definite	X						X			2
Yang [72]	2013	empiric	X		X	X						3
Yang [73]	2013	empiric	X	X	X	X			X			5
Zheng [74]	2013	empiric	X	X								2
Anderson [75]	2014	empiric	X	X								2
Bloos [76]	2014	empiric								X	therapy considered inappropriate when antibiotics were changed/escalated	1
Chusri [77]	2014	empiric	X									1
Corrêa [78]	2014	empiric	X									1
De Bus [79]	2014	empiric	X									1
Esparcia [80]	2014	empiric	X									1
Falcone [81]	2014	empiric	X									1
Fayad [82]	2014	other	X				X	X				3

Garnacho-Montero [83]	2014	empiric	X	X	X							3
Girometti [84]	2014	empiric	X							X	specific antibiotics considered (in)appropriate in specific resistance pattern	2
Gonçalves-Pereira [85]	2014	empiric	X	X								2
Hsu [86]	2014	empiric	X	X								2
Jeong [87]	2014	empiric	X									1
Kim [88]	2014	empiric	X		X				X			3
		definite	X		X				X			3
Lee [89]	2014	empiric	X	X					X			3
Lipsky [90]	2014	empiric	X	X						X	rules for appropriateness when susceptibility testing was not performed/reported	3
Membrilla-Fernández [91]	2014	empiric	X									1
Nygård [92]	2014	empiric						X				1
Pelegrín [94]	2014	empiric				X				X	specific antibiotics that were considered (in)appropriate for the entire study cohort	2
Shorr [95]	2014	empiric	X	X			X					3
Spoorenberg [96]	2014	overall						X		X	- Diagnostic requirements<br>- De-escalation of antibiotics<br>- Switch to oral therapy	4
Vallés [97]	2014	empiric	X	X								2
Van Aken [98]	2014	empiric	X	X	X							3
Vilella [99]	2014	empiric	X									1
Yokota [100]	2014	empiric	X									1
Zeng [101]	2014	definite	X									1
Zilberberg [102]	2014	empiric	X	X			X					3
Al-Dorzi [103]	2015	empiric	X									1
Allou [104]	2015	empiric	X									1
Andria [105]	2015	empiric	X	X								2
Bass [106]	2015	unclear	X		X					X	consideration of pharmacokinetic parameters	3

Bastug [107]	2015	empiric	X	X								2
Beuving [108]	2015	unclear	X							X	unneccessarily broad therapy considered inappropriate	2
Boel [109]	2015	empiric	X									1
Brigmon [110]	2015	empiric	X		X	X						3
Coccolini [111]	2015	empiric								X	therapy considered appropriate based on clinical success	1
Denis [112]	2015	empiric	X	X	X							3
Dimopoulos [113]	2015	empiric	X									1
Inchai [114]	2015	empiric	X									1
Katsiari [115]	2015	empiric	X									1
Lee [116]	2015	empiric	X	X						X	monotherapy with specific antibiotics other than aminoglycosides considered inappropriate	3
Martin-Loeches [117]	2015	empiric	X									1
Martin-Loeches [118]	2015	empiric	X									1
Oliveira [119]	2015	empiric	X	X	X							3
Park [120]	2015	empiric	X	X								2
Rabello [121]	2015	empiric	X									1
Ratzinger [122]	2015	empiric	X									1
Shindo [123]	2015	empiric	X									1
Su [124]	2015	empiric	X		X		X					3
Suberviola Cañas [125]	2015	unclear	X									1
Sumida [126]	2015	unclear	X									1
Torres [127]	2015	empiric	X					X				2
Tumbarello [128]	2015	empiric	X									1
Wu [129]	2015	empiric	X	X	X	X						4
Abraham [130]	2016	empiric	X	X	X	X						4
Ali [131]	2016	empiric	X	X								2
Cheng [132]	2016	empiric	X									1

		definite	X									1
Chin [133]	2016	empiric	X							X	rules for appropriateness when susceptibility testing was not performed/reported	2
Cuervo [135]	2016	empiric	X						X			2
De Rosa [136]	2016	definite								X	specific antibiotics that were considered (in)appropriate for the entire study cohort	1
Fitzpatrick [137]	2016	empiric	X	X		X						3
Freire [138]	2016	unclear	X				X					2
Garnacho-Montero [139]	2016	empiric	X									1
Gonzalez [140]	2016	empiric	X									1
Guilbart [141]	2016	empiric	X									1
Guillamet [142]	2016	empiric	X									1
Herkel [143]	2016	empiric	X									1
Li [144]	2016	empiric	X									1
Li [145]	2016	empiric	X									1
Maeda [146]	2016	definite	X							X	therapy considered appropriate based on clinical success	2
Oshima [147]	2016	empiric	X									1
Palacios-Baena [148]	2016	empiric	X									1
		definite	X									1
Ruangchan [149]	2016	empiric	X									1
Savage [150]	2016	empiric	X									1
Stoma [151]	2016	empiric	X	X	X							3
Trecarichi [152]	2016	empiric	X									1
Vallés [153]	2016	empiric	X									1
Worapratya [154]	2016	empiric	X									1
Yilmaz [155]	2016	empiric								X	specific antibiotics that were considered (in)appropriate for the entire study cohort	1
Yoon [156]	2016	empiric	X	X	X	X						4
Zarco-Márquez [157]	2016	empiric	X	X			X					3

Zilberberg [158]	2016	empiric	X	X								2
Ahn [159]	2017	empiric	X									1
Babich [160]	2017	empiric	X	X								2
Battle [161]	2017	empiric	X		X	X						3
Bosch-Nicolau [162]	2017	empiric	X									1
Costa-de-Oliveira [163]	2017	empiric	X									1
Deconinck [164]	2017	empiric	X									1
González-Del Castillo [165]	2017	empiric								X	therapy considered inappropriate when antibiotics were changed/escalated	1
Goto [166]	2017	definite				X				X	specific antibiotics that were considered (in)appropriate for the entire study cohort	2
Gutiérrez-Gutiérrez [167]	2017	unclear	X	X								2
Jokinen [168]	2017	empiric	X									1
Joo [169]	2017	empiric	X	X	X	X						4
Li [171]	2017	empiric	X		X	X						3
	2017	definite	X		X	X						3
Micozzi [172]	2017	empiric	X									1
		definite	X									1
Póvoa [173]	2017	empiric	X									1
Palacios-Baena [174]	2017	empiric	X	X								2
		definite	X	X								2
Papadimitriou-Olivgeris [175]	2017	empiric	X	X	X							3
Pouwels [176]	2017	empiric	X	X								2
Rello [177]	2017	unclear						X				1
Royo-Cebrecos [178]	2017	empiric	X									1
Tagashira [179]	2017	empiric	X							X	specific antibiotics considered (in)appropriate in specific resistance pattern	2

Thaden [180]	2017	unclear	X									1
Tuon [181]	2017	empiric	X		X							2
		definite	X		X							2
Wang [182]	2017	empiric	X	X								2
Zhang [183]	2017	empiric	X		X							2
Zilberberg [184]	2017	empiric	X	X								2
Abdulsalam [185]	2018	empiric	X									1
Bassetti [186]	2018	empiric	X		X							2
Bouiller [187]	2018	general	X									1
		empiric	X									1
		definite	X									1
Chen [188]	2018	empiric	X	X	X	X						4
Claeys [189]	2018	empiric	X		X			X				3
		definite	X		X			X				3
Dewi [190]	2018	unclear			X			X		X	general rules for choice of empiric antibiotic	3
Fouks [191]	2018	empiric	X									1
Garcia-Vidal [192]	2018	empiric	X	X	X	X						4
Garrouste-Orgeas [193]	2018	empiric	X	X								2
Holmes [194]	2018	empiric	X									1
Islas-Muñoz [195]	2018	empiric	X	X			X					3
Kethireddy [196]	2018	empiric	X							X	rules for appropriateness when susceptibility testing was not performed/reported	2
Lee [197]	2018	empiric	X	X	X	X	X		X			6
Li [198]	2018	empiric	X									1
Papadopoulos [199]	2018	empiric	X									1
Saliba [200]	2018	empiric	X									1
Seas [201]	2018	definite				X				X	specific antibiotics that were considered (in)appropriate for the entire study cohort	2
Sommer [202]	2018	empiric	X	X								2
Tang [203]	2018	empiric	X									1

Tschudin-Sutter [204]	2018	empiric	X									1
Xu [205]	2018	empiric	X							X	therapy considered appropriate based on clinical success	2
Yamaga [206]	2018	empiric	X	X								2
Battle [207]	2019	empiric	X		X	X						3
Ben-Chetrit [209]	2019	empiric	X							X	specific antibiotics considered (in)appropriate in specific resistance pattern	2
Ben-Zvi [210]	2019	empiric								X	specific antibiotics that were considered (in)appropriate for the entire study cohort	1
Brescini [211]	2019	empiric	X									1
	2019	definite	X	X								2
Callejas-Díaz [212]	2019	empiric	X	X					X			3
	2019	definite	X						X			2
Castaño [213]	2019	empiric	X									1
Chusri [214]	2019	empiric	X									1
Delle Rose [215]	2019	empiric	X		X	X			X			4
Eliakim-Raz [216]	2019	empiric	X									1
Gómez Belda [217]	2019	empiric	X									1
Huang [218]	2019	definite	X				X					2
Jeon [219]	2019	empiric	X									1
Kim [220]	2019	empiric	X									1
Lat [221]	2019	empiric	X									1
Lim [222]	2019	empiric	X							X	specific antibiotics that were considered (in)appropriate for the entire study cohort	2
	2019	definite	X							X	specific antibiotics that were considered (in)appropriate for the entire study cohort	2
Maruyama [223]	2019	empiric	X									1
Morvan [224]	2019	empiric	X							X	rules for appropriateness when susceptibility testing was not performed/reported	2
Park [225]	2019	empiric	X									1

Ramos-Rincón [226]	2019	empiric	X									1
Rodriguez-Gómez [227]	2019	empiric	X							X	specific antibiotics that were considered (in)appropriate for the entire study cohort	2
Schuttevaer [228]	2019	empiric	X		X	X						3
Shi [229]	2019	empiric	X						X			2
	2019	definite	X						X			2
Tacconelli [230]	2019	empiric	X									1
Wang [231]	2019	definite	X									1
Wiggers [232]	2019	empiric	X	X								2
Al-Sunaidar [233]	2020	empiric	X	X	X			X		X	- De-escalation of antibiotics<br>- general rules for choice of empiric antibiotic	6
Augustin [234]	2020	empiric	X									1
Babich [235]	2020	empiric	X	X								2
Benetazzo [236]	2020	empiric	X	X								2
Chen [237]	2020	empiric	X	X								2
Chen [238]	2020	empiric	X									1
Dubler [239]	2020	empiric	X			X						2
Falcone [240]	2020	unclear	X									2
Ingram [241]	2020	other			X	X	X	X				4
Kang [242]	2020	empiric	X	X	X	X			X			5
Kawasuji [243]	2020	empiric	X									1
Kim [244]	2020	empiric	X					X				2
Lambregts, Wijnakker [245]	2020	empiric	X	X								2
Lee [246]	2020	empiric	X		X	X						3
Lee [247]	2020	empiric	X	X	X	X		X				5
Malbasa [248]	2020	empiric	X	X								2
Martinez-Nadal [249]	2020	empiric	X									1
Mitsuboshi [250]	2020	empiric	X									1
		definite	X									1

Montero [251]	2020	empiric	X	X								2
Mora-Guzmán [252]	2020	empiric	X				X					2
		definite	X				X					2
Papadimitriou-Olivgeris [253]	2020	empiric	X	X	X							3
Rhee [254]	2020	empiric	X									1
Righolt [255]	2020	empiric	X									1
Rivera-Espinar [256]	2020	empiric	X							X	therapy considered appropriate based on clinical success	2
		definite	X									1
Santos [257]	2020	empiric	X									1
Seo [258]	2020	empiric	X				X					2
		definite	X				X					2
Seok [259]	2020	empiric	X							X	- Expert opinion<br>- therapy considered appropriate based on clinical success	3
Wagner [260]	2020	empiric						X				1
Wang [261]	2020	empiric	X	X	X	X			X			5
Wiener-Well [262]	2020	empiric	X									1
Xiao, Zhu [263]	2020	empiric	X									1
		definite	X									1
Zhao [264]	2020	empiric	X	X								2
Aliyu [265]	2021	empiric	X									1
Amipara [266]	2021	empiric	X		X	X						3
Cetin [267]	2021	empiric	X									1
Chang [268]	2021	empiric	X									1
		definite	X									1
D'Onofrio [269]	2021	empiric						X				1
Gómez-Zorrilla [270]	2021	empiric	X									1
Jovanovic [271]	2021	empiric	X	X	X							3

Kadri [272]	2021	empiric	X									1
Kohler [273]	2021	empiric	X	X						X	specific antibiotics considered (in)appropriate in specific resistance pattern	3
Liu [274]	2021	empiric	X									1
	2021	definite	X									1
Man [275]	2021	empiric	X	X								2
Meng [276]	2021	empiric	X	X	X	X						4
	2021	definite	X	X	X	X						4
Moschou [277]	2021	empiric	X									1
Puzniak [278]	2021	empiric	X	X								2
Quillici [279]	2021	empiric	X	X								2
Rodríguez [280]	2021	empiric	X							X	rules regarding combination therapy	2
	2021	definite	X							X	rules regarding combination therapy	2
Shen [281]	2021	empiric	X	X								2
	2021	definite	X									1
Shorr [282]	2021	empiric	X							X	specific antibiotics that were considered (in)appropriate for the entire study cohort	2
Sun, Zhao [283]	2021	empiric	X									1
Teelucksingh [284]	2021	empiric	X	X						X	specific antibiotics that were considered (in)appropriate for the entire study cohort	3
Thy [285]	2021	empiric	X									1
Yiang [286]	2021	empiric	X									1
Zhang [287]	2021	empiric	X							X	therapy considered appropriate based on clinical success	2
Zhu, Chen [288]	2021	empiric	X									1
Zilberberg [289]	2021	empiric	X	X								2

S Table 4: Definitions of the terms "empiric" and "definite" therapy reported in the studies

While 89/258 (34.50%) of studies providing a definition of empiric AAT defined what "empiric" referred to in their respective study context, this was true for 26/41 (63.41%) studies investigating definite AAT. 118/258 (45.74%) studies defined a timeframe that was considered to be empiric, 11/41 (26.83%) a timeframe for definite therapy.

Category	Treatment Phase	Aspects used in respective definitions		Number of studies (%)
Definition	Empiric	Antibiotic regimen prescribed before availability of culture and/or in vitro susceptibility test results		47 (52.81%)
		Antibiotic regimen started within specific time after microbiological culture collection		21 (23.60%)
		First antibiotic regimen prescribed/antibiotics administered during first day(s)		14 (15.73%)
		Antibiotic regimen started within specific time after infection onset		14 (15.73%)
		Other		4 (4.49%)
	Definite	Antibiotic regimen started/changed to after pathogen identification and/or availability of susceptibility testing		23 (88.46%)
		Other		4 (15.38%)
Timing	Empiric	Timeframe	<24 hours	3 (2.54%)
			24 hours/same day	63 (53.39%)
			48 hours	41 (34.75%)
			72 hours	9 (7.63%)
			5 days	2 (1.69%)
		Timepoint	After microbiological culture collection	55 (46.61%)
			After infection onset/diagnosis	40 (33.90%)
			Hospital/ICU admission	9 (7.63%)
			Unspecific/Other	14 (11.86%)
	Definite	Timeframe	24 hours	3 (27.27%)
			48 – 72 hours	3 (27.27%)
			4 – 5 days	3 (27.27%)
			7 days	2 (18.18%)
		Timepoint	After microbiological culture collection	6 (54.55%)
			After pathogen identification/susceptibility testing	4 (36.36%)
			Unspecific	1 (9.09%)

S Table 5: Overview of aspects included in study definitions of appropriate antibiotic therapy (AAT) for unclear and other treatment phase

Aspect	Number of definitions	definitions with details on evaluation
**Total number of definitions of unclear AAT**	14	
Susceptibility	12 (85.71%)	8 (66.67%)
Timing	1 (7.14%)	1 (100.00%)
Dosing	2 (14.29%)	1 (50.00%)
Route of administration	-	-
Aminoglycoside restriction	1 (7.14%)	1 (100.00%)
Duration	3 (21.43%)	3 (100.00%)
Guideline-based choice of antibiotic agent	2 (14.29%)	1 (50.00%)
Other	5 (35.71%)	5 (100.00%)
**Total number of definitions of other AAT**	3	
Susceptibility	2 (66.67%)	1 (50.00%)
Timing	-	-
Dosing	1 (33.33%)	1 (100.00%)
Route of administration	1 (33.33%)	1 (100.00%)
Aminoglycoside restriction	-	-
Duration	2 (66.67%)	2 (100.00%)
Guideline-based choice of antibiotic agent	2 (66.67%)	2 (100.00%)
Other	-	-

S Table 6: Detailed methods for evaluation of appropriate antibiotic therapy (AAT) aspects in study definitions of empiric therapy (n=258)

Aspect	Subaspects	Number of definitions	Number of definitions with details on evaluation
**Total number of definitions of empiric AAT**		258 (100.00%)	
**Susceptibility**		245 (94.96%)	151 (61.63%)
	Clinical breakpoints		
	Clinical and Laboratory Standards Institute		109 (72.19%)
	European Committee on Antimicrobial Susceptibility Testing		26 (17.22%)
	French Society for Microbiology		12 (7.95%)
	Mixed		4 (2.65%)
**Timing**		92 (35.66%)	91 (98.91%)
	Reference point for timely initiation		
	Microbiological culture collection		47 (51.65%)
	Symptom onset / diagnosis of infection		28 (30.77%)
	Hospital admission / ED presentation		7 (7.69%)
	Specific timeframe without reference point		5 (5.49%)
	Other / Mixed		4 (4.40%)
	Time until antibiotic had to be started		
	Within 6h		3 (3.27%)
	Within 12h		1 (1.10%)
	Within 24h / same day		50 (54.95%)
	Within 48h		29 (31.87%)
	Within 72h		6 (6.59%)
	Other		1 (1.10%)
**Dosing**		49 (18.99%)	21 (42.86%)
	Assessment of dosing*		
	Appropriate for endorgan function		8 (38.10%)
	According to guideline		7 (33.33%)
	Adaption to renal function when required		6 (28.57%)
	Appropriate dosing interval		4 (19.05%)
	Other		3 (14.29%)
**Route of Administration**		34 (13.18%)	14 (41.18%)
	Assessment of route of administration*		
	Only i.v. therapy considered appropriate		7 (50.00%)
	According to guideline		3 (21.43%)
	Only i.v. therapy considered appropriate with exceptions for antibiotics with good bioavailability		3 (21.43%)
	Other		1 (7.14%)
**Aminoglycoside restriction**		16 (6.20%)	16 (100.00%)
	Assessment of aminoglycoside use*		
	Use of aminoglycosides as monotherapy was not considered adequate		12 (75.00%)
	Any aminoglycoside use considered inappropriate		2 (12.50%)
	Other aspects		3 (18.75%)
**Duration**		11 (4.26%)	10 (90.91%)
	Minimum duration of therapy required		
	24 hours		6 (60.00%)
	48 hours		1 (10.00%)
	72 hours		3 (30.00%)
		11 (4.26%)	11 (100.00%)

**Guideline-based choice of antibiotic agent**	Applied guideline<br>Local guideline<br>Non-local guideline<br>Local or non-local guideline		 5 (45.45%)<br>4 (36.36%)<br>2 (18.18%)
**Other**[a]		33 (12.79%)	33 (100.00%)
	Specific antibiotics considered (in)appropriate in specific resistance pattern		8 (24.24%)
	Rules for appropriateness when susceptibility testing was not performed/reported		5 (15.15%)
	Specific antibiotics considered appropriate for the entire study cohort		5 (15.15%)
	Therapy considered appropriate based on clinical success		5 (15.15%)
	Specific antibiotics considered inappropriate for the entire study cohort		3 (9.09%)
	Other		8 (24.24%)

a. One study may use multiple aspects of assessment.

S Table 7: Detailed methods for evaluation of appropriate antibiotic therapy (AAT) aspects in study definitions of definite therapy (n=41)

Aspect	Subaspects	Number of definitions	Number of definitions with details on evaluation
**Total number of definitions of definite AAT**		41 (100%)	
**Susceptibility**		36 (87.81%)	31 (86.11%)
	Clinical breakpoints<br>Clinical and Laboratory Standards Institute<br>European Committee on Antimicrobial Susceptibility Testing<br>Mixed		 20 (64.52%)<br>7 (22.58%)<br>4 (12.90%)
**Timing**		4 (9.76%)	4 (100.00%)
	Reference point for timely initiation<br>Microbiological culture collection<br>Symptom onset / diagnosis of infection<br>Other / Mixed		 1 (25.00%)<br>1 (25.00%)<br>2 (50.00%)
	Time until antibiotic has to be started<br>Within 24h<br>Within 96h<br>Within 120h		 2 (50.00%)<br>1 (25.00%)<br>1 (25.00%)
**Dosing**		10 (24.39%)	7 (70.00%)
	Assessment of dosing*<br>Appropriate for endorgan function<br>According to guideline<br>Adaption to renal function when required<br>Appropriate dosing interval<br>Other		 1 (14.29%)<br>4 (57.14%)<br>2 (28.57%)<br>1 (14.29%)<br>1 (14.29%)
**Route of Administration**		5 (12.20%)	2 (40.00%)
	Assessment of route of administration*<br>Only i.v. therapy considered appropriate		 2 (100.00%)
**Aminoglycoside restriction**		5 (12.20%)	5 (100.00%)
	Use of aminoglycosides as monotherapy was not considered adequate		5 (100.00%)
**Duration**		6 (14.63%)	6 (100.00%)
	Assessment of duration of therapy<br>Minimum duration of therapy required<br>According to local guideline		 5 (83.33%)<br>1 (16.67%)
**Guideline-based choice of antibiotic agent**		2 (4.88%)	2 (100.00%)
	Applied guideline<br>Local guideline<br>Local or non-local guideline		 1 (50.00%)<br>1 (50.00%)
**Other**[a]		9 (21.95%)	9 (100.00%)
	Specific antibiotics considered appropriate for the entire study cohort<br>Therapy considered appropriate based on clinical success<br>Specific antibiotics considered inappropriate for the entire study cohort<br>Other		4 (44.44%)<br>1 (11.11%)<br>1 (11.11%)<br>3 (33.33%)

a. One study may use multiple aspects of assessment.

S Table 8: Assessment of appropriate antibiotic therapy (AAT) in patients without pathogen identification among studies including such patients and providing information on AAT assessment

Of 62 studies including patients without pathogen identification, 61 studies included both microbiologically confirmed and non-confirmed infections, while 1 study included culture-negative infections only. 55/62 studies (88.71%) studies provided the number of isolated pathogens, with a mean percentage (IQR) of isolated pathogens of 62.9% (45;78.8). Assessment of AAT in studies without pathogen identification is presented in the table below.

**Assessment of appropriateness**	**n**	**%**
No assessment of appropriateness	24	50.00
Study definition of AAT applies to both culture-negative and culture-positive patients	11	22.92
Guideline-based assessment of appropriateness	7	14.58
Expert assessment	3	6.25
Treatment considered appropriate for all culture-negative patients	3	6.25
Other	3	6.25

S Table 9: Assessment of AMS objectives and individual appropriate antibiotic therapy (AAT) aspects for their effect on patient outcomes

115/284 (40.49%) studies providing definition of AAT assessed the effect of AMS objectives and/or individual aspects of their respective AAT definition on outcomes.

Aspects of AMS assessed in the corresponding studies	n	%
Combination therapy	52	44.07
Source control	29	25.22
Time to AAT	28	24.35
Timely administration of antibiotics	19	16.52
Duration of antibiotic therapy	16	13.91
De-escalation or streamlining	9	7.83
Appropriate diagnostics	8	6.97
Infectious disease consultation	7	6.09
Change of empiric therapy	6	5.22
Dosing	5	4.35
Process-related aspects	4	3.48
Susceptibility	3	2.61
Guideline-based therapy	3	2.61
Intravenous to oral switch	2	1.74
Use of specific antibiotics	2	1.74
AMS intervention	2	1.74
Route of administration	2	1.74
Documentation related to antibiotic therapy	1	0.87
Other	7	6.09

S Table 10: Sources and other ways of determining study definitions of appropriate antibiotic therapy (AAT)

77/288 (26.74%) studies specified how the AAT definition was developed

Development of the AAT definition was based on	n	%
Adoption of definition from previous study	68	87.01
Guideline	12	14.47
Consensus procedure	2	2.63
Expert opinion	2	2.63

S Table 11: Mentioned strengths and limitations of appropriate antibiotic therapy (AAT) definitions among studies providing critical assessment of their respective study definitions

15/288 (5.21%) studies commented on the strengths of their definition, 61/288 (21.18%) commented on limitations

**Strengths mentioned**	**n**	**%**
Definition in accordance with previous publication(s)	5	33.33
Definition of AAT very comprehensive	3	20.00
Definition clear and/or precise	2	13.33
Study results support that the definition of AAT is well chosen	2	13.33
Other	3	20.00
**Limitations mentioned**	**n**	**%**
Aspects that were not evaluated as part of the definition	48	78.69
Dosing (including therapeutic drug monitoring and dosing interval)	16	26.23
Timing	7	11.48
Pharmacodynamics and pharmacokinetics (including tissue penetration and interactions)	6	9.84
Route of administration	4	6.56
Duration	3	4.92
Indication of antibiotic treatment	2	3.28
Aminoglycoside restriction	2	3.28
Other	4	6.56
Generally, more aspects of AAT should have been evaluated	4	6.56
In vitro sensitivity does not equal in vivo effectiveness or treatment of choice	8	13.11
Breakpoint-related limitations	7	11.48
Breakpoints used in the study not up-to-date	4	6.56
Breakpoints vary between agencies	3	4.92
Appropriateness of empirical or definite therapy was not evaluated	3	4.92
Lack of standard definition of AAT to base the study definition upon	3	4.92
Susceptibility-based definition does not sanction overtreatment or promotes unnecessary use of broad-spectrum antibiotics	3	4.92
Tigecycline was considered appropriate in BSI	2	3.28
Different definitions for culture-positive and culture-negative patients	2	3.28
Patients without positive cultures were not evaluated for appropriateness	2	3.28
Other	21	34.43

S Table 12: Study characteristics and definitions of appropriate antibiotic therapy (AAT) grouped according to use of susceptibility and other aspects for empiric therapy (n=258)

	Grouping of AAT-definition					
	susceptibility only	Susceptibility + 1 other aspect	Susceptibility + timing	Susceptibility + ≥ 2 other aspects	Non-susceptibility-centered	All definitions of empirical AAT
**Study characteristic**	n (%)	n (%)	n (%)	n (%)	n (%)	n (%)
**Overall**	105 (40.70%)	76 (29.46%)	46 (17.83%)	64 (24.81%)	13 (5.04%)	258 (100.00%)
Year of publication						
2011 – 2014	28 (26.67%)	28 (36.84%)	20 (43.48%)	28 (43.75%)	5 (38.46%)	89 (34.50%)
2015 – 2018	56 (53.33%)	28 (36.84%)	14 (30.43%)	24 (37.50%)	5 (38.46%)	113 (43.80%)
2019 – 2021	21 (20.00%)	20 (26.32%)	12 (26.09%)	12 (18.75%)	3 (23.08%)	56 (21.71%)
Geographical Distribution						
Europe	49 (46.67%)	30 (39.47%)	17 (36.96%)	19 (29.69%)	8 (61.54%)	106 (41.09%)
Asia	37 (35.24%)	26 (34.21%)	14 (30.43%)	26 (40.62%)	2 (15.38%)	91 (35.27%)
North America	12 (11.43%)	13 (17.11%)	11 (23.91%)	16 (25.00%)	1 (7.69%)	42 (16.28%)
South America	5 (4.76%)	2 (2.63%)	1 (2.17%)	3 (4.69%)	1 (7.69%)	11 (4.26%)
Oceania	1 (0.95%)	1 (1.32%)	-	-	-	2 (0.78%)
Intercontinental	1 (0.95%)	4 (5.26%)	3 (6.52%)	-	1 (7.69%)	6 (2.33%)
Hospital type						
Secondary Care	3 (2.86%)	2 (2.63%)	1 (2.17%)	4 (6.25%)	-	9 (3.59%)
Tertiary Care	78 (74.29%)	47 (61.84%)	27 (58.70%)	45 (70.31%)	10 (76.92%)	180 (69.77%)
Mixed	7 (6.67%)	11 (14.47%)	9 (19.57%)	7 (10.94%)	1 (7.69%)	26 (10.08%)
Unclear	17 (16.19%)	16 (21.05%)	9 (19.57%)	8 (12.50%)	2 (15.38%)	43 (16.67%)
Study department						
Hospital wide	55 (52.38%)	51 (67.11%)	28 (60.87%)	48 (75.00%)	5 (38.46%)	159 (61.63%)
ICU	33 (31.43%)	18 (23.68%)	13 (28.26%)	11 (17.19%)	1 (7.69%)	63 (24.42%)
Emergency department	6 (5.71%)	2 (2.63%)	1 (2.17%)	4 (6.25%)	2 (15.38%)	14 (5.43%)
Mixed	11 (10.48%)	5 (6.58%)	4 (8.70%)	-	4 (30.77%)	20 (7.75%)
Unclear	-	-	-	1 (1.56%)	1 (7.69%)	2 (0.78%)
Target pathogens						
P. aeruginosa	5 (4.76%)	8 (10.53%)	6 (13.04%)	7 (10.94%)	-	20 (7.75%)
K. pneumoniae	7 (6.67%)	8 (10.53%)	3 (6.52%)	2 (3.12%)	-	17 (6.59%)
S. aureus	5 (4.76%)	5 (6.58%)	2 (4.35%)	3 (4.69%)	2 (15.38%)	15 (5.81%)
A. baumannii	2 (1.90%)	4 (5.26%)	3 (6.52%)	6 (9.38%)	-	12 (4.65%)
E. coli	1 (0.95%)	2 (2.63%)	1 (2.17%)	3 (4.69%)	-	6 (2.33%)

S. pneumoniae	-	2 (2.63%)	1 (2.17%)	1 (1.56%)	-	3 (1.16%)
Mixed	19 (18.10%)	23 (30.26%)	13 (28.26%)	22 (34.38%)	-	64 (24.81%)
Other	-	1 (1.32%)	-	2 (3.12%)	2 (15.38%)	5 (1.94%)
Unrestricted by pathogen	66 (62.86%)	23 (30.26%)	17 (36.96%)	18 (28.12%)	9 (69.23%)	116 (44.96%)
Resistance mechanism of target pathogens						
Carbapenem-resistance	7 (6.67%)	6 (7.89%)	2 (4.35%)	1 (1.56%)	-	14 (5.43%)
ESBL-production	7 (6.67%)	5 (6.58%)	4 (8.70%)	2 (3.12%)	-	14 (5.43%)
MRSA	1 (0.95%)	2 (2.63%)	-	2 (3.12%)	-	5 (1.94%)
VRE	1 (0.95%)	-	-	-	-	1 (0.39%)
Unspecified	-	1 (1.32%)	1 (2.17%)	1 (1.56%)	-	2 (0.78%)
Unrestricted by resistance	89 (84.76%)	62 (81.58%)	139 (84.78%)	58 (90.62%)	13 (100.00%)	222 (86.05%)
Target type of infection						
Bloodstream infection	59 (56.19%)	47 (61.84%)	28 (60.87%)	54 (84.38%)	6 (46.15%)	166 (64.34%)
Respiratory infection	24 (22.86%)	15 (19.74%)	8 (17.39%)	3 (4.69%)	2 (15.38%)	44 (17.05%)
Urinary tract infection	7 (6.67%)	3 (3.95%)	2 (4.35%)	-	-	10 (3.88%)
Abdominal infection	4 (3.81%)	2 (2.63%)	-	-	2 (15.38%)	8 (3.10%)
Central nervous system	1 (0.95%)	-	-	-	1 (7.69%)	2 (0.78%)
Skin and soft tissue	1 (0.95%)	1 (1.32%)	1 (2.17%)	2 (3.12%)	-	4 (1.55%)
Mixed / Other	3 (2.86%)	4 (5.26%)	4 (8.70%)	-	-	7 (2.71%)
Unrestricted by type of infection	6 (5.71%)	4 (5.26%)	3 (6.52%)	5 (7.81%)	2 (15.38%)	17 (6.59%)
Target site of acquisition						
Healthcare-setting	24 (22.86%)	15 (19.74%)	8 (17.39%)	11 (17.19%)	-	50 (19.38%)
Community acquired	17 (16.19%)	11 (14.47%)	7 (15.22%)	10 (15.62%)	7 (53.85%)	45 (17.44%)
Unrestricted by acquisition	64 (60.95%)	50 (65.79%)	31 (67.39%)	43 (67.19%)	6 (46.15%)	163 (63.18%)
Immunosuppressed and cancer patients	8 (7.62%)	6 (7.89%)	3 (6.52%)	7 (10.94%)	-	21 (8.14%)
Appropriateness in study objectives						
AAT mentioned in study objectives	35 (33.33%)	31 (40.79%)	21 (45.65%)	25 (39.06%)	4 (30.77%)	95 (36.82%)
Impact of AAT on outcomes in study objectives	27 (25.71%)	25 (32.89%)	16 (34.78%)	22 (34.38%)	3 (23.08%)	77 (29.84%)
Source of definition specified in the paper	23 (21.90%)	20 (26.32%)	12 (26.09%)	21 (32.81%)	2 (15.38%)	66 (25.58%)
Reflection of quality/limitations of the definition in the paper	18 (17.14%)	18 (24.32%)	11 (23.91%)	16 (25.00%)	2 (15.38%)	54 (21.09%)

S Table 13: Study characteristics and definitions of appropriate antibiotic therapy (AAT) grouped according to use of susceptibility and other aspects for definite therapy (n=41)

	Grouping of AAT-definition					
	susceptibility only	Susceptibility + 1 other aspect	Susceptibility + timing	Susceptibility + ≥ 2 other aspects	Non-susceptibility-centered	All definitions of definite AAT
**Study characteristic**	n (%)	n (%)	n (%)	n (%)	n (%)	n (%)
**Overall**	13 (31.71%)	16 (39.02%)	2 (4.88%)	7 (17.07%)	5 (12.20%)	41 (100.00%)
Year of publication						
2011 – 2014	2 (15.38%)	5 (31.25%)	-	4 (57.14%)	2 (40.00%)	13 (31.71%)
2015 – 2018	5 (38.46%)	8 (50.00%)	2 (100.00%)	2 (28.57%)	3 (60.00%)	18 (43.90%)
2019 – 2021	6 (46.15%)	3 (18.75%)	-	1 (14.29%)	-	10 (24.39%)
Geographical Distribution						
Europe	4 (30.77%)	6 (37.50%)	1 (50.00%)	2 (28.57%)	2 (40.00%)	14 (34.15%)
Asia	9 (69.23%)	7 (43.75%)	-	4 (57.14%)	-	20 (48.78%)
North America	-	-	-	1 (14.29%)	2 (40.00%)	3 (7.32%)
South America	-	1 (6.25%)	-	-	1 (20.00%)	2 (4.88%)
Oceania	-	1 (6.25%)	-	-	-	1 (2.44%)
Intercontinental	-	1 (6.25%)	1 (50.00%)	-	-	1 (2.44%)
Hospital type						
Secondary Care	1 (7.69%)	1 (6.25%)	-	-	-	2 (4.88%)
Tertiary Care	10 (76.92%)	14 (87.50%)	2 (100.00%)	5 (71.43%)	1 (20.00%)	30 (73.17%)
Mixed	1 (7.69%)	1 (6.25%)	-	1 (14.29%)	-	3 (7.32%)
Unclear	1 (7.69%)	-	-	1 (14.29%)	4 (80.00%)	6 (14.63%)
Study department						
Hospital wide	10 (76.92%)	15 (93.75%)	2 (100.00%)	5 (71.43%)	5 (100.00%)	35 (85.37%)
ICU	2 (15.38%)	-	-	2 (28.57%)	-	4 (9.76%)
Mixed	1 (7.69%)	1 (6.25%)	-	-	-	2 (4.88%)
Target pathogens						
P. aeruginosa	-	3 (18.75%)	-	2 (28.57%)	-	5 (12.50%)
K. pneumoniae	5 (38.46%)	4 (25.00%)	1 (50.00%)	1 (14.29%)	-	10 (24.39%)
S. aureus	1 (7.69%)	-	-	-	3 (60.00%)	4 (9.76%)
A. baumannii	-	-	-	3 (42.86%)	-	3 (7.32%)
Mixed	6 (46.15%)	8 (50.00%)	1 (50.00%)	1 (14.29%)	1 (20.00%)	16 (39.02%)
Other	-	-	-	-	1 (20.00%)	1 (2.44%)
Unrestricted by pathogen	1 (7.69%)	1 (6.25%)	-	-	-	2 (4.88%)

Resistance mechanism of target pathogens						
Carbapenem-resistance	5 (38.46%)	6 (37.50%)	1 (50.00%)	1 (14.29%)	-	12 (29.27%)
ESBL-production	3 (23.08%)	2 (12.50%)	1 (50.00%)	-	-	5 (12.20%)
VRE	-	-	-	-	1 (20.00%)	1 (2.44%)
Unrestricted by resistance	5 (38.46%)	8 (50.00%)	-	6 (85.71%)	4 (80.00%)	23 (56.10%)
Target type of infection						
Bloodstream infection	9 (69.23%)	14 (87.50%)	2 (100.00%)	4 (57.14%)	3 (60.00%)	30 (73.17%)
Respiratory infection	2 (15.38%)	1 (6.25%)	-	2 (28.57%)	1 (20.00%)	6 (14.63%)
Urinary tract infection	-	-	-	-	1 (20.00%)	1 (2.44%)
Abdominal infection	-	1 (6.25%)	-	-	-	1 (2.44%)
Mixed / Other	1 (7.69%)	-	-	-	-	1 (2.44%)
Unrestricted by type of infection	1 (7.69%)	-	-	1 (14.29%)	-	2 (4.88%)
Target site of acquisition						
Healthcare-setting	2 (15.38%)	3 (18.75%)	-	3 (42.86%)	-	8 (19.51%)
Community acquired	-	1 (6.25%)	-	-	1 (20.00%)	2 (4.88%)
Unrestricted by acquisition	11 (84.62%)	12 (75.00%)	2 (100.00%)	4 (57.14%)	4 (80.00%)	31 (75.61%)
Immunosuppressed and cancer patients	2 (15.38%)	-	-	-	-	2 (4.88%)
Appropriateness in study objectives						
AAT mentioned in study objectives	1 (7.69%)	4 (25.00%)	1 (50.00%)	4 (57.14%)	1 (20.00%)	10 (24.39%)
Impact of AAT on outcomes in study objectives	-	4 (25.00%)	1 (50.00%)	3 (42.86%)	-	7 (17.07%)
Source of definition specified in the paper	1 (7.69%)	4 (25.00%)	-	1 (14.29%)	2 (40.00%)	8 (19.51%)
Reflection of quality/limitations of the definition in the paper	1 (7.69%)	5 (31.25%)	1 (50.00%)	3 (42.86%)	1 (20.00%)	10 (24.39%)

S Table 14: Study characteristics and definitions of appropriate antibiotic therapy (AAT) grouped according to use of individual aspects for empiric therapy (n=258)

	Aspect of AAT								
	susceptibility	Timely start of therapy	dosing	Route of administration	duration	Guideline.based	Aminoglycoside restriction	other	Overall definition
**Study characteristic**	n (%)	n (%)	n (%)	n (%)	n (%)	n (%)	n (%)	n (%)	n (%)
**Overall**	n = 245	n = 92	n =49	n = 34	n =11	n = 12	n =16	n =32	n = 258
Year of publication									
2011 – 2014	84 (34.29%)	41 (44.57%)	18 (36.73%)	13 (38.24%)	5 (45.45%)	3 (25.00%)	9 (56.25%)	11 (34.38%)	89 (34.50%)
2015 – 2018	108 (44.08%)	30 (32.61%)	22 (44.90%)	14 (41.18%)	4 (36.35%)	3 (25.00%)	5 (31.25%)	13 (40.62%)	113 (43.80%)
2019 – 2021	53 (21.63%)	21 (22.83%)	9 (18.37%)	7 (20.59%)	2 (18.18%)	6 (50.00%)	2 (12.50%)	8 (25.00%)	56 (21.71%)
Geographical Distribution									
Europe	98 (40.00%)	30 (32.61%)	16 (32.65%)	9 (26.47%)	1 (9.09%)	5 (41.67%)	6 (37.50%)	13 (40.62%)	106 (41.09%)
Asia	89 (36.33%)	33 (35.87%)	24 (48.98%)	19 (55.88%)	3 (27.27%)	4 (33.33%)	10 (62.50%)	9 (28.12%)	91 (35.27%)
North America	41 (16.73%)	22 (23.91%)	6 (12.24%)	5 (14.71%)	6 (54.55%)	2 (16.67%)	-	6 (18.75%)	42 (16.28%)
South America	10 (4.08%)	4 (4.35%)	3 (6.12%)	1 (2.94%)	1 (9.09%)	1 (8.33%)	-	1 (3.12%)	11 (4.26%)
Oceania	2 (0.82%)	-	-	-	-	-	-	1 (3.12%)	2 (0.78%)
Intercontinental	5 (2.04%)	3 (3.26%)	-	-	-	-	-	2 (6.25%)	6 (2.33%)
Hospital type									
Secondary Care	9 (3.67%)	2 (2.17%)	4 (8.16%)	3 (8.82%)	-	-	-	1 (3.12%)	9 (3.59%)
Tertiary Care	170 (69.39%)	60 (65.22%)	34 (69.39%)	22 (64.71%)	10 (90.91%)	10 (83.33%)	9 (56.25%)	22 (68.75%)	180 (69.77%)
Mixed	25 (10.20%)	15 (16.30%)	5 (10.20%)	4 (11.76%)	-	-	2 (12.50%)	2 (6.25%)	26 (10.08%)
Unclear	41 (16.73%)	15 (16.30%)	6 (12.24%)	5 (14.71%)	1 (9.09%)	2 (16.67%)	5 (31.25%)	7 (21.88%)	43 (16.67%)
Study department									
Hospital wide	154 (62.86%)	62 (67.39%)	37 (75.51%)	28 (82.35%)	7 (63.64%)	3 (25.00%)	15 (93.75%)	22 (68.75%)	159 (61.63%)
ICU	62 (25.31%)	23 (25.00%)	8 (16.33%)	2 (5.88%)	2 (18.18%)	3 (25.00%)	1 (6.25%)	6 (18.75%)	63 (24.42%)
Emergency department	12 (4.90%)	2 (2.17%)	3 (6.12%)	3 (8.82%)	-	3 (25.00%)	-	2 (6.25%)	14 (5.43%)

Mixed	16 (6.53%)	4 (4.35%)	-	-	1 (9.09%)	3 (25.00%)	-	1 (3.12%)	20 (7.75%)
Unclear	1 (0.41%)	1 (1.09%)	1 (2.04%)	1 (2.94%)	1 (9.09%)	-	-	1 (3.12%)	2 (0.78%)
Target pathogens									
P. aeruginosa	20 (8.16%)	10 (10.87%)	3 (6.12%)	2 (5.88%)	1 (9.09%)	-	5 (31.25%)	2 (6.25%)	20 (7.75%)
K. pneumoniae	17 (6.94%)	4 (4.35%)	2 (4.08%)	1 (2.94%)	-	-	-	5 (15.62%)	17 (6.59%)
S. aureus	12 (5.31%)	5 (5.43%)	3 (6.12%)	2 (5.88%)	1 (9.09%)	-	2 (12.50%)	3 (9.38%)	15 (5.81%)
A. baumannii	12 (4.90%)	9 (9.78%)	5 (10.20%)	4 (11.76%)	-	-	3 (18.75%)	1 (3.12%)	12 (4.65%)
E. coli	6 (2.45%)	3 (3.26%)	3 (6.12%)	1 (2.94%)	-	-	-	1 (3.12%)	6 (2.33%)
S. pneumoniae	3 (1.22%)	2 (2.17%)	-	-	1 (9.09%)	-	-	1 (3.12%)	3 (1.16%)
Mixed	64 (26.12%)	28 (30.43%)	18 (36.73%)	12 (35.29%)	7 (63.64%)	2 (16.67%)	6 (37.50%)	6 (18.75%)	64 (24.81%)
Other	3 (1.22%)	2 (2.17%)	-	3 (8.82%)	-	1 (8.33%)	-	2 (6.25%)	5 (1.94%)
Unrestricted by pathogen	107 (43.67%)	29 (31.52%)	14 (28.57%)	9 (26.47%)	1 (9.09%)	9 (75.00%)	-	11 (34.38%)	116 (44.96%)
Resistance mechanism of target pathogens						-			
Carbapenem-resistance	14 (5.71%)	3 (3.26%)	3 (6.12%)	-	2 (18.18%)	-	-	-	14 (5.43%)
ESBL-production	14 (5.71%)	5 (5.43%)	2 (4.08%)	2 (5.88%)	-	-	-	1 (3.12%)	14 (5.43%)
MRSA	5 (2.04%)	2 (2.17%)	1 (2.04%)	1 (2.94%)	-	-	2 (12.50%)	1 (3.12%)	5 (1.94%)
VRE	1 (0.41%)	-	-	-	-	-	-	-	1 (0.39%)
Unspecified	2 (0.82%)	1 (1.09%)	1 (2.04%)	-	1 (9.09%	-	-	-	2 (0.78%
Unrestricted by resistance	209 (85.31%)	81 (88.04%)	42 (85.71%)	31 (91.18%)	8 (72.73%))	12 (100.00%)	14 (87.50%)	30 (93.75%)	222 (86.05%)
Target type of infection									
Bloodstream infection	160 (65.31%)	67 (72.83%)	43 (87.76%)	32 (94.12%)	9 (81.82%)	6 (50.00%)	15 (93.75%)	17 (53.12%)	166 (64.34%)
Respiratory infection	42 (17.14%)	9 (9.78%)	3 (6.12%)	-	-	4 (33.33%)	1 (6.25%)	6 (18.75%)	44 (17.05%)
Urinary tract infection	10 (4.08%)	2 (2.17%)	-	-	-	-	-	1 (3.12%)	10 (3.88%)
Abdominal infection	6 (2.45%)	-	-	-	1 (9.09%)	1 (8.33%)	-	2 (6.25%)	8 (3.10%)
Central nervous system	1 (0.41%)	-	-	1 (2.94%)	-	-	-	1 (3.12%)	2 (0.78%)
Skin and soft tissue	4 (1.63%)	3 (3.26%)	-	-	-	-	-	2 (6.25%)	4 (1.55%)
Mixed / Other	7 (2.86%)	4 (4.35%)	-	-	-	-	-	-	7 (2.71%)
Unrestricted by type of infection	15 (6.12%)	7 (7.61%)	3 (6.12%)	1 (2.94%)	1 (9.09%)	1 (8.33%)	-	3 (9.38%)	17 (6.59%)
Target site of acquisition									
Healthcare acquired	50 (20.41%)	15 (16.30%)	14 (28.57%)	7 (20.59%)	3 (27.27%)	-	4 (25.00%)	4 (12.50%)	50 (19.38%)
Community acquired	38 (15.51%)	13 (14.12%)	7 (14.29%)	6 (17.65%)	1 (9.09%)	7 (58.33%)	-	7 (21.88%)	45 (17.44%)

Unrestricted by acquisition	157 (64.08%)	64 (69.57%)	28 (57.14%)	21 (61.76%)	7 (63.64%)	5 (41.67%)	12 (75.00%)	21 (65.62%)	163 (63.18%)
Immunosuppressed and cancer patients	21 (8.57%)	9 (9.78%)	3 (6.12%)	3 (8.82%)	2 (18.18%)	-	-	5 (15.62%)	21 (8.14%)
Appropriateness in study objectives									
AAT mentioned in study objectives	91 (37.14%)	37 (40.22%)	20 (40.82%)	15 (44.12%)	2 (18.18%)	6 (50.00%)	3 (18.75%)	13 (40.62%)	95 (36.82%)
Impact of AAT on outcomes in study objectives	74 (30.20%)	31 (33.70%)	17 (34.69%)	14 (41.18%)	2 (18.18%)	3 (25.00%)	3 (18.75%)	11 (34.38%)	77 (29.84%)
Source of definition specified in the paper	64 (26.12%)	25 (27.17%)	17 (34.69%)	14 (41.18%)	2 (18.18%)	6 (50.00%)	1 (6.25%)	8 (25.00%)	66 (25.58%)
Reflection of quality/limitations of the definition in the paper	52 (21.40%)	23 (25.56%)	9 (18.37%)	4 (11.76%)	2 (18.18%)	2 (16.67%)	5 (31.25%)	9 (28.12%)	54 (21.09%)

S Table 15: Study characteristics and definitions of appropriate antibiotic therapy (AAT) grouped according to use of individual aspects for definite therapy (n=41)

	Aspect of AAT								
	susceptibility	Timely start of therapy	dosing	Route of administration	duration	Guideline.based	Aminoglycoside restriction	other	Overall definition
**Study characteristic**	n (%)	n (%)	n (%)	n (%)	n (%)	n (%)	n (%)	n (%)	n (%)
**Overall**	n = 36	n = 4	n = 10	n = 5	n = 6	n = 2	n = 5	n = 9	n = 41
Year of publication									
2011 – 2014	11 (30.56%)	1 (25.00%)	6 (60.00%)	1 (20.00%)	3 (50.00%)	1 (50.00%)	3 (60.00%)	3 (33.33%)	13 (31.71%)
2015 – 2018	15 (41.67%)	2 (50.00%)	3 (30.00%)	3 (60.00%)	1 (16.67%)	1 (50.00%)	2 (40.00%)	5 (55.56%)	18 (43.90%)
2019 – 2021	10 (27.78%)	1 (25.00%)	1 (10.00%)	1 (20.00%)	2 (33.33%)	-	-	1 (11.11%)	10 (24.39%)
Geographical Distribution									
Europe	12 (33.33%)	1 (25.00%)	1 (10.00%)	-	3 (50.00%)	-	3 (60.00%)	4 (44.44%)	14 (34.15%)
Asia	20 (55.56%)	2 (50.00%)	6 (60.00%)	3 (60.00%)	2 (33.33%)	-	2 (40.00%)	2 (22.22%)	20 (48.78%)
North America	1 (2.78%)	-	2 (20.00%)	1 (20.00%)	1 (16.67%)	2 (100.00)	-	1 (11.11%)	3 (7.32%)
South America	1 (2.78%)	-	1 (10.00%)	1 (20.00%)	-	-	-	1 (11.11%)	2 (4.88%)
Oceania	1 (2.78%)	-	-	-	-	-	-	1 (11.11%)	1 (2.44%)
Intercontinental	1 (2.78%)	1 (25.00%)	-	-	-	-	-	-	1 (2.44%)
Hospital type									
Secondary Care	2 (5.56%)	-	1 (10.00%)	-	-	-	-	-	2 (4.88%)
Tertiary Care	29 (80.56%)	3 (75.00%)	7 (70.00%)	2 (40.00%)	6 (100.00%)	1 (50.00%)	4 (80.00%)	5 (55.56%)	30 (73.17%)
Mixed	3 (8.33%)	1 (25.00%)	1 (10.00%)	1 (20.00%)	-	-	1 (20.00%)	-	3 (7.32%)
Unclear	2 (5.56%)	-	1 (10.00%)	2 (40.00%)	-	1 (50.00%)	-	4 (44.44%)	6 (14.63%)
Study department									
Hospital wide	30 (83.33%)	3 (75.00%)	8 (80.00%)	4 (80.00%)	5 (83.33%)	1 (50.00%)	5 (100.00%)	9 (100.00%)	35 (85.37%)
ICU	4 (11.11%)	1 (25.00%)	2 (20.00%)	1 (20.00%)	-	1 (50.00%)	-	-	4 (9.76%)
Mixed	2 (5.56%)	-	-	-	1 (16.67%)	-	-	-	2 (4.88%)

Target pathogens									
P. aeruginosa	5 (13.89%)	-	1 (10.00%)	-	-	-	5 (100.00%)	1 (11.11%)	5 (12.50%)
S. aureus	1 (2.78%)	-	-	2 (40.00%)	-	-	-	3 (33.33%)	10 (24.39%)
K. pneumoniae	10 (27.78%)	1 (25.00%)	1 (10.00%)	1 (20.00%)	1 (16.67%)	-	-	2 (22.22%)	4 (9.76%)
A. baumannii	3 (8.33%)	2 (50.00%)	3 (30.00%)	2 (40.00%)	1 (16.67%)	-	-	-	3 (7.32%)
Mixed	15 (41.67%)	1 (25.00%)	5 (50.00%)	-	4 (66.67%)	2 (100.00%)	-	1 (11.11%)	16 (39.02%)
Other	-	-	-	-	-	-	-	1 (11.11%)	1 (2.44%)
Unrestricted by pathogen	2 (5.56%)	-	-	-	-	-	-	1 (11.11%)	2 (4.88%)
Resistance mechanism of target pathogens									
Carbapenem-resistance	12 (33.33%)	1 (25.00%)	2 (20.00%)	-	5 (83.33%)	-	-	-	12 (29.27%)
ESBL-production	5 (3.89%)	1 (25.00%)	-	-	-	-	-	1 (11.11%)	5 (12.20%)
VRE	-	-	1 (10.00%)	-	1 (16.67%)	1 (50.00%)	-	-	1 (2.44%)
Unrestricted by resistance	19 (52.78%)	2 (50.00%)	7 (70.00%)	5 (100.00%)	-	1 (50.00%)	5 (100.00%)	8 (88.89%)	23 (56.10%)
Target type of infection									
Bloodstream infection	27 (75.00%)	4 (100.00%)	6 (60.00%)	5 (100.00%)	3 (50.00%)	-	4 (80.00%)	7 (77.78%)	30 (73.17%)
Respiratory infection	5 (13.89%)	-	2 (20.00%)	-	-	1 (50.00%)	1 (20.00%)	2 (22.22%)	6 (14.63%)
Urinary tract infection	-	-	1 (10.00%)	-	1 (16.67%)	1 (50.00%)	-	-	1 (2.44%)
Abdominal infection	1 (2.78%)	-	-	-	1 (16.67%)	-	-	-	1 (2.44%)
Mixed / Other	1 (2.78%)	-	-	-	-	-	-	-	1 (2.44%)
Unrestricted by type of infection	2 (5.56%)	-	1 (10.00%)	-	1 (16.67%)	-	-	-	2 (4.88%)
Target site of acquisition									
Healthcare-setting	8 (22.22%)	1 (25.00%)	4 (40.00%)	1 (20.00%)	2 (33.33%)	-	1 (20.00%)	1 (11.11%)	8 (19.51%)
Community acquired	1 (2.78%)	-	-	-	-	-	-	2 (22.22%)	2 (4.88%)
Unrestricted by acquisition	27 (75.00%)	3 (75.00%)	6 (60.00%)	4 (80.00%)	4 (66.67%)	2 (100.00%)	4 (80.00%)	6 (66.67%)	31 (75.61%)
Immunosuppressed and cancer patients	2 (5.56%)	-	-	-	-	-	-	-	2 (4.88%)
Appropriateness in study objectives									
AAT mentioned in study objectives	9 (25.00%)	2 (50.00%)	5 (50.00%)	2 (40.00%)	2 (33.33%)	1 (50.00%)	1 (20.00%)	2 (22.22%)	10 (24.39%)
	7 (19.44%)	2 (50.00%)	4 (40.00%)	1 (20.00%)	2 (33.33%)	1 (50.00%)	1 (20.00%)	1 (11.11%)	7 (17.07%)

Impact of AAT on outcomes in study objectives									
Source of definition specified in the paper	6 (16.67%)	1 (25.00%)	2 (20.00%)	2 (40.00%)	4 (66.67%)	1 (50.00%)	-	2 (22.22%)	8 (19.51%)
Reflection of quality/limitations of the definition in the paper	9 (25.00%)	2 (50.00%)	6 (60.00%)	1 (20.00%)	2 (33.33%)	2 (100.00%)	2 (40.00%)	-	10 (24.39%)

S Table 16: Overview of outcome measures and effect of empiric appropriate antibiotic therapy (AAT) on patient outcomes in univariate and multivariate analyses reported by the studies

	Total number of outcomes	Number of outcomes tested in multivariable analysis	Significant effect based on multivariable analysis		No significant effect based on multivariable analysis
			appropriate therapy favored	inappropriate therapy favored	
	n	n (%)	n (%)	n (%)	n (%)
**Outcomes**	**390**	**199 (51.03%)**	**125 (62.81%)**	**1 (0.50%)**	**73 (36.68%)**
All-Cause Mortality[a]	268	147 (54.85%)	102 (69.39%)	1 (0.68%)	44 (29.93%)
Early	35	22 (62.86%)	15 (68.18%)	1 (4.55%)	6 (27.27%)
Late	118	65 (55.08%)	40 (61.54%)	-	25 (38.46%)
Long-term	14	9 (64.29%)	9 (100.00%)	-	-
In-hospital	71	38 (53.52%)	27 (71.05%)	-	11 (28.95%)
ICU	22	11 (50.00%)	9 (81.82%)	-	2 (18.18%)
Unknown	8	2 (25.00%)	2 (100.00%)	-	-
Attributable Mortality	12	6 (50.00%)	4 (66.67%)	-	2 (33.33%)
Length of hospital stay	34	15 (44.12%)	10 (66.67%)	-	5 (33.33%)
Clinical failure	25	14 (56.00%)	7 (50.00%)	-	7 (50.00%)
ICU length of stay	16	4 (25.00%)	1 (25.00%)	-	3 (75.00%)
Hospital readmission	9	6 (66.67%)	-		6 (100.00%)
ICU admission	8	2 (25.00%)	-	-	2 (100.00%)
Adverse events	7	2 (28.57%)	-	-	2 (100.00%)
CDI rate	1	1 (100.00%)	-	-	1 (100.00%)
Microbiological failure	1	1 (100.00%)	1 (100.00%)	-	-
Time to clinical success	1	1 (100.00%)	-	-	1 (100.00%)
Duration of antibiotic therapy	1	-	-	-	-
Emergence of MDR	1	-	-	-	-

a. All-cause mortality was classified as: early mortality (2 - 15 days), late mortality (21 - 30 days), long-term mortality (over 30 days, including 60-day - 1-year mortality).

S Table 17: Overview of outcome measures and effect of definite appropriate antibiotic therapy (AAT) on patient outcomes in univariate and multivariate analyses reported by the studies

	Total number of outcomes	Number of outcomes tested in multivariable analysis	Significant effect based on multivariable analysis		No significant effect based on multivariable analysis
			appropriate therapy favored	inappropriate therapy favored	
	n	n (%)	n (%)	n (%)	n (%)
**Outcomes**	**53**	**26 (49.06%)**	**21 (80.77%)**	**-**	**5 (19.23%)**
All-Cause Mortality[a]	46	22 (47.83%)	18 (81.82%)	-	4 (18.18%)
Early	8	4 (50.00%)	4 (100.00%)	-	-
Late	29	13 (44.83%)	10 (76.92%)	-	3 (23.08%)
Long-term	2	-	-	-	-
In-hospital	7	5 (71.43%)	4 (80.00%)	-	1 (20.00%)
Attributable Mortality	2	2 (100.00%)	2 (100.00%)	-	-
Length of hospital stay	3	1 (33.33%)	1 (100.00%)	-	-
Clinical failure	2	1 (50.00%)	-	-	1 (100.00%)

a. All-cause mortality was classified as: early mortality (2 - 15 days), late mortality (21 - 30 days), long-term mortality (over 30 days, including 60-day - 1-year mortality).

S Table 18: Study characteristics and the measured effect of empiric appropriate antibiotic therapy (AAT) on all-cause mortality in the multivariable analyses reported by the studies

	**Effect of AAT on all-cause mortality**		
	**Significant effect found[a]**	**Significant effect not found**	
	n (%)	n (%)	p-value
	102 (69.86%)	44 (30.14%)	
**Study characteristic**			
Year of publication			
2011 – 2014	36 (78.26%)	10 (21.74%)	0.142
2015 – 2018	47 (70.15%)	20 (29.85%)	
2019 – 2021	19 (57.86%)	14 (42.42%)	
Geographical Distribution			
Europe	46 (70.77%)	19 (29.23%)	0.920
Asia	37 (68.52%)	17 (31.48%)	
North America	15 (75.00%)	5 (25.00%)	
South America	3 (60.00%)	2 (40.00%)	
Intercontinental	1 (50.00%)	1 (50.00%)	
Hospital type			
Secondary Care	3 (60.00%)	2 (40.00%)	0.309
Tertiary Care	68 (70.83%)	28 (29.17%)	
Mixed	11 (55.00%)	9 (45.00%)	
Unclear	20 (80.00%)	5 (20.00%)	
Study department			
Hospital wide	63 (67.74%)	30 (32.36%)	0.274
ICU	26 (78.79%)	7 (21.21%)	
Emergency department	4 (44.44%)	5 (55.56%)	
Mixed	8 (80.00%)	2 (20.00%)	
Unclear	1 (100.00%)	-	
Target pathogens			
P. aeruginosa	5 (83.33%)	1 (16.67%)	0.476
E. coli	2 (66.67%)	1 (33.33%)	
S. aureus	5 (62.50%)	3 (37.50%)	
K. pneumoniae	6 (85.71%)	1 (14.29%)	
A. baumannii	8 (88.89%)	1 (11.11%)	
S. pneumoniae	2 (100.00%)	-	
Mixed	22 (56.41%)	17 (43.59%)	
Other	2 (66.67%)	1 (33.33%)	
Unrestricted by pathogen	50 (72.46%)	19 (27.54%)	
Resistance mechanism of target pathogens			
Carbapenem-resistance	4 (100.00%)	-	0.132
ESBL-production	1 (25.00%)	3 (75.00%)	
MRSA	3 (60.00%)	2 (40.00%)	
Unspecified	3 (100.00%)	-	
Unrestricted by resistance	91 (70.00%)	39 (30.00%)	
Target type of infection			
Bloodstream infection	69 (68.32%)	32 (31.68%)	0.456
Respiratory infection	14 (77.78%)	4 (22.22%)	
Urinary tract infection	1 (25.00%)	3 (75.00%)	
Abdominal infection	3 (75.00%)	1 (25.00%)	

Central nervous system infection	1 (100.00%)	-	
Mixed / Other	5 (71.43%)	2 (28.57%)	
Unrestricted by type of infection	9 (81.82%)	2 (18.18%)	
Target site of acquisition			
Healthcare-setting	22 (73.33%)	8 (26.67%)	0.883
Community acquired	19 (70.37%)	8 (29.63%)	
Unrestricted by acquisition	61 (68.54%)	28 (31.46%)	
Immunosuppressed and cancer patients			
Yes	8 (57.14%)	6 (42.86%)	0.275
No	94 (71.21%)	38 (28.79%)	
AAT mentioned in study objectives			
Yes	38 (65.52%)	20 (34.48%)	0.353
No	64 (72.73%)	24 (27.27%)	
Impact of AAT on outcomes in study objectives			0.103
Yes	32 (61.54%)	20 (38.46%)	
No	70 (74.47%)	24 (25.53%)	
Source of definition specified in the paper			
Yes	26 (65.00%)	14 (35.00%)	0.431
No	76 (71.70%)	30 (28.30%)	
Reflection of quality/limitations of the definition in the paper			0.014
Yes	18 (52.94%)	16 (47.06%)	
No	84 (75.00%)	28 (25.00%)	
Definition of appropriateness			
Based on susceptibility only	35 (71.43%)	14 (28.57%)	
Susceptibility + 1 aspect	32 (66.67%)	16 (33.33%)	0.948
Susceptibility + ≥ 2 aspects	30 (71.43%)	12 (28.57%)	
Non-susceptibility-centered	4 (66.67%)	2 (33.33%)	
Aspects considered			
Susceptibility	97 (69.78%)	42 (30.22%)	0.871
Timely initiation	38 (67.86%)	18 (32.14%)	0.709
Dosing	27 (71.05%)	11 (28.95%)	0.827
Route of Administration	15 (60.00%)	10 (40.00%)	0.248
Duration of Treatment	8 (100.00%)	-	0.055
Guideline-based choice	6 (75.00%)	2 (25.00%)	0.735
Aminoglycoside restriction	10 (90.91%)	1 (9.09%)	0.111
Other	9 (60.00%)	6 (40.00%)	0.390

a. Excluding data from one study in which IAT was found to significantly reduce mortality.

S Table 19: Covariates considered as potential risk factors for unfavorable outcome among studies reporting multivariable analysis

Outcome	Covariate Category	Number of analyses (%) considering the covariate[a]
All-cause mortality (n = 179)	Disease severity	149/157 (94.91%)
	Scoring system	117 (78.52%)
	Critical disease	101 (67.79%)
	Specific organ failure	50 (33.56%)
	Laboratory values indicating severe disease	32 (21.48%)
	Vital parameters indicating severe disease	32 (21.48%)
	Other	16 (10.74%)
	Comorbidities	138/152 (90.79%)
	Scoring system	77 (55.80%)
	Severe underlying disease	77 (55.80%)
	Poor functional status	21 (15.22%)
	Specific comorbid condition	99 (71.74%)
	Other	19 (13.77%)
	Infection with resistant pathogen	78/135 (57.78%)
	Immunosuppression	85/146 (58.22%)
	Compound measure	25 (29.41%)
	Immunosuppressive medication	56 (65.88%)
	Neutropenia and neutropenic sepsis	44 (51.76%)
	Immunodeficiency disorder (conatal/acquired)	10 (11.76%)
	Other	1 (1.18%)
Length of stay (n = 17)	Disease severity	12/16 (75.00%)
	Scoring system	5 (41.67%)
	Critical disease	3 (25.00%)
	Vital parameters indicating severe disease	3 (25.00%)
	Other	4 (33.33%)
	Comorbidities	14/16 (87.50%)
	Scoring system	3 (21.43%)
	Severe underlying disease	4 (28.57%)
	Poor functional status	4 (28.57%)
	Specific comorbid condition	8 (57.14%)
	Other	6 (42.86%)
	Infection with resistant pathogen	4/15 (26.67%)
	Immunosuppression	5/14 (35.71%)
	Compound measure	4 (80.00%)
	Immunosuppressive medication	2 (40.00%)
	Neutropenia and neutropenic sepsis	2 (40.00%)
Clinical failure (n = 8)	Disease severity	6/8 (75.00%)
	Scoring system	3 (50.00%)
	Critical disease	2 (33.33%)
	Lab values indicating severe disease	1 (16.67%)
	Vital parameters indicating severe disease	1 (16.67%)
	Other	2 (33.33%)
	Comorbidities	6/8 (75.00%)
	Scoring system	1 (16.67%)
	Severe underlying disease	2 (33.33%)
	Poor functional status	1 (16.67%)
	Specific comorbid condition	5 (83.33%)

	Infection with resistant pathogen	6/7 (85.71%)
	Immunosuppression	4/6 (66.67%)
	Compound measure<br>Neutropenia and neutropenic sepsis	3 (75.00%)<br>1 (25.00%)

a. Total number of studies varies based on how often a category was found to be non-applicable, e.g. infection with resistant pathogen in a cohort including resistant infections only. Additionally, some studies did not detail the aspects considered for analysis and if the covariates were considered was therefore deemed unknown.

S Table 20: Relationship between the considered risk factors for mortality and the measured effect of appropriate antibiotic therapy (AAT) on all-cause mortality in multivariable analysis reported in the studies[a]

		Effect of AAT on all-cause mortality		
		Significant effect found[b]	Significant effect not found	
		n (%)	n (%)	p-value
**Overall treatment**		130 (72.63%)	49 (27.37%)	
Risk factors for mortality considered[c]				
Severity of disease	Yes<br>No	113 (75.84%)<br>3 (37.50%)	36 (24.16%)<br>5 (62.50%)	0.016
Comorbidities	Yes<br>No	100 (72.46%)<br>12 (85.71%)	38 (27.54%)<br>2 (14.29%)	0.283
Immunosuppression	Yes<br>No	67 (78.82%)<br>41 (67.21%)	18 (21.18%)<br>20 (32.79%)	0.115
Infection with resistant pathogen	Yes<br>No	57 (73.08%)<br>44 (77.19%)	21 (26.92%)<br>13 (22.81%)	0.586
Independent risk factors for mortality among all studies with multivariable analysis[c, d]				
Severity of disease	Yes<br>No	110 (78.57%)<br>20 (66.67%)	30 (21.43%)<br>10 (33.33%)	0.163
Comorbidities	Yes<br>No	56 (70.89%)<br>74 (81.32%)	23 (29.11%)<br>17 (18.68%)	0.110
Immunosuppression	Yes<br>No	17 (80.95%)<br>113 (75.84%)	4 (19.05%)<br>36 (24.16%)	0.605
Infection with resistant pathogen	Yes<br>No	17 (70.83%)<br>113 (77.40%)	7 (29.17%)<br>33 (22.60%)	0.482
Independent risk factors for mortality among studies considering the respective aspects upon univariable analysis[c, d]				
Severity of disease	Yes<br>No	103 (79.84%)<br>10 (66.67%)	26 (20.16%)<br>5 (33.33%)	0.240
Comorbidities	Yes<br>No	52 (72.22%)<br>48 (81.36%)	20 (27.78%)<br>11 (18.64%)	0.221
Immunosuppression	Yes<br>No	15 (78.95%)<br>52 (82.54%)	4 (21.05%)<br>11 (17.46%)	0.723
Infection with resistant pathogen	Yes<br>No	16 (72.73%)<br>41 (73.21%)	6 (27.27%)<br>15 (26.79%)	0.965
**Empiric treatment**		102 (69.86%)	44 (30.14%)	
Risk factors for mortality considered[c]				
Severity of disease	Yes<br>No	85 (72.65%)<br>3 (37.50%)	32 (27.35%)<br>5 (62.50%)	0.035
Comorbidities	Yes<br>No	75 (68.81%)<br>9 (81.82%)	34 (31.19%)<br>2 (18.18%)	0.369
Immunosuppression	Yes<br>No	50 (76.92%)<br>31 62.00%)	15 (23.08%)<br>19 (38.00%)	0.082
Infection with resistant pathogen	Yes<br>No	44 (69.84%)<br>38 (74.51%)	19 (30.16%)<br>13 (25.49%)	0.581
Independent risk factors for mortality among all studies with multivariable analysis[c, d]				
Severity of disease	Yes<br>No	84 (75.68%)<br>18 (66.67%)	27 (24.32%)<br>9 (33.33%)	0.339

Comorbidities	Yes<br>No	44 (67.69%)<br>58 (79.45%)	21 (32.31%)<br>15 (20.55%)	0.116
Immunosuppression	Yes<br>No	14 (77.78%)<br>88 (73.33%)	4 (22.22%)<br>32 (26.67%)	0.689
Infection with resistant pathogen	Yes<br>No	12 (66.67%)<br>90 (75.00%)	6 (33.33%)<br>30 (25.00%)	0.453
Independent risk factors for mortality among studies considering the respective aspects upon univariable analysis[c, d]				
Severity of disease	Yes<br>No	77 (77.00%)<br>8 (66.67%)	23 (23.00%)<br>4 (33.33%)	0.429
Comorbidities	Yes<br>No	40 (68.97%)<br>35 (79.55%)	18 (31.03%)<br>9 (20.45%)	0.230
Immunosuppression	Yes<br>No	12 (75.00%)<br>38 (82.61%)	4 (25.00%)<br>8 (17.39%)	0.507
Infection with resistant pathogen	Yes<br>No	11 (68.75%)<br>33 (70.21%)	5 (31.25%)<br>14 (29.79%)	0.912

a. Studies in which consideration of the specific risk factor were unknown or not applicable were excluded.

b. Excluding data from one study in which IAT was found to significantly reduce mortality.

c. 6 studies investigated both the effect of empiric and definite therapy within the same analysis, therefore contributing twice.

d. 7 studies did not provide data on factors found significant upon multivariable analysis other than AAT.

References

1. De Rosa, F.G., et al., *The effect of inappropriate therapy on bacteremia by ESBL-producing bacteria.* Infection, 2011. **39**(6): p. 555-61.
2. Fayad, G., et al., *Characteristics and prognosis of patients requiring valve surgery during active infective endocarditis.* J Heart Valve Dis, 2011. **20**(2): p. 223-8.
3. Fernández-Hidalgo, N., et al., *Prognosis of left-sided infective endocarditis in patients transferred to a tertiary-care hospital--prospective analysis of referral bias and influence of inadequate antimicrobial treatment.* Clin Microbiol Infect, 2011. **17**(5): p. 769-75.
4. Johnson, M.T., et al., *Impact of previous antibiotic therapy on outcome of Gram-negative severe sepsis.* Crit Care Med, 2011. **39**(8): p. 1859-65.
5. Joo, E.J., et al., *Impact of inappropriate empiric antimicrobial therapy on outcome in Pseudomonas aeruginosa bacteraemia: a stratified analysis according to sites of infection.* Infection, 2011. **39**(4): p. 309-18.
6. Lin, J.N., et al., *Clinical characteristics and outcomes of patients with extended-spectrum beta-lactamase-producing bacteremias in the emergency department.* Internal and Emergency Medicine, 2011. **6**(6): p. 547-555.
7. Micek, S.T., R.M. Reichley, and M.H. Kollef, *Health care-associated pneumonia (HCAP): empiric antibiotics targeting methicillin-resistant Staphylococcus aureus (MRSA) and Pseudomonas aeruginosa predict optimal outcome.* Medicine (Baltimore), 2011. **90**(6): p. 390-395.
8. Montravers, P., et al., *Strategies of initiation and streamlining of antibiotic therapy in 41 French intensive care units.* Crit Care, 2011. **15**(1): p. R17.
9. Plataki, M., et al., *Predictors of acute kidney injury in septic shock patients: an observational cohort study.* Clin J Am Soc Nephrol, 2011. **6**(7): p. 1744-51.
10. Reisfeld, S., et al., *The effect of empiric antibiotic therapy on mortality in debilitated patients with dementia.* Eur J Clin Microbiol Infect Dis, 2011. **30**(6): p. 813-8.
11. Rello, J., et al., *Determinants of prescription and choice of empirical therapy for hospital-acquired and ventilator-associated pneumonia.* Eur Respir J, 2011. **37**(6): p. 1332-9.
12. Schechner, V., et al., *Pseudomonas aeruginosa bacteremia upon hospital admission: risk factors for mortality and influence of inadequate empirical antimicrobial therapy.* Diagn Microbiol Infect Dis, 2011. **71**(1): p. 38-45.
13. Schreiber, M.P., C.M. Chan, and A.F. Shorr, *Bacteremia in Staphylococcus aureus pneumonia: outcomes and epidemiology.* J Crit Care, 2011. **26**(4): p. 395-401.
14. Seligman, R., B.G. Seligman, and P.J. Teixeira, *Comparing the accuracy of predictors of mortality in ventilator-associated pneumonia.* J Bras Pneumol, 2011. **37**(4): p. 495-503.
15. Shorr, A.F., et al., *Inappropriate antibiotic therapy in Gram-negative sepsis increases hospital length of stay.* Crit Care Med, 2011. **39**(1): p. 46-51.
16. Suppli, M., et al., *Mortality in enterococcal bloodstream infections increases with inappropriate antimicrobial therapy.* Clin Microbiol Infect, 2011. **17**(7): p. 1078-83.
17. Tumbarello, M., et al., *Multidrug-resistant Pseudomonas aeruginosa bloodstream infections: risk factors and mortality.* Epidemiol Infect, 2011. **139**(11): p. 1740-9.
18. Wang, S.S., et al., *Clinical manifestations and prognostic factors in cancer patients with bacteremia due to extended-spectrum β-lactamase-producing Escherichia coli or Klebsiella pneumoniae.* J Microbiol Immunol Infect, 2011. **44**(4): p. 282-8.
19. Zarkotou, O., et al., *Predictors of mortality in patients with bloodstream infections caused by KPC-producing Klebsiella pneumoniae and impact of appropriate antimicrobial treatment.* Clin Microbiol Infect, 2011. **17**(12): p. 1798-803.
20. Aguilar-Duran, S., et al., *Community-onset healthcare-related urinary tract infections: comparison with community and hospital-acquired urinary tract infections.* J Infect, 2012. **64**(5): p. 478-83.

21. Ariza, X., et al., *Risk factors for resistance to ceftriaxone and its impact on mortality in community, healthcare and nosocomial spontaneous bacterial peritonitis.* J Hepatol, 2012. **56**(4): p. 825-32.
22. Bouza, E., et al., *Ventilator-associated pneumonia due to meticillin-resistant Staphylococcus aureus: risk factors and outcome in a large general hospital.* J Hosp Infect, 2012. **80**(2): p. 150-5.
23. Castillo, J.S., et al., *Mortality among critically ill patients with methicillin-resistant Staphylococcus aureus bacteremia: a multicenter cohort study in Colombia.* Rev Panam Salud Publica, 2012. **32**(5): p. 343-50.
24. Chidiac, C., et al., *Factors associated with hospital mortality in community-acquired legionellosis in France.* European Respiratory Journal, 2012. **39**(4): p. 963-970.
25. Chuang, Y.C., S.C. Chang, and W.K. Wang, *Using the rate of bacterial clearance determined by real-time polymerase chain reaction as a timely surrogate marker to evaluate the appropriateness of antibiotic usage in critical patients with Acinetobacter baumannii bacteremia.* Crit Care Med, 2012. **40**(8): p. 2273-80.
26. de Gouvêa, E.F., et al., *The influence of carbapenem resistance on mortality in solid organ transplant recipients with Acinetobacter baumannii infection.* BMC Infect Dis, 2012. **12**: p. 351.
27. Gozel, M.G., et al., *Risk Factors for Mortality in Patients with Nosocomial Gram-Negative Bacteremia.* Turkiye Klinikleri Tip Bilimleri Dergisi, 2012. **32**(6): p. 1641-1647.
28. Halilovic, J., B.H. Heintz, and J. Brown, *Risk factors for clinical failure in patients hospitalized with cellulitis and cutaneous abscess.* Journal of Infection, 2012. **65**(2): p. 128-134.
29. Hernández-Torres, A., et al., *Multidrug and carbapenem-resistant Acinetobacter baumannii infections: Factors associated with mortality.* Med Clin (Barc), 2012. **138**(15): p. 650-5.
30. Horino, T., et al., *Clinical characteristics and risk factors for mortality in patients with bacteremia caused by Pseudomonas aeruginosa.* Intern Med, 2012. **51**(1): p. 59-64.
31. Huang, S.T., et al., *Risk factors and clinical outcomes of patients with carbapenem-resistant Acinetobacter baumannii bacteremia.* J Microbiol Immunol Infect, 2012. **45**(5): p. 356-62.
32. Jung, Y., et al., *Differences in characteristics between healthcare-associated and community-acquired infection in community-onset Klebsiella pneumoniae bloodstream infection in Korea.* BMC Infect Dis, 2012. **12**: p. 239.
33. Kim, Y.J., et al., *Risk factors for mortality in patients with carbapenem-resistant Acinetobacter baumannii bacteremia: impact of appropriate antimicrobial therapy.* J Korean Med Sci, 2012. **27**(5): p. 471-5.
34. Lee, N.Y., et al., *Carbapenem therapy for bacteremia due to extended-spectrum-β-lactamase-producing Escherichia coli or Klebsiella pneumoniae: implications of ertapenem susceptibility.* Antimicrob Agents Chemother, 2012. **56**(6): p. 2888-93.
35. Lye, D.C., et al., *The impact of multidrug resistance in healthcare-associated and nosocomial Gram-negative bacteraemia on mortality and length of stay: cohort study.* Clin Microbiol Infect, 2012. **18**(5): p. 502-8.
36. Micek, S., et al., *An institutional perspective on the impact of recent antibiotic exposure on length of stay and hospital costs for patients with gram-negative sepsis.* BMC Infect Dis, 2012. **12**: p. 56.
37. O'Neal, C.S., et al., *Treatment outcomes in patients with third-generation cephalosporin-resistant Enterobacter bacteremia.* Scand J Infect Dis, 2012. **44**(10): p. 726-32.
38. Park, S.Y., et al., *Impact of adequate empirical combination therapy on mortality from bacteremic Pseudomonas aeruginosa pneumonia.* BMC Infect Dis, 2012. **12**: p. 308.
39. Qureshi, Z.A., et al., *Clinical characteristics of bacteraemia caused by extended-spectrum β-lactamase-producing Enterobacteriaceae in the era of CTX-M-type and KPC-type β-lactamases.* Clin Microbiol Infect, 2012. **18**(9): p. 887-93.
40. Sancho, S., et al., *Impact of nosocomial polymicrobial bloodstream infections on the outcome in critically ill patients.* Eur J Clin Microbiol Infect Dis, 2012. **31**(8): p. 1791-6.

41. Silveira, C.D., C.S. Ferreira, and A. Corrêa Rde, *Adherence to guidelines and its impact on outcomes in patients hospitalized with community-acquired pneumonia at a university hospital.* J Bras Pneumol, 2012. **38**(2): p. 148-57.
42. Tabah, A., et al., *Characteristics and determinants of outcome of hospital-acquired bloodstream infections in intensive care units: the EUROBACT International Cohort Study.* Intensive Care Med, 2012. **38**(12): p. 1930-45.
43. Tumbarello, M., et al., *Multidrug-resistant Proteus mirabilis bloodstream infections: risk factors and outcomes.* Antimicrob Agents Chemother, 2012. **56**(6): p. 3224-31.
44. Wu, U.I., et al., *Ertapenem in the treatment of bacteremia caused by extended-spectrum beta-lactamase-producing Escherichia coli: a propensity score analysis.* Int J Infect Dis, 2012. **16**(1): p. e47-52.
45. Zervos, M.J., et al., *Epidemiology and outcomes of complicated skin and soft tissue infections in hospitalized patients.* J Clin Microbiol, 2012. **50**(2): p. 238-45.
46. Adrie, C., et al., *Initial use of one or two antibiotics for critically ill patients with community-acquired pneumonia: impact on survival and bacterial resistance.* Crit Care, 2013. **17**(6): p. R265.
47. Bowers, D.R., et al., *Outcomes of appropriate empiric combination versus monotherapy for Pseudomonas aeruginosa bacteremia.* Antimicrob Agents Chemother, 2013. **57**(3): p. 1270-4.
48. Capone, A., et al., *High rate of colistin resistance among patients with carbapenem-resistant Klebsiella pneumoniae infection accounts for an excess of mortality.* Clin Microbiol Infect, 2013. **19**(1): p. E23-e30.
49. Cardoso, T., et al., *The impact of healthcare-associated infection on mortality: failure in clinical recognition is related with inadequate antibiotic therapy.* PLoS One, 2013. **8**(3): p. e58418.
50. Ferreira, J.P., et al., *Left-sided infective endocarditis: analysis of in-hospital and medium-term outcome and predictors of mortality.* Rev Port Cardiol, 2013. **32**(10): p. 777-84.
51. Gasch, O., et al., *Predictive factors for early mortality among patients with methicillin-resistant Staphylococcus aureus bacteraemia.* J Antimicrob Chemother, 2013. **68**(6): p. 1423-30.
52. Gasch, O., et al., *Predictive factors for mortality in patients with methicillin-resistant Staphylococcus aureus bloodstream infection: impact on outcome of host, microorganism and therapy.* Clin Microbiol Infect, 2013. **19**(11): p. 1049-57.
53. Heintz, B.H., et al., *Evaluation of the treatment of vancomycin-resistant enterococcal urinary tract infections in a large academic medical center.* Ann Pharmacother, 2013. **47**(2): p. 159-69.
54. Horcajada, J.P., et al., *Healthcare-associated, community-acquired and hospital-acquired bacteraemic urinary tract infections in hospitalized patients: a prospective multicentre cohort study in the era of antimicrobial resistance.* Clin Microbiol Infect, 2013. **19**(10): p. 962-8.
55. Kang, C.I., et al., *Clinical impact of inappropriate initial antimicrobial therapy on outcome in bacteremic biliary tract infections.* Scand J Infect Dis, 2013. **45**(3): p. 227-34.
56. Kang, C.I., et al., *Outcomes and risk factors for mortality in community-onset bacteremia caused by extended-spectrum beta-lactamase-producing Escherichia coli, with a special emphasis on antimicrobial therapy.* Scand J Infect Dis, 2013. **45**(7): p. 519-25.
57. Kuo, S.C., et al., *Evaluation of the effect of appropriate antimicrobial therapy on mortality associated with Acinetobacter nosocomialis bacteraemia.* Clin Microbiol Infect, 2013. **19**(7): p. 634-9.
58. Lee, C.C., et al., *Different impact of the appropriateness of empirical antibiotics for bacteremia among younger adults and the elderly in the ED.* Am J Emerg Med, 2013. **31**(2): p. 282-90.
59. Lee, Y.T., et al., *Bacteremic nosocomial pneumonia caused by Acinetobacter baumannii and Acinetobacter nosocomialis: a single or two distinct clinical entities?* Clin Microbiol Infect, 2013. **19**(7): p. 640-5.

60. Metan, G., et al., *Factors influencing the early mortality in haematological malignancy patients with nosocomial Gram negative bacilli bacteraemia: a retrospective analysis of 154 cases.* Braz J Infect Dis, 2013. **17**(2): p. 143-9.
61. Navarro-San Francisco, C., et al., *Bacteraemia due to OXA-48-carbapenemase-producing Enterobacteriaceae: a major clinical challenge.* Clin Microbiol Infect, 2013. **19**(2): p. E72-9.
62. Palmer, H.R., et al., *Clinical and microbiological implications of time-to-positivity of blood cultures in patients with Gram-negative bacilli bacteremia.* Eur J Clin Microbiol Infect Dis, 2013. **32**(7): p. 955-9.
63. Park, J.H., S.H. Choi, and J.W. Chung, *The impact of early adequate antimicrobial therapy on 14-day mortality in patients with monomicrobial Pseudomonas aeruginosa and Acinetobacter baumannii bacteremia.* J Infect Chemother, 2013. **19**(5): p. 843-9.
64. Peña, C., et al., *Impact of multidrug resistance on Pseudomonas aeruginosa ventilator-associated pneumonia outcome: predictors of early and crude mortality.* Eur J Clin Microbiol Infect Dis, 2013. **32**(3): p. 413-20.
65. Phua, J., et al., *Characteristics and outcomes of culture-negative versus culture-positive severe sepsis.* Crit Care, 2013. **17**(5): p. R202.
66. Retamar, P., et al., *Reappraisal of the outcome of healthcare-associated and community-acquired bacteramia: a prospective cohort study.* BMC Infect Dis, 2013. **13**: p. 344.
67. Ruiz-Giardin, J.M., et al., *Clinical diagnostic accuracy of suspected sources of bacteremia and its effect on mortality.* Eur J Intern Med, 2013. **24**(6): p. 541-5.
68. Shorr, A.F., et al., *Readmission following hospitalization for pneumonia: the impact of pneumonia type and its implication for hospitals.* Clin Infect Dis, 2013. **57**(3): p. 362-7.
69. Tumbarello, M., et al., *Clinical outcomes of Pseudomonas aeruginosa pneumonia in intensive care unit patients.* Intensive Care Med, 2013. **39**(4): p. 682-92.
70. Vallés, J., et al., *Evolution over a 15-year period of clinical characteristics and outcomes of critically ill patients with community-acquired bacteremia.* Crit Care Med, 2013. **41**(1): p. 76-83.
71. Willmann, M., et al., *Effect of metallo-beta-lactamase production and multidrug resistance on clinical outcomes in patients with Pseudomonas aeruginosa bloodstream infection: a retrospective cohort study.* Bmc Infectious Diseases, 2013. **13**: p. 9.
72. Yang, C.J., et al., *The impact of inappropriate antibiotics on bacteremia patients in a community hospital in Taiwan: an emphasis on the impact of referral information for cases from a hospital affiliated nursing home.* BMC Infect Dis, 2013. **13**: p. 500.
73. Yang, Y.S., et al., *Comparison between bacteremia caused by carbapenem resistant Acinetobacter baumannii and Acinetobacter nosocomialis.* BMC Infect Dis, 2013. **13**: p. 311.
74. Zheng, Y.L., et al., *Risk factors and mortality of patients with nosocomial carbapenem-resistant Acinetobacter baumannii pneumonia.* Am J Infect Control, 2013. **41**(7): p. e59-63.
75. Anderson, D.J., et al., *Bloodstream infections in community hospitals in the 21st century: a multicenter cohort study.* PLoS One, 2014. **9**(3): p. e91713.
76. Bloos, F., et al., *Impact of compliance with infection management guidelines on outcome in patients with severe sepsis: a prospective observational multi-center study.* Crit Care, 2014. **18**(2): p. R42.
77. Chusri, S., et al., *Clinical outcomes of hospital-acquired infection with Acinetobacter nosocomialis and Acinetobacter pittii.* Antimicrob Agents Chemother, 2014. **58**(7): p. 4172-9.
78. Corrêa Rde, A., et al., *Quantitative culture of endotracheal aspirate and BAL fluid samples in the management of patients with ventilator-associated pneumonia: a randomized clinical trial.* J Bras Pneumol, 2014. **40**(6): p. 643-51.
79. De Bus, L., et al., *Development of antibiotic treatment algorithms based on local ecology and respiratory surveillance cultures to restrict the use of broad-spectrum antimicrobial drugs in the treatment of hospital-acquired pneumonia in the intensive care unit: a retrospective analysis.* Crit Care, 2014. **18**(4): p. R152.

80. Esparcia, A., et al., *Influence of inadequate antimicrobial therapy on prognosis in elderly patients with severe urinary tract infections.* Eur J Intern Med, 2014. **25**(6): p. 523-7.
81. Falcone, M., et al., *Role of empirical and targeted therapy in hospitalized patients with bloodstream infections caused by ESBL-producing Enterobacteriaceae.* Ann Ig, 2014. **26**(4): p. 293-304.
82. Fayad, G., et al., *Impact of antimicrobial therapy on prognosis of patients requiring valve surgery during active infective endocarditis.* J Thorac Cardiovasc Surg, 2014. **147**(1): p. 254-8.
83. Garnacho-Montero, J., et al., *De-escalation of empirical therapy is associated with lower mortality in patients with severe sepsis and septic shock.* Intensive Care Med, 2014. **40**(1): p. 32-40.
84. Girometti, N., et al., *Klebsiella pneumoniae Bloodstream Infection Epidemiology and Impact of Inappropriate Empirical Therapy.* Medicine, 2014. **93**(17): p. 298-308.
85. Gonçalves-Pereira, J., et al., *Impact of infection on admission and of the process of care on mortality of patients admitted to the Intensive Care Unit: the INFAUCI study.* Clin Microbiol Infect, 2014. **20**(12): p. 1308-15.
86. Hsu, M.S., et al., *Sequential time to positivity of blood cultures can be a predictor of prognosis of patients with persistent Staphylococcus aureus bacteraemia.* Clin Microbiol Infect, 2014. **20**(9): p. 892-8.
87. Jeong, B.H., et al., *Comparison of severe healthcare-associated pneumonia with severe community-acquired pneumonia.* Lung, 2014. **192**(2): p. 313-20.
88. Kim, Y.J., et al., *Risk factors for mortality in patients with Pseudomonas aeruginosa bacteremia; retrospective study of impact of combination antimicrobial therapy.* BMC Infect Dis, 2014. **14**: p. 161.
89. Lee, H.Y., et al., *Risk factors and outcome analysis of acinetobacter baumannii complex bacteremia in critical patients.* Crit Care Med, 2014. **42**(5): p. 1081-8.
90. Lipsky, B.A., et al., *Economic outcomes of inappropriate initial antibiotic treatment for complicated skin and soft tissue infections: a multicenter prospective observational study.* Diagn Microbiol Infect Dis, 2014. **79**(2): p. 266-72.
91. Membrilla-Fernández, E., et al., *Effect of initial empiric antibiotic therapy combined with control of the infection focus on the prognosis of patients with secondary peritonitis.* Surg Infect (Larchmt), 2014. **15**(6): p. 806-14.
92. Nygård, S.T., et al., *Aetiology, antimicrobial therapy and outcome of patients with community acquired severe sepsis: a prospective study in a Norwegian university hospital.* BMC Infect Dis, 2014. **14**: p. 121.
93. Park, D.W., et al., *Impact of serial measurements of lysophosphatidylcholine on 28-day mortality prediction in patients admitted to the intensive care unit with severe sepsis or septic shock.* J Crit Care, 2014. **29**(5): p. 882.e5-11.
94. Pelegrín, I., et al., *Listeria monocytogenes meningoencephalitis in adults: analysis of factors related to unfavourable outcome.* Infection, 2014. **42**(5): p. 817-27.
95. Shorr, A.F., et al., *Predictors of hospital mortality among septic ICU patients with Acinetobacter spp. bacteremia: a cohort study.* BMC Infect Dis, 2014. **14**: p. 572.
96. Spoorenberg, V., et al., *Appropriate antibiotic use for patients with urinary tract infections reduces length of hospital stay.* Clin Infect Dis, 2014. **58**(2): p. 164-9.
97. Vallés, J., et al., *Epidemiology, antibiotic therapy and clinical outcomes of healthcare-associated pneumonia in critically ill patients: a Spanish cohort study.* Intensive Care Med, 2014. **40**(4): p. 572-81.
98. Van Aken, S., et al., *Risk factors, outcome and impact of empirical antimicrobial treatment in extended-spectrum beta-lactamase-producing Escherichia coli bacteraemia.* Scandinavian Journal of Infectious Diseases, 2014. **46**(11): p. 753-762.
99. Vilella, A.L. and C.F. Seifert, *Timing and appropriateness of initial antibiotic therapy in newly presenting septic patients.* Am J Emerg Med, 2014. **32**(1): p. 7-13.

100. Yokota, P.K., et al., *Impact of appropriate antimicrobial therapy for patients with severe sepsis and septic shock--a quality improvement study.* PLoS One, 2014. **9**(11): p. e104475.
101. Zeng, Q., F. Xu, and S. Jiang, *The impact of previous hospitalization in the preceding 90 days on the outcome in critically ill patients with gram-negative bloodstream infection.* Diagn Microbiol Infect Dis, 2014. **80**(2): p. 136-40.
102. Zilberberg, M.D., et al., *Multi-drug resistance, inappropriate initial antibiotic therapy and mortality in Gram-negative severe sepsis and septic shock: a retrospective cohort study.* Crit Care, 2014. **18**(6): p. 596.
103. Al-Dorzi, H.M., et al., *Impact of empirical antimicrobial therapy on the outcome of critically ill patients with Acinetobacter bacteremia.* Annals of Thoracic Medicine, 2015. **10**(4): p. 256-262.
104. Allou, N., et al., *Postoperative pneumonia following cardiac surgery in non-ventilated patients versus mechanically ventilated patients: is there any difference?* Crit Care, 2015. **19**(1): p. 116.
105. Andria, N., et al., *Mortality burden related to infection with carbapenem-resistant Gram-negative bacteria among haematological cancer patients: a retrospective cohort study.* J Antimicrob Chemother, 2015. **70**(11): p. 3146-53.
106. Bass, S.N., et al., *Impact of combination antimicrobial therapy on mortality risk for critically ill patients with carbapenem-resistant bacteremia.* Antimicrob Agents Chemother, 2015. **59**(7): p. 3748-53.
107. Bastug, A., et al., *Emergence of multidrug resistant isolates and mortality predictors in patients with solid tumors or hematological malignancies.* J Infect Dev Ctries, 2015. **9**(10): p. 1100-7.
108. Beuving, J., et al., *Impact of same-day antibiotic susceptibility testing on time to appropriate antibiotic treatment of patients with bacteraemia: a randomised controlled trial.* Eur J Clin Microbiol Infect Dis, 2015. **34**(4): p. 831-8.
109. Boel, J., et al., *Evaluating antibiotic stewardship programs in patients with bacteremia using administrative data: a cohort study.* Eur J Clin Microbiol Infect Dis, 2015. **34**(7): p. 1475-84.
110. Brigmon, M.M., et al., *Impact of fluoroquinolone resistance in Gram-negative bloodstream infections on healthcare utilization.* Clin Microbiol Infect, 2015. **21**(9): p. 843-9.
111. Coccolini, F., et al., *Antibiotic resistance pattern and clinical outcomes in acute cholecystitis: 567 consecutive worldwide patients in a prospective cohort study.* Int J Surg, 2015. **21**: p. 32-7.
112. Denis, B., et al., *Prevalence, risk factors, and impact on clinical outcome of extended-spectrum beta-lactamase-producing Escherichia coli bacteraemia: a five-year study.* Int J Infect Dis, 2015. **39**: p. 1-6.
113. Dimopoulos, G., et al., *Bloodstream infections in ICU with increased resistance: epidemiology and outcomes.* Minerva Anestesiologica, 2015. **81**(4): p. 405-418.
114. Inchai, J., et al., *Ventilator-associated pneumonia: epidemiology and prognostic indicators of 30-day mortality.* Jpn J Infect Dis, 2015. **68**(3): p. 181-6.
115. Katsiari, M., et al., *Carbapenem-resistant Klebsiella pneumoniae infections in a Greek intensive care unit: Molecular characterisation and treatment challenges.* Journal of Global Antimicrobial Resistance, 2015. **3**(2): p. 123-127.
116. Lee, H.Y., et al., *Impact of Molecular Epidemiology and Reduced Susceptibility to Glycopeptides and Daptomycin on Outcomes of Patients with Methicillin-Resistant Staphylococcus aureus Bacteremia.* PLoS One, 2015. **10**(8): p. e0136171.
117. Martin-Loeches, I., et al., *Resistance patterns and outcomes in intensive care unit (ICU)-acquired pneumonia. Validation of European Centre for Disease Prevention and Control (ECDC) and the Centers for Disease Control and Prevention (CDC) classification of multidrug resistant organisms.* J Infect, 2015. **70**(3): p. 213-22.

118. Martin-Loeches, I., et al., *Incidence and prognosis of ventilator-associated tracheobronchitis (TAVeM): a multicentre, prospective, observational study.* Lancet Respir Med, 2015. **3**(11): p. 859-68.
119. Oliveira, M.C., et al., *Enterobacteriaceae resistant to third generation cephalosporins upon hospital admission: risk factors and clinical outcomes.* Braz J Infect Dis, 2015. **19**(3): p. 239-45.
120. Park, S.Y., et al., *Coagulase-negative staphylococcal bacteremia: risk factors for mortality and impact of initial appropriate antimicrobial therapy on outcome.* Eur J Clin Microbiol Infect Dis, 2015. **34**(7): p. 1395-401.
121. Rabello, L.S., et al., *Clinical outcomes and microbiological characteristics of severe pneumonia in cancer patients: a prospective cohort study.* PLoS One, 2015. **10**(3): p. e0120544.
122. Ratzinger, F., et al., *Sepsis in standard care: patients' characteristics, effectiveness of antimicrobial therapy and patient outcome-a cohort study.* Infection, 2015. **43**(3): p. 345-352.
123. Shindo, Y., et al., *Risk factors for 30-day mortality in patients with pneumonia who receive appropriate initial antibiotics: an observational cohort study.* Lancet Infect Dis, 2015. **15**(9): p. 1055-1065.
124. Su, T.Y., et al., *Clinical characteristics and risk factors for mortality in cefepime-resistant Pseudomonas aeruginosa bacteremia.* J Microbiol Immunol Infect, 2015. **48**(2): p. 175-82.
125. Suberviola Cañas, B., et al., *Effects of antibiotic administration delay and inadequacy upon the survival of septic shock patients.* Med Intensiva, 2015. **39**(8): p. 459-66.
126. Sumida, K., et al., *Risk Factors Associated with Stenotrophomonas maltophilia Bacteremia: A Matched Case-Control Study.* PLoS One, 2015. **10**(7): p. e0133731.
127. Torres, A., et al., *Bacteraemia and antibiotic-resistant pathogens in community acquired pneumonia: risk and prognosis.* Eur Respir J, 2015. **45**(5): p. 1353-63.
128. Tumbarello, M., et al., *Infections caused by KPC-producing Klebsiella pneumoniae: differences in therapy and mortality in a multicentre study.* J Antimicrob Chemother, 2015. **70**(7): p. 2133-43.
129. Wu, J.N., et al., *Epidemiology and microbiology of nosocomial bloodstream infections: analysis of 482 cases from a retrospective surveillance study.* Journal of Zhejiang University-Science B, 2015. **16**(1): p. 70-77.
130. Abraham, K., et al., *Impact of inappropriate initial antibiotics in critically ill surgical patients with bacteremia.* Am J Surg, 2016. **211**(3): p. 593-8.
131. Ali, H.S., et al., *Epidemiology and Outcome of Ventilator-Associated Pneumonia in a Heterogeneous ICU Population in Qatar.* Biomed Res Int, 2016. **2016**: p. 8231787.
132. Cheng, W.L., et al., *Bacteremic pneumonia caused by extended-spectrum beta-lactamase-producing Escherichia coli and Klebsiella pneumoniae: Appropriateness of empirical treatment matters.* J Microbiol Immunol Infect, 2016. **49**(2): p. 208-15.
133. Chin, T., et al., *Antibiotic Utilization Patterns in Patients with Ventilator-Associated Pneumonia: A Canadian Context.* Canadian Journal of Infectious Diseases & Medical Microbiology, 2016. **2016**: p. 10.
134. Coccolini, F., et al., *Antibiotic resistance evaluation and clinical analysis of acute appendicitis; report of 1431 consecutive worldwide patients: A cohort study.* Int J Surg, 2016. **26**: p. 6-11.
135. Cuervo, G., et al., *Clinical characteristics, treatment and outcomes of MRSA bacteraemia in the elderly.* J Infect, 2016. **72**(3): p. 309-16.
136. De Rosa, F.G., et al., *Risk factors for mortality in patients with Staphylococcus aureus bloodstream infection.* J Chemother, 2016. **28**(3): p. 187-90.
137. Fitzpatrick, J.M., et al., *Gram-negative bacteraemia; a multi-centre prospective evaluation of empiric antibiotic therapy and outcome in English acute hospitals.* Clin Microbiol Infect, 2016. **22**(3): p. 244-51.
138. Freire, M.P., et al., *Bloodstream infection caused by extensively drug-resistant Acinetobacter baumannii in cancer patients: high mortality associated with delayed treatment rather than with the degree of neutropenia.* Clin Microbiol Infect, 2016. **22**(4): p. 352-358.

139. Garnacho-Montero, J., et al., *Acinetobacter baumannii in critically ill patients: Molecular epidemiology, clinical features and predictors of mortality.* Enferm Infecc Microbiol Clin, 2016. **34**(9): p. 551-558.
140. Gonzalez, C., et al., *Prognostic impact of left ventricular diastolic function in patients with septic shock.* Annals of Intensive Care, 2016. **6**: p. 8.
141. Guilbart, M., et al., *Compliance with an empirical antimicrobial protocol improves the outcome of complicated intra-abdominal infections: a prospective observational study.* Br J Anaesth, 2016. **117**(1): p. 66-72.
142. Guillamet, C.V., et al., *A cohort study of bacteremic pneumonia: The importance of antibiotic resistance and appropriate initial therapy?* Medicine (Baltimore), 2016. **95**(35): p. e4708.
143. Herkel, T., et al., *Epidemiology of hospital-acquired pneumonia: Results of a Central European multicenter, prospective, observational study compared with data from the European region.* Biomed Pap Med Fac Univ Palacky Olomouc Czech Repub, 2016. **160**(3): p. 448-55.
144. Li, M., et al., *Risk factors for slowly resolving pneumonia in the intensive care unit.* J Microbiol Immunol Infect, 2016. **49**(5): p. 654-662.
145. Li, Z., et al., *Empirical Combination Antibiotic Therapy Improves the Outcome of Nosocomial Meningitis or Ventriculitis in Neuro-Critical Care Unit Patients.* Surg Infect (Larchmt), 2016. **17**(4): p. 465-72.
146. Maeda, M., et al., *Effect of interventions by an antimicrobial stewardship team on clinical course and economic outcome in patients with bloodstream infection.* J Infect Chemother, 2016. **22**(2): p. 90-5.
147. Oshima, T., et al., *Empiric Antibiotic Therapy for Severe Sepsis and Septic Shock.* Surg Infect (Larchmt), 2016. **17**(2): p. 210-6.
148. Palacios-Baena, Z.R., et al., *Comprehensive clinical and epidemiological assessment of colonisation and infection due to carbapenemase-producing Enterobacteriaceae in Spain.* J Infect, 2016. **72**(2): p. 152-60.
149. Ruangchan, S., et al., *Clinical Outcomes of Community-Acquired Severe Sepsis after Implementation of a Simple Severe Sepsis Fast Track.* J Med Assoc Thai, 2016. **99**(8): p. 877-85.
150. Savage, R.D., et al., *The Effect of Inadequate Initial Empiric Antimicrobial Treatment on Mortality in Critically Ill Patients with Bloodstream Infections: A Multi-Centre Retrospective Cohort Study.* PLoS One, 2016. **11**(5): p. e0154944.
151. Stoma, I., et al., *Risk factors for mortality in patients with bloodstream infections during the pre-engraftment period after hematopoietic stem cell transplantation.* Blood Research, 2016. **51**(2): p. 102-106.
152. Trecarichi, E.M., et al., *Bloodstream infections caused by Klebsiella pneumoniae in onco-hematological patients: clinical impact of carbapenem resistance in a multicentre prospective survey.* Am J Hematol, 2016. **91**(11): p. 1076-1081.
153. Vallés, J., et al., *Evolution over a 15-year period of the clinical characteristics and outcomes of critically ill patients with severe community-acquired pneumonia.* Med Intensiva, 2016. **40**(4): p. 238-45.
154. Worapratya, P., et al., *Appropriateness of Broad Spectrum Antibiotics for Severe Sepsis and Septic Shock in the Emergency Department.* J Med Assoc Thai, 2016. **99**(5): p. 477-83.
155. Yilmaz, M., et al., *Mortality predictors of Staphylococcus aureus bacteremia: a prospective multicenter study.* Ann Clin Microbiol Antimicrob, 2016. **15**: p. 7.
156. Yoon, Y.K., et al., *Effects of inappropriate empirical antibiotic therapy on mortality in patients with healthcare-associated methicillin-resistant Staphylococcus aureus bacteremia: a propensity-matched analysis.* BMC Infect Dis, 2016. **16**: p. 331.
157. Zarco-Márquez, S., et al., *Invasive and Complicated Pneumococcal Infection in Patients with Cancer.* Rev Invest Clin, 2016. **68**(5): p. 221-228.
158. Zilberberg, M.D., et al., *Multidrug resistance, inappropriate empiric therapy, and hospital mortality in Acinetobacter baumannii pneumonia and sepsis.* Crit Care, 2016. **20**(1): p. 221.

159. Ahn, J.H., et al., *Clinical characteristics and prognostic risk factors of healthcare-associated pneumonia in a Korean tertiary teaching hospital.* Medicine (Baltimore), 2017. **96**(42): p. e8243.
160. Babich, T., et al., *Empirical Antibiotic Treatment Does Not Improve Outcomes in Catheter-Associated Urinary Tract Infection: Prospective Cohort Study.* Clin Infect Dis, 2017. **65**(11): p. 1799-1805.
161. Battle, S.E., et al., *Association between inappropriate empirical antimicrobial therapy and hospital length of stay in Gram-negative bloodstream infections: stratification by prognosis.* J Antimicrob Chemother, 2017. **72**(1): p. 299-304.
162. Bosch-Nicolau, P., et al., *A Cohort Study of Risk Factors That Influence Empirical Treatment of Patients with Acute Pyelonephritis.* Antimicrob Agents Chemother, 2017. **61**(12).
163. Costa-de-Oliveira, S., et al., *Potential Impact of Flow Cytometry Antimicrobial Susceptibility Testing on the Clinical Management of Gram-Negative Bacteremia Using the FASTinov (R) Kit.* Frontiers in Microbiology, 2017. **8**: p. 7.
164. Deconinck, L., et al., *Impact of combination therapy and early de-escalation on outcome of ventilator-associated pneumonia caused by Pseudomonas aeruginosa.* Infect Dis (Lond), 2017. **49**(5): p. 396-404.
165. González-Del Castillo, J., et al., *Effect of the inadequacy of antibiotic therapy in the Emergency Department on hospital stays.* Enferm Infecc Microbiol Clin, 2017. **35**(4): p. 208-213.
166. Goto, M., et al., *Association of Evidence-Based Care Processes With Mortality in Staphylococcus aureus Bacteremia at Veterans Health Administration Hospitals, 2003-2014.* JAMA Intern Med, 2017. **177**(10): p. 1489-1497.
167. Gutiérrez-Gutiérrez, B., et al., *Effect of appropriate combination therapy on mortality of patients with bloodstream infections due to carbapenemase-producing Enterobacteriaceae (INCREMENT): a retrospective cohort study.* Lancet Infect Dis, 2017. **17**(7): p. 726-734.
168. Jokinen, E., et al., *Comparison of outcome and clinical characteristics of bacteremia caused by methicillin-resistant, penicillin-resistant and penicillin-susceptible Staphylococcus aureus strains.* Infectious Diseases, 2017. **49**(7): p. 493-500.
169. Joo, E.J., et al., *Impact of appropriateness of empiric therapy on outcomes in community-onset bacteremia by extended-spectrum-beta-lactamase producing Escherichia coli and Klebisella pneumoniae definitively treated with carbapenems.* European Journal of Clinical Microbiology & Infectious Diseases, 2017. **36**(11): p. 2093-2100.
170. Lachhab, Z., et al., *Bacteraemia in Intensive Care Unit: Clinical, Bacteriological, and Prognostic Prospective Study.* Canadian Journal of Infectious Diseases & Medical Microbiology, 2017. **2017**: p. 9.
171. Li, L. and H. Huang, *Risk factors of mortality in bloodstream infections caused by Klebsiella pneumonia: A single-center retrospective study in China.* Medicine (Baltimore), 2017. **96**(35): p. e7924.
172. Micozzi, A., et al., *Carbapenem-resistant Klebsiella pneumoniae in high-risk haematological patients: factors favouring spread, risk factors and outcome of carbapenem-resistant Klebsiella pneumoniae bacteremias.* BMC Infect Dis, 2017. **17**(1): p. 203.
173. Póvoa, P., et al., *Biomarkers kinetics in the assessment of ventilator-associated pneumonia response to antibiotics - results from the BioVAP study.* J Crit Care, 2017. **41**: p. 91-97.
174. Palacios-Baena, Z.R., et al., *Development and validation of the INCREMENT-ESBL predictive score for mortality in patients with bloodstream infections due to extended-spectrum-β-lactamase-producing Enterobacteriaceae.* J Antimicrob Chemother, 2017. **72**(3): p. 906-913.
175. Papadimitriou-Olivgeris, M., et al., *Carbapenemase-producing Klebsiella pneumoniae bloodstream infection in critically ill patients: risk factors and predictors of mortality.* Eur J Clin Microbiol Infect Dis, 2017. **36**(7): p. 1125-1131.

176. Pouwels, K.B., et al., *Does appropriate empiric antibiotic therapy modify intensive care unit-acquired Enterobacteriaceae bacteraemia mortality and discharge?* J Hosp Infect, 2017. **96**(1): p. 23-28.
177. Rello, J., et al., *Improved survival among ICU-hospitalized patients with community-acquired pneumonia by unidentified organisms: a multicenter case-control study.* Eur J Clin Microbiol Infect Dis, 2017. **36**(1): p. 123-130.
178. Royo-Cebrecos, C., et al., *Characteristics, aetiology, antimicrobial resistance and outcomes of bacteraemic cholangitis in patients with solid tumours: A prospective cohort study.* J Infect, 2017. **74**(2): p. 172-178.
179. Tagashira, Y., et al., *Impact of inadequate initial antimicrobial therapy on mortality in patients with bacteraemic cholangitis: a retrospective cohort study.* Clin Microbiol Infect, 2017. **23**(10): p. 740-747.
180. Thaden, J.T., et al., *Results from a 13-Year Prospective Cohort Study Show Increased Mortality Associated with Bloodstream Infections Caused by Pseudomonas aeruginosa Compared to Other Bacteria.* Antimicrob Agents Chemother, 2017. **61**(6).
181. Tuon, F.F., et al., *Risk factors for mortality in patients with ventilator-associated pneumonia caused by carbapenem-resistant Enterobacteriaceae.* Braz J Infect Dis, 2017. **21**(1): p. 1-6.
182. Wang, X., et al., *Acinetobacter baumannii bacteraemia in patients with haematological malignancy: a multicentre retrospective study from the Infection Working Party of Jiangsu Society of Hematology.* Eur J Clin Microbiol Infect Dis, 2017. **36**(7): p. 1073-1081.
183. Zhang, Y.Y., et al., *Incidence, clinical characteristics, and outcomes of nosocomial Enterococcus spp. bloodstream infections in a tertiary-care hospital in Beijing, China: a four-year retrospective study.* Antimicrobial Resistance and Infection Control, 2017. **6**: p. 11.
184. Zilberberg, M.D., et al., *Carbapenem resistance, inappropriate empiric treatment and outcomes among patients hospitalized with Enterobacteriaceae urinary tract infection, pneumonia and sepsis.* BMC Infect Dis, 2017. **17**(1): p. 279.
185. Abdulsalam, M.S., et al., *Staphylococcus aureus bacteremia in a tertiary care hospital in India.* Indian Journal of Medical Specialities, 2018. **9**(2): p. 60-64.
186. Bassetti, M., et al., *Predictors of Mortality with Staphylococcus aureus Bacteremia in Elderly Adults.* J Am Geriatr Soc, 2018. **66**(7): p. 1284-1289.
187. Bouiller, K., et al., *No effect of vancomycin MIC >= 1.5 mg/L on treatment outcome in methicillin-susceptible Staphylococcus aureus bacteraemia.* International Journal of Antimicrobial Agents, 2018. **51**(5): p. 721-726.
188. Chen, C.T., et al., *Community-acquired bloodstream infections caused by Acinetobacter baumannii: A matched case-econtrol study.* Journal of Microbiology Immunology and Infection, 2018. **51**(5): p. 629-635.
189. Claeys, K.C., et al., *Antimicrobial Stewardship Opportunities in Critically Ill Patients with Gram-Negative Lower Respiratory Tract Infections: A Multicenter Cross-Sectional Analysis.* Infectious Diseases and Therapy, 2018. **7**(1): p. 135-146.
190. Dewi, R.S., M. Radji, and R. Andalusia, *Evaluation of Antibiotic Use Among Sepsis Patients in an Intensive Care Unit: A cross-sectional study at a referral hospital in Indonesia.* Sultan Qaboos Univ Med J, 2018. **18**(3): p. e367-e373.
191. Fouks, Y., et al., *Empirical Antibiotic Treatment of Obstetric and Gynecologic Surgical Site Infections: Are the Right Pathogens Being Targeted?* Journal of Gynecologic Surgery, 2018. **34**(5): p. 229-233.
192. Garcia-Vidal, C., et al., *Risk factors for mortality in patients with acute leukemia and bloodstream infections in the era of multiresistance.* PLoS One, 2018. **13**(6): p. e0199531.
193. Garrouste-Orgeas, M., et al., *Diabetes was the only comorbid condition associated with mortality of invasive pneumococcal infection in ICU patients: a multicenter observational study from the Outcomerea research group.* Infection, 2018. **46**(5): p. 669-677.

194. Holmes, N.E., et al., *Morbidity from in-hospital complications is greater than treatment failure in patients with Staphylococcus aureus bacteraemia.* BMC Infect Dis, 2018. **18**(1): p. 107.
195. Islas-Muñoz, B., et al., *Bloodstream infections in cancer patients. Risk factors associated with mortality.* Int J Infect Dis, 2018. **71**: p. 59-64.
196. Kethireddy, S., et al., *Culture-Negative Septic Shock Compared With Culture-Positive Septic Shock: A Retrospective Cohort Study.* Crit Care Med, 2018. **46**(4): p. 506-512.
197. Lee, Y., et al., *Risk of Mortality of Catheter-Related Bloodstream Infections Caused by Acinetobacter Species: Is Early Removal of the Catheters Associated With a Better Survival Outcome?* J Intensive Care Med, 2018. **33**(6): p. 361-369.
198. Li, H., et al., *Antibiotics De-Escalation in the Treatment of Ventilator-Associated Pneumonia in Trauma Patients: A Retrospective Study on Propensity Score Matching Method.* Chin Med J (Engl), 2018. **131**(10): p. 1151-1157.
199. Papadopoulos, D., et al., *Microbiology and prognosis assessment of hospitalized patients with aspiration pneumonia: a single-center prospective cohort study.* Infez Med, 2018. **26**(2): p. 103-114.
200. Saliba, P., et al., *Mortality risk factors among non-ICU patients with nosocomial vascular catheter-related bloodstream infections: a prospective cohort study.* J Hosp Infect, 2018. **99**(1): p. 48-54.
201. Seas, C., et al., *Staphylococcus aureus bloodstream infections in Latin America: results of a multinational prospective cohort study.* J Antimicrob Chemother, 2018. **73**(1): p. 212-222.
202. Sommer, H., et al., *The Impact of Early Adequate Treatment on Extubation and Discharge Alive of Patients With Pseudomonas aeruginosa-Related Ventilator-Associated Pneumonia.* Crit Care Med, 2018. **46**(10): p. 1643-1648.
203. Tang, Y., et al., *Prognostic factors and scoring model of hematological malignancies patients with bloodstream infections.* Infection, 2018. **46**(4): p. 513-521.
204. Tschudin-Sutter, S., et al., *Combination therapy for treatment of Pseudomonas aeruginosa bloodstream infections.* PLoS One, 2018. **13**(9): p. e0203295.
205. Xu, L., et al., *Pneumonia in patients with cirrhosis: risk factors associated with mortality and predictive value of prognostic models.* Respir Res, 2018. **19**(1): p. 242.
206. Yamaga, S. and N. Shime, *Association between appropriate empiric antimicrobial therapy and mortality from bloodstream infections in the intensive care unit.* J Infect Chemother, 2018. **24**(4): p. 267-271.
207. Battle, S.E., et al., *Derivation of a quick Pitt bacteremia score to predict mortality in patients with Gram-negative bloodstream infection.* Infection, 2019. **47**(4): p. 571-578.
208. Benítez-Sala, R., et al., *Infections related to healthcare in patients hospitalized in a Urology service: resistance patterns and adequacy of empirical antibiotic treatment as a prognostic factor.* Actas Urol Esp (Engl Ed), 2019. **43**(3): p. 151-157.
209. Ben-Chetrit, E., et al., *Associated factors and clinical outcomes of bloodstream infection due to extended-spectrum β-lactamase-producing Escherichia coli and Klebsiella pneumoniae during febrile neutropenia.* Int J Antimicrob Agents, 2019. **53**(4): p. 423-428.
210. Ben-Zvi, H., et al., *Influence of GeneXpert MRSA/SA test implementation on clinical outcomes of Staphylococcus aureus bacteremia - a before-after retrospective study.* Diagn Microbiol Infect Dis, 2019. **93**(2): p. 120-124.
211. Brescini, L., et al., *Clinical and epidemiological characteristics of KPC-producing Klebsiella pneumoniae from bloodstream infections in a tertiary referral center in Italy.* BMC Infect Dis, 2019. **19**(1): p. 611.
212. Callejas-Díaz, A., et al., *Impact of Pseudomonas aeruginosa bacteraemia in a tertiary hospital: Mortality and prognostic factors.* Med Clin (Barc), 2019. **152**(3): p. 83-89.
213. Castaño, P., et al., *Antimicrobial agent prescription: a prospective cohort study in patients with sepsis and septic shock.* Trop Med Int Health, 2019. **24**(2): p. 175-184.

214. Chusri, S., et al., *Clinical characteristics and outcomes of community and hospital-acquired Acinetobacter baumannii bacteremia.* J Microbiol Immunol Infect, 2019. **52**(5): p. 796-806.
215. Delle Rose, D., et al., *An in-depth analysis of nosocomial bloodstream infections due to Gram-negative bacilli: clinical features, microbiological characteristics and predictors of mortality in a 1 year, prospective study in a large tertiary care Italian hospital.* Infect Dis (Lond), 2019. **51**(1): p. 12-22.
216. Eliakim-Raz, N., et al., *Risk Factors for Treatment Failure and Mortality Among Hospitalized Patients With Complicated Urinary Tract Infection: A Multicenter Retrospective Cohort Study (RESCUING Study Group).* Clin Infect Dis, 2019. **68**(1): p. 29-36.
217. Gómez Belda, A.B., et al., *Inadequate empirical antimicrobial treatment in older people with bacteremic urinary tract infection who reside in nursing homes: A multicenter prospective observational study.* Geriatr Gerontol Int, 2019. **19**(11): p. 1112-1117.
218. Huang, Y.C., et al., *Comparison of clinical characteristics of bacteremia from Elizabethkingia meningoseptica and other carbapenem-resistant, non-fermenting Gram-negative bacilli at a tertiary medical center.* J Microbiol Immunol Infect, 2019. **52**(2): p. 304-311.
219. Jeon, K., et al., *Characteristics, management and clinical outcomes of patients with sepsis: a multicenter cohort study in Korea.* Acute and Critical Care, 2019. **34**(3): p. 179-191.
220. Kim, S.H., et al., *Inappropriate empirical antibiotic therapy does not adversely affect the clinical outcomes of patients with acute pyelonephritis caused by extended-spectrum beta-lactamase-producing Enterobacteriales.* European Journal of Clinical Microbiology & Infectious Diseases, 2019. **38**(5): p. 937-944.
221. Lat, I., et al., *A Multicenter, Prospective, Observational Study to Determine Predictive Factors for Multidrug-Resistant Pneumonia in Critically Ill Adults: The DEFINE Study.* Pharmacotherapy, 2019. **39**(3): p. 253-260.
222. Lim, C.L. and D. Spelman, *Mortality impact of empirical antimicrobial therapy in ESBL- and AmpC-producing Enterobacteriaceae bacteremia in an Australian tertiary hospital.* Infect Dis Health, 2019. **24**(3): p. 124-133.
223. Maruyama, T., et al., *A Therapeutic Strategy for All Pneumonia Patients: A 3-Year Prospective Multicenter Cohort Study Using Risk Factors for Multidrug-resistant Pathogens to Select Initial Empiric Therapy.* Clin Infect Dis, 2019. **68**(7): p. 1080-1088.
224. Morvan, A.C., et al., *Impact of species and antibiotic therapy of enterococcal peritonitis on 30-day mortality in critical care-an analysis of the OUTCOMEREA database.* Crit Care, 2019. **23**(1): p. 307.
225. Park, J.W., et al., *Epidemiological, clinical, and microbiological characteristics of carbapenemase-producing Enterobacteriaceae bloodstream infection in the Republic of Korea.* Antimicrob Resist Infect Control, 2019. **8**: p. 48.
226. Ramos-Rincón, J.M., et al., *The quick Sepsis-related Organ Failure Assessment (qSOFA) is a good predictor of in-hospital mortality in very elderly patients with bloodstream infections: A retrospective observational study.* Sci Rep, 2019. **9**(1): p. 15075.
227. Rodriguez-Gómez, J., et al., *Prognosis of urinary tract infection caused by KPC-producing Klebsiella pneumoniae: The impact of inappropriate empirical treatment.* J Infect, 2019. **79**(3): p. 245-252.
228. Schuttevaer, R., et al., *Appropriate empirical antibiotic therapy and mortality: Conflicting data explained by residual confounding.* PLoS One, 2019. **14**(11): p. e0225478.
229. Shi, Q., et al., *A retrospective analysis of Pseudomonas aeruginosa bloodstream infections: prevalence, risk factors, and outcome in carbapenem-susceptible and -non-susceptible infections.* Antimicrob Resist Infect Control, 2019. **8**: p. 68.
230. Tacconelli, E., et al., *Role of place of acquisition and inappropriate empirical antibiotic therapy on the outcome of extended-spectrum β-lactamase-producing Enterobacteriaceae infections.* Int J Antimicrob Agents, 2019. **54**(1): p. 49-54.

231. Wang, X., et al., *Retrospective Observational Study from a Chinese Network of the Impact of Combination Therapy versus Monotherapy on Mortality from Carbapenem-Resistant Enterobacteriaceae Bacteremia.* Antimicrob Agents Chemother, 2019. **63**(1).
232. Wiggers, J.B., et al., *The association of adequate empirical treatment and time to recovery from bacteraemic urinary tract infections: a retrospective cohort study.* Clin Microbiol Infect, 2019. **25**(10): p. 1253-1258.
233. Al-Sunaidar, K.A., N. Prof Abd Aziz, and Y. Prof Hassan, *Appropriateness of empirical antibiotics: risk factors of adult patients with sepsis in the ICU.* Int J Clin Pharm, 2020. **42**(2): p. 527-538.
234. Augustin, P., et al., *Outcome and Adequacy of Empirical Antibiotherapy in Post-Operative Peritonitis: A Retrospective Study.* Surg Infect (Larchmt), 2020. **21**(3): p. 284-292.
235. Babich, T., et al., *Risk factors for mortality among patients with Pseudomonas aeruginosa bacteraemia: a retrospective multicentre study.* Int J Antimicrob Agents, 2020. **55**(2): p. 105847.
236. Benetazzo, L., et al., *Combination Therapy with Aminoglycoside in Bacteremiasdue to ESBL-Producing Enterobacteriaceae in ICU.* Antibiotics-Basel, 2020. **9**(11): p. 13.
237. Chen, H.Y. and Y.C. Hsu, *Afebrile Bacteremia in Adult Emergency Department Patients with Liver Cirrhosis: Clinical Characteristics and Outcomes.* Sci Rep, 2020. **10**(1): p. 7617.
238. Chen, F.C., et al., *Does inappropriate initial antibiotic therapy affect in-hospital mortality of patients in the emergency department with Escherichia coli and Klebsiella pneumoniae bloodstream infections?* Int J Immunopathol Pharmacol, 2020. **34**: p. 2058738420942375.
239. Dubler, S., et al., *Does vancomycin resistance increase mortality in Enterococcus faecium bacteraemia after orthotopic liver transplantation? A retrospective study.* Antimicrob Resist Infect Control, 2020. **9**(1): p. 22.
240. Falcone, M., et al., *Time to appropriate antibiotic therapy is a predictor of outcome in patients with bloodstream infection caused by KPC-producing Klebsiella pneumoniae.* Crit Care, 2020. **24**(1): p. 29.
241. Ingram, P.R., et al., *Impact of adherence to surgical and non-surgical components of infective endocarditis guidelines and recommendations.* J Infect Chemother, 2020. **26**(9): p. 923-927.
242. Kang, F.Y., et al., *Influence of severity of infection on the effect of appropriate antimicrobial therapy for Acinetobacter baumannii bacteremic pneumonia.* Antimicrob Resist Infect Control, 2020. **9**(1): p. 160.
243. Kawasuji, H., et al., *Proactive infectious disease consultation at the time of blood culture collection is associated with decreased mortality in patients with methicillin-resistant Staphylococcus aureus bacteremia: A retrospective cohort study.* J Infect Chemother, 2020. **26**(6): p. 588-595.
244. Kim, D.H., et al., *The Use of Inappropriate Antibiotics in Patients Admitted to Intensive Care Units with Nursing Home-Acquired Pneumonia at a Korean Teaching Hospital.* Tuberculosis and Respiratory Diseases, 2020. **83**(1): p. 81-88.
245. Lambregts, M.M.C., et al., *Mortality After Delay of Adequate Empiric Antimicrobial Treatment of Bloodstream Infection.* Journal of Clinical Medicine, 2020. **9**(5): p. 10.
246. Lee, C.C., et al., *Is Early Oral Antimicrobial Switch Useful for Less Critically Ill Adults with Community-Onset Bacteraemia in Emergency Departments?* Antibiotics (Basel), 2020. **9**(11).
247. Lee, C.H., et al., *Differential impacts of inappropriate empirical therapy on ED patients with Staphylococcus aureus and streptococci bacteremia.* Am J Emerg Med, 2020. **38**(5): p. 940-946.
248. Malbasa, J.D., et al., *Risk factors and clinical outcomes for intensive care unit patients with multidrug-resistant Acinetobacter spp. bacteremia.* Hippokratia, 2020. **24**(1): p. 21-26.
249. Martinez-Nadal, G., et al., *Inappropriate Empirical Antibiotic Treatment in High-risk Neutropenic Patients With Bacteremia in the Era of Multidrug Resistance.* Clin Infect Dis, 2020. **70**(6): p. 1068-1074.

250. Mitsuboshi, S., et al., *Advanced Age is not a Risk Factor for Mortality in Patients with Bacteremia Caused by Extended-Spectrum β-Lactamase-Producing Organisms: a Multicenter Cohort Study.* Jpn J Infect Dis, 2020. **73**(4): p. 288-292.
251. Montero, M.M., et al., *Risk Factors for Mortality among Patients with Pseudomonas aeruginosa Bloodstream Infections: What Is the Influence of XDR Phenotype on Outcomes?* Journal of Clinical Medicine, 2020. **9**(2): p. 14.
252. Mora-Guzmán, I., et al., *Surgical site infection by carbapenemase-producing Enterobacteriaceae. A challenge for today's surgeons.* Cir Esp (Engl Ed), 2020. **98**(6): p. 342-349.
253. Papadimitriou-Olivgeris, M., et al., *Molecular characteristics and predictors of mortality among Gram-positive bacteria isolated from bloodstream infections in critically ill patients during a 5-year period (2012-2016).* Eur J Clin Microbiol Infect Dis, 2020. **39**(5): p. 863-869.
254. Rhee, C., et al., *Prevalence of Antibiotic-Resistant Pathogens in Culture-Proven Sepsis and Outcomes Associated With Inadequate and Broad-Spectrum Empiric Antibiotic Use.* JAMA Netw Open, 2020. **3**(4): p. e202899.
255. Righolt, C.H., P. Lagace-Wiens, and S.M. Mahmud, *Prevalence, predictors, and consequences of inappropriate empiric antimicrobial therapy for complicated urinary tract and intra-abdominal infections in Winnipeg hospitals.* Diagnostic Microbiology and Infectious Disease, 2020. **96**(1): p. 6.
256. Rivera-Espinar, F., et al., *Impact of KPC Production and High-Level Meropenem Resistance on All-Cause Mortality of Ventilator-Associated Pneumonia in Association with Klebsiella pneumoniae.* Antimicrob Agents Chemother, 2020. **64**(6).
257. Santos, S., et al., *Sepsis - Retrospective Observational Study of Sepsis and Septic Shock treated in internal medicine wards.* Galicia Clinica, 2020. **81**(3): p. 70-74.
258. Seo, H., et al., *Clinical and Microbiological Analysis of Risk Factors for Mortality in Patients with Carbapenem-Resistant Enterobacteriaceae Bacteremia.* Int J Antimicrob Agents, 2020. **56**(4): p. 106126.
259. Seok, H., et al., *Timing of antibiotics in septic patients: a prospective cohort study.* Clin Microbiol Infect, 2020. **26**(11): p. 1495-1500.
260. Wagner, J.L., et al., *Antimicrobial Stewardship Metrics that Matter.* Infectious Diseases in Clinical Practice, 2020. **28**(2): p. 89-93.
261. Wang, Y.C., et al., *Is Polymicrobial Bacteremia an Independent Risk Factor for Mortality in Acinetobacter baumannii Bacteremia?* Journal of Clinical Medicine, 2020. **9**(1): p. 10.
262. Wiener-Well, Y., et al., *Prospective Audit of Empirical Antibiotic Therapy for Septic Patients.* Isr Med Assoc J, 2020. **22**(6): p. 378-383.
263. Xiao, T., et al., *A Retrospective Analysis of Risk Factors and Outcomes of Carbapenem-Resistant Klebsiella pneumoniae Bacteremia in Nontransplant Patients.* J Infect Dis, 2020. **221**(Suppl 2): p. S174-s183.
264. Zhao, Y., et al., *Risk Factors and Outcomes of Antibiotic-resistant Pseudomonas aeruginosa Bloodstream Infection in Adult Patients With Acute Leukemia.* Clin Infect Dis, 2020. **71**(Suppl 4): p. S386-s393.
265. Aliyu, S., et al., *Prevalence and Outcomes of Multi-Drug Resistant Blood Stream Infections Among Nursing Home Residents Admitted to an Acute Care Hospital.* Journal of Intensive Care Medicine, 2021: p. 7.
266. Amipara, R., et al., *Impact of follow up blood cultures on outcomes of patients with community-onset gram-negative bloodstream infection.* Eclinicalmedicine, 2021. **34**: p. 7.
267. Cetin, S., et al., *Comparison of Risk Factors and Outcomes in Carbapenem-Resistant and Carbapenem-Susceptible Gram-Negative Bacteremia.* Medical Bulletin of Sisli Etfal Hospital, 2021. **55**(3): p. 398-404.
268. Chang, Y., et al., *The Distribution of Multidrug-resistant Microorganisms and Treatment Status of Hospital-acquired Pneumonia/Ventilator-associated Pneumonia in Adult Intensive*

Care Units: a Prospective Cohort Observational Study. Journal of Korean Medical Science, 2021. **36**(41): p. 15.
269. D'Onofrio, V., et al., *Audit of empirical antibiotic therapy for sepsis and the impact of early multidisciplinary consultation on patient outcomes.* Int J Antimicrob Agents, 2021. **58**(3): p. 106379.
270. Gomez-Zorrilla, S., et al., *A Large Multicenter Prospective Study of Community-Onset Healthcare Associated Bacteremic Urinary Tract Infections in the Era of Multidrug Resistance: Even Worse than Hospital Acquired Infections?* Infectious Diseases and Therapy, 2021. **10**(4): p. 2677-2699.
271. Jovanovic, B., et al., *Trauma and Antimicrobial Resistance Are Independent Predictors of Inadequate Empirical Antimicrobial Treatment of Ventilator-Associated Pneumonia in Critically Ill Patients.* Surgical Infections, 2021. **22**(7): p. 730-737.
272. Kadri, S.S., et al., *Inappropriate empirical antibiotic therapy for bloodstream infections based on discordant in-vitro susceptibilities: a retrospective cohort analysis of prevalence, predictors, and mortality risk in US hospitals.* Lancet Infect Dis, 2021. **21**(2): p. 241-251.
273. Kohler, P., et al., *Temporal trends, risk factors and outcomes of infections due to extended-spectrum beta-lactamase producing Enterobacterales in Swiss solid organ transplant recipients between 2012 and 2018.* Antimicrobial Resistance and Infection Control, 2021. **10**(1): p. 12.
274. Liu, K.S., et al., *Risk Factors of 30-Day All-Cause Mortality in Patients with Carbapenem-Resistant Klebsiella pneumoniae Bloodstream Infection.* Journal of Personalized Medicine, 2021. **11**(7): p. 11.
275. Man, M.Y., et al., *Impact of appropriate empirical antibiotics on clinical outcomes in Klebsiella pneumoniae bacteraemia.* Hong Kong Medical Journal, 2021. **27**(4): p. 247-257.
276. Meng, X., et al., *Ten-Year Changes in Bloodstream Infection With Acinetobacter Baumannii Complex in Intensive Care Units in Eastern China: A Retrospective Cohort Study.* Frontiers in Medicine, 2021. **8**: p. 11.
277. Moschou, A., et al., *A prospective study of epidemiology characteristics and outcomes of bloodstream infections in older patients.* Australas J Ageing, 2021. **40**(3): p. e182-e189.
278. Puzniak, L., et al., *Effect of Inadequate Empiric Antibacterial Therapy on Hospital Outcomes in SARS-CoV-2-Positive and -Negative US Patients With a Positive Bacterial Culture: A Multicenter Evaluation From March to November 2020.* Open Forum Infectious Diseases, 2021. **8**(6): p. 9.
279. Quillici, M.C.B., et al., *Gram-negative bacilli bacteremia: a 7 year retrospective study in a referral Brazilian tertiary-care teaching hospital.* J Med Microbiol, 2021. **70**(1).
280. Rodríguez, O.L., et al., *Mortality-related factors in patients with OXA-48 carbapenemase-producing Klebsiella pneumoniae bacteremia.* Medicine (Baltimore), 2021. **100**(14): p. e24880.
281. Shen, L., et al., *Bloodstream Infections due to Carbapenem-Resistant Klebsiella pneumoniae: A Single-Center Retrospective Study on Risk Factors and Therapy Options.* Microb Drug Resist, 2021. **27**(2): p. 227-233.
282. Shorr, A.F., et al., *Pneumococal community-acquired pneumonia in the intensive care unit: Azithromycin remains protective despite macrolide resistance.* Respiratory Medicine, 2021. **177**: p. 5.
283. Sun, Y., et al., *Taxonomy, virulence determinants and antimicrobial susceptibility of Aeromonas spp. isolated from bacteremia in southeastern China.* Antimicrob Resist Infect Control, 2021. **10**(1): p. 43.
284. Teelucksingh, K. and E. Shaw, *Clinical characteristics, appropriateness of empiric antibiotic therapy, and outcome of Pseudomonas aeruginosa bacteremia across multiple community hospitals.* European Journal of Clinical Microbiology & Infectious Diseases, 2021: p. 10.
285. Thy, M., et al., *Dynamic Changes in Microbial Composition During Necrotizing Soft-Tissue Infections in ICU Patients.* Front Med (Lausanne), 2020. **7**: p. 609497.

286. Yiang, G.T., et al., *Early Screening of Risk for Multidrug-Resistant Organisms in the Emergency Department in Patients With Pneumonia and Early Septic Shock: Single-Center, Retrospective Cohort Study.* Shock, 2021. **55**(2): p. 198-209.
287. Zhang, Q., et al., *A TTP-incorporated scoring model for predicting mortality of solid tumor patients with bloodstream infection caused by Escherichia coli.* Supportive Care in Cancer, 2021: p. 9.
288. Zhu, H., et al., *Impact of inappropriate empirical antibiotic treatment on clinical outcomes of urinary tract infections caused by Escherichia coli: a retrospective cohort study.* J Glob Antimicrob Resist, 2021. **26**: p. 148-153.
289. Zilberberg, M.D., et al., *Multiple antimicrobial resistance and outcomes among hospitalized patients with complicated urinary tract infections in the US, 2013-2018: a retrospective cohort study.* BMC Infect Dis, 2021. **21**(1): p. 159.
